# Interventions for quitting vaping

**DOI:** 10.1002/14651858.CD016058.pub2

**Published:** 2025-01-08

**Authors:** Ailsa R Butler, Nicola Lindson, Jonathan Livingstone-Banks, Caitlin Notley, Tari Turner, Nancy A Rigotti, Thomas R Fanshawe, Lynne Dawkins, Rachna Begh, Angela Difeng Wu, Leonie Brose, Monserrat Conde, Erikas Simonavičius, Jamie Hartmann-Boyce

**Affiliations:** Nuffield Department of Primary Care Health SciencesUniversity of OxfordOxfordUK; Addiction Research Group, Norwich Medical SchoolUniversity of East AngliaNorwichUK; Cochrane AustraliaSchool of Public Health & Preventive MedicineMelbourneAustralia; Tobacco Research and Treatment Center, Department of MedicineMassachusetts General Hospital and Harvard Medical SchoolBostonMassachusettsUSA; Nicotine, Tobacco and Vaping Research GroupLondon South Bank UniversityLondonUK; Department of Addiction SciencesKing's College LondonLondonUK; Department of Health Promotion and PolicyUniversity of MassachusettsAmherstMAUSA

**Keywords:** Adolescent, Humans, Young Adult, Bias, Electronic Nicotine Delivery Systems, Nicotine, Nicotine/administration & dosage, Nicotinic Agonists, Nicotinic Agonists/therapeutic use, Randomized Controlled Trials as Topic, Smoking Cessation, Smoking Cessation/methods, Tobacco Use Cessation Devices, Tobacco Use Cessation Devices/adverse effects, Vaping, Vaping/adverse effects, Vaping/prevention & control

## Abstract

**Rationale:**

There is limited guidance on the best ways to stop using nicotine‐containing vapes (otherwise known as e‐cigarettes) and ensure long‐term abstinence, whilst minimising the risk of tobacco smoking and other unintended consequences. Treatments could include pharmacological interventions, behavioural interventions, or both.

**Objectives:**

To conduct a living systematic review assessing the benefits and harms of interventions to help people stop vaping compared to each other or to placebo or no intervention.

To also assess how these interventions affect the use of combustible tobacco, and whether the effects vary based on participant characteristics.

**Search methods:**

We searched the following databases from 1 January 2004 to 24 April 2024: CENTRAL; MEDLINE; Embase; PsycINFO; ClinicalTrials.gov (through CENTRAL); World Health Organization International Clinical Trials Registry Platform (through CENTRAL). We also searched the references of eligible studies and abstracts from the Society for Research on Nicotine and Tobacco 2024 conference, and contacted study authors.

**Eligibility criteria:**

Randomised controlled trials (RCTs) recruiting people of any age using nicotine‐containing vapes, regardless of tobacco smoking status. Studies had to test an intervention designed to support people to quit vaping, and plan to measure at least one of our outcomes.

**Outcomes:**

Critical outcomes: vaping cessation; change in combustible tobacco use at six months or longer; number of participants reporting serious adverse events (SAEs) at one week or longer.

**Risk of bias:**

We used the Cochrane RoB 1 tool to assess bias in the included studies.

**Synthesis methods:**

We followed standard Cochrane methods for screening and data extraction. We grouped studies by comparisons and outcomes reported, and calculated individual study and pooled effects, as appropriate. We used random‐effects Mantel‐Haenszel methods to calculate risk ratios (RR) with 95% confidence intervals (CI) for dichotomous outcomes. We used random‐effects inverse variance methods to calculate mean differences and 95% CI for continuous outcomes. We assessed the certainty of the evidence using the GRADE approach.

**Included studies:**

Nine RCTs, representing 5209 participants motivated to stop using nicotine‐containing vapes at baseline, are included. In six studies, participants were abstinent from smoking tobacco cigarettes at baseline, although most studies included some participants who had previously smoked. Eight studies included participants aged 18 or older, three included only young adults (18 to 24 years), and one included 13‐ to 17‐year‐olds only. We judged three studies at low risk, three at high risk, and three at unclear risk of bias.

**Synthesis of results:**

**Pharmacological interventions for quitting nicotine vaping**

Studies assessed combination nicotine replacement therapy (NRT), cytisine, and varenicline as pharmacological interventions for quitting vaping in comparison to placebo or no/minimal support (control). The point estimate for combination NRT indicated possible benefit, but the CI incorporated the possibility of no benefit and a potential benefit of control (very low‐certainty evidence due to imprecision and risk of bias; RR 2.57, 95% CI 0.29 to 22.93; 1 study, 16 participants). The one study investigating cytisine did not report vaping cessation rates at six months or longer. Varenicline increased vaping cessation rates at six months, but the evidence was low certainty due to imprecision (RR 2.00, 95% CI 1.09 to 3.68; 1 study, 140 participants).

Zero participants reported SAEs in the studies of combination NRT versus no/minimal support (1 study, 508 participants; low‐certainty evidence due to imprecision) and cytisine versus placebo (1 study, 159 participants; low‐certainty evidence due to imprecision). Three studies investigating varenicline measured the number of participants reporting SAEs. However, only one study reported an SAE (in the intervention arm); therefore, the effect estimate was calculated based on that single study (RR 2.60, 95% CI 0.11 to 62.16; 95 participants; low‐certainty evidence due to imprecision).

**Behavioural interventions for quitting nicotine vaping**

Studies assessed reducing nicotine concentration and vaping behaviour (1 study) and text message‐based interventions (3 studies) as behavioural interventions for stopping vaping in comparison to no/minimal support (control). In one study, the point estimate suggested nicotine/vaping reduction increased vaping cessation compared to minimal support at six‐month follow‐up, but the CI incorporated the possibility of no intervention effect and higher cessation rates in the control arm (RR 3.38, 95% CI 0.43 to 26.30; 17 participants; very low‐certainty due to imprecision and risk of bias). There was low‐certainty evidence (downgraded two levels due to indirectness) that text message‐based interventions may have increased vaping cessation rates compared to control in 13‐ to 24‐year‐olds (RR 1.32, 95% CI 1.19 to 1.47; I^2^ = 0%; 2 studies, 4091 participants).

The one study investigating nicotine/vaping behaviour reduction did not report on SAEs. One of the studies investigating text message‐based interventions did report on SAEs; however, zero events were reported in both study arms (508 participants; low‐certainty evidence due to imprecision).

No studies reported change in combustible tobacco smoking at six‐month follow‐up or longer.

**Authors' conclusions:**

There is low‐certainty evidence that text message‐based interventions designed to help people stop nicotine vaping may help more youth and young adults to successfully stop than no/minimal support, and low‐certainty evidence that varenicline may also help people quit vaping. Data exploring the effectiveness of combination NRT, cytisine, and nicotine/vaping behaviour reduction are inconclusive due to risk of bias and imprecision.

Most studies that measured SAEs reported none; however, more data are needed to draw clear conclusions. Of note, data from studies investigating these interventions for quitting smoking have not demonstrated serious concerns about SAEs. No studies assessed the change in combustible tobacco smoking, including relapse to or uptake of tobacco smoking, at six‐month follow‐up or longer. It is important that future studies measure this so the complete risk profile of relevant interventions can be considered.

We identified 20 ongoing RCTs. Their incorporation into the evidence base and the continued identification of new studies is imperative to inform clinical and policy guidance on the best ways to stop vaping. Therefore, we will continue to update this review as a living systematic review by running searches monthly and updating the review when relevant new evidence that will strengthen or change our conclusions emerges.

**Funding:**

Cancer Research UK (PRCPJT‐Nov22/100012).

National Institute of Health Research (NIHR206123)

**Registration:**

Protocol available via DOI: 10.1002/14651858.CD016058.

## Plain language summary

**What are the best ways to help people to stop vaping nicotine and do they have any unwanted effects?**

**Key messages**

- Text message‐based interventions compared to no or minimal support may help young people stop vaping, and varenicline compared to no or minimal support may help people stop vaping; however, more evidence is needed.
- We need more information on whether other interventions can help people to stop vaping.
- We need more information on potential harms of interventions and whether they affect the number of people smoking tobacco.

**What are nicotine‐containing vapes?**

Vapes (also known as electronic cigarettes or e‐cigarettes) are handheld devices that heat liquid that usually contains nicotine and flavourings. Liquids are stored in a disposable or refillable cartridge, reservoir, or 'pod'. Vapes allow users to inhale nicotine in a vapour rather than smoke. Because they do not burn tobacco, vapes expose users to fewer of the substances that cause diseases in people who smoke tobacco cigarettes. However, vaping is likely to cause more harm than not vaping. Some people vape nicotine to help them quit smoking; however, some people who vape nicotine have never smoked. People may want to stop using vapes containing nicotine, but find it difficult due to the addictive properties of nicotine.

**What interventions might help people to stop vaping?**

Medicines including nicotine replacement therapy (gums, patches, etc.), varenicline, bupropion, and cytisine could be used to stop vaping. These are already used to help people stop smoking. Behavioural interventions could include counselling, text message, or online support, vaping reduction programmes and print‐based information. In this review we looked at both medicines and behavioural interventions.

**What did we want to find out?**

There is limited advice available on the best ways to stop nicotine vaping. Therefore, we brought together up‐to‐date information to see what tools have been tested for this purpose and whether they can help people to stop vaping. We also looked at any harms these interventions may cause, including whether they could cause more people to smoke tobacco.

**What did we do?**

We searched for studies looking at any intervention designed to support people who use nicotine‐containing vapes to stop vaping.

We included randomised controlled trials, in which the treatments people received were decided at random. This type of study usually gives the most reliable evidence about treatment effects.

We were interested in finding out:

- how many people stopped using nicotine vapes at least six months after study start;
- any change in tobacco smoking at least six months after study start;
- how many people experienced unwanted effects of treatment, at least one week after treatment started.

**What did we find?**

We found nine studies, including 5209 participants who used nicotine‐containing vapes. Eight studies took place in the USA and one in Italy. Treatments used to help people stop vaping were:

- two types of nicotine replacement therapy used together, such as patches and gum (otherwise known as combination nicotine replacement therapy);
- cytisine (a medicine to help people stop smoking);
- varenicline (a medicine to help people stop smoking);
- a plan to help people reduce the nicotine in their vapes and the amount they were vaping over time;
- a text message‐based intervention.

**What are the results of our review?**

Text message‐based interventions might help more young people (13 to 24 years) stop vaping compared to no support or minimal support. Varenicline may also help people quit vaping. There was not enough information to say whether any of the other interventions were better at helping people to stop vaping than no or minimal support.

There was not enough information about the potential harms of the treatments, as very few studies reported any serious unwanted effects. No studies looked at whether the interventions studied affected the number of people smoking tobacco after six months.

**How reliable are these results?**

Our results are based on small numbers of studies and participants for most outcomes, making it difficult to draw conclusions.

We found some evidence that youth and young adults may be more likely to stop vaping nicotine using a text message‐based intervention than using no or minimal support. However, both studies looking at this tested the same text message intervention. We do not know whether we would find the same results for other text message‐based interventions or in older adults. We also found some evidence that varenicline may help more people to stop vaping nicotine than no or minimal support. However, more evidence is needed, which could change our conclusions.

The studies looking at whether people experienced serious unwanted effects either reported none or very few, making it difficult to draw clear conclusions about whether more serious unwanted effects occur when using particular interventions. More studies and participants will help us feel more confident about this.

In some cases, only one study tested an intervention and measured an outcome. Where there were issues with the quality of the study, this made it difficult to draw conclusions about which interventions work best and their safety.

More relevant studies are underway and will help us learn more about what interventions could be used to help people to stop vaping nicotine.

**How up‐to‐date is this evidence?**

We included evidence published up to 24 April 2024. We will run monthly searches and update this review when new information will strengthen or change our conclusions.

## Summary of findings

**Summary of findings 1:** Summary of findings table ‐ Combination NRT compared to control for nicotine vaping cessation

**Combination NRT compared to control for nicotine vaping cessation**						
**Patient or population:** nicotine vaping cessation **Setting:** Any (USA) **Intervention:** combination NRT **Comparison:** control						
Outcomes	Anticipated absolute effects^*^ (95% CI)		Relative effect (95% CI)	№ of participants (studies)	Certainty of the evidence (GRADE)	Comments
	Risk with control	Risk with combination NRT				
Vaping cessation at 6 months or longer follow‐up: 6 months	11 per 100	**29 per 100** (3 to 100)	**RR 2.57** (0.29 to 22.93)	16 (1 RCT)	⊕⊝⊝⊝ Very low^a,^^b^	
Change in combustible tobacco use at 6 months or longer ‐ not reported	‐	‐	‐	‐	‐	No studies reported this outcome.
Number of participants reporting SAEs follow‐up: 3 months	Not pooled	Not pooled	Not pooled	508 (1 RCT)	⊕⊕⊝⊝ Low^c^	We did not calculate relative or absolute effects as there were no events across study arms.
*The risk in the intervention group (and its 95% confidence interval) is based on the assumed risk in the comparison group and the **relative effect** of the intervention (and its 95% CI). **CI:** confidence interval; **RR:** risk ratio						
**GRADE Working Group grades of evidence** **High certainty:** we are very confident that the true effect lies close to that of the estimate of the effect. **Moderate certainty:** we are moderately confident in the effect estimate: the true effect is likely to be close to the estimate of the effect, but there is a possibility that it is substantially different. **Low certainty:** our confidence in the effect estimate is limited: the true effect may be substantially different from the estimate of the effect. **Very low certainty:** we have very little confidence in the effect estimate: the true effect is likely to be substantially different from the estimate of effect.						
See interactive version of this table: https://gdt.gradepro.org/presentations/#/isof/isof_question_revman_web_451184852882794857.						

^a^ Downgraded two levels due to risk of bias: only study contributing to comparison and outcome was judged to be at high risk of bias. ^b^ Downgraded two levels due to imprecision: extremely low number of events across arms (n = 3) and 95% CI incorporates the potential for benefit, harm, and no effect of the intervention. ^c^ Downgraded two levels due to imprecision: no events recorded across study arms.

**Summary of findings 2:** Summary of findings table ‐ Cytisine compared to placebo for nicotine vaping cessation

**Cytisine compared to placebo for nicotine vaping cessation**						
**Patient or population:** nicotine vaping cessation **Setting:** Any (USA) **Intervention:** cytisine **Comparison:** placebo						
Outcomes	Anticipated absolute effects^*^ (95% CI)		Relative effect (95% CI)	№ of participants (studies)	Certainty of the evidence (GRADE)	Comments
	Risk with placebo	Risk with cytisine				
Vaping cessation at 6 months or longer ‐ not reported	‐	‐	‐	‐	‐	No studies reported this outcome.
Change in combustible tobacco use at 6 months or longer ‐ not reported	‐	‐	‐	‐	‐	No studies reported this outcome.
Number of participants reporting SAEs follow‐up: 4 months	Not pooled	Not pooled	Not pooled	159 (1 RCT)	⊕⊕⊝⊝ Low^a^	We did not calculate relative or absolute effects as there were no events across study arms.
*The risk in the intervention group (and its 95% confidence interval) is based on the assumed risk in the comparison group and the **relative effect** of the intervention (and its 95% CI). **CI:** confidence interval						
**GRADE Working Group grades of evidence** **High certainty:** we are very confident that the true effect lies close to that of the estimate of the effect. **Moderate certainty:** we are moderately confident in the effect estimate: the true effect is likely to be close to the estimate of the effect, but there is a possibility that it is substantially different. **Low certainty:** our confidence in the effect estimate is limited: the true effect may be substantially different from the estimate of the effect. **Very low certainty:** we have very little confidence in the effect estimate: the true effect is likely to be substantially different from the estimate of effect.						
See interactive version of this table: https://gdt.gradepro.org/presentations/#/isof/isof_question_revman_web_451185324505541376.						

^a^ Downgraded two levels due to imprecision. No events were reported across study arms.

**Summary of findings 3:** Summary of findings table ‐ Varenicline compared to control for nicotine vaping cessation

**Varenicline compared to control for nicotine vaping cessation**						
**Patient or population:** nicotine vaping cessation **Setting:** any (Italy and USA) **Intervention:** varenicline **Comparison:** control						
Outcomes	Anticipated absolute effects^*^ (95% CI)		Relative effect (95% CI)	№ of participants (studies)	Certainty of the evidence (GRADE)	Comments
	Risk with control	Risk with varenicline				
Vaping cessation at 6 months or longer follow‐up: 6 months	24 per 100	**49 per 100** (26 to 89)	**RR 2.00** (1.09 to 3.68)	140 (1 RCT)	⊕⊕⊝⊝ Low^a^	
Change in combustible tobacco use at 6 months or longer ‐ not reported	‐	‐	‐	‐	‐	No studies reported this outcome.
Number of participants reporting SAEs follow‐up: range 3 months to 6 months	Absolute effects: n/a (the one study contributing to this comparison that reported events did not report events in the control arm, so an accurate absolute risk for the treatment group could not be calculated) RR 2.60 (95% CI 0.11 to 62.16)			130 (3 RCTs)	⊕⊕⊝⊝ Low^b^	Two of the three studies in this comparison reporting SAEs reported zero events in both arms and so only one study with 95 participants contributes to the effect estimate.
*The risk in the intervention group (and its 95% confidence interval) is based on the assumed risk in the comparison group and the **relative effect** of the intervention (and its 95% CI). **CI:** confidence interval; **RR:** risk ratio						
**GRADE Working Group grades of evidence** **High certainty:** we are very confident that the true effect lies close to that of the estimate of the effect. **Moderate certainty:** we are moderately confident in the effect estimate: the true effect is likely to be close to the estimate of the effect, but there is a possibility that it is substantially different. **Low certainty:** our confidence in the effect estimate is limited: the true effect may be substantially different from the estimate of the effect. **Very low certainty:** we have very little confidence in the effect estimate: the true effect is likely to be substantially different from the estimate of effect.						
See interactive version of this table: https://gdt.gradepro.org/presentations/#/isof/isof_question_revman_web_451184323175229067.						

^a^ Downgraded two levels due to imprecision: small number of events (n = 36) reported across study arms. ^b^ Downgraded two levels due to imprecision: very few events and 95% CI incorporates the potential for benefit, harm, and no effect of the intervention.

**Summary of findings 4:** Summary of findings table ‐ Nicotine/vaping reduction compared to minimal support for nicotine vaping cessation

**Nicotine/vaping reduction compared to minimal support for nicotine vaping cessation**						
**Patient or population:** nicotine vaping cessation **Setting:** university (USA) **Intervention:** nicotine/vaping reduction **Comparison:** minimal support						
Outcomes	Anticipated absolute effects^*^ (95% CI)		Relative effect (95% CI)	№ of participants (studies)	Certainty of the evidence (GRADE)	Comments
	Risk with minimal support	Risk with nicotine/vaping reduction				
Vaping cessation at 6 months or longer follow‐up: 6 months	11 per 100	**38 per 100** (5 to 100)	**RR 3.38** (0.43 to 26.30)	17 (1 RCT)	⊕⊝⊝⊝ Very low^a,^^b^	
Change in combustible tobacco use at 6 months or longer ‐ not reported	‐	‐	‐	‐	‐	No studies reported this outcome.
Number of participants reporting SAEs ‐ not reported	‐	‐	‐	‐	‐	No studies reported this outcome.
*The risk in the intervention group (and its 95% confidence interval) is based on the assumed risk in the comparison group and the **relative effect** of the intervention (and its 95% CI). **CI:** confidence interval; **RR:** risk ratio						
**GRADE Working Group grades of evidence** **High certainty:** we are very confident that the true effect lies close to that of the estimate of the effect. **Moderate certainty:** we are moderately confident in the effect estimate: the true effect is likely to be close to the estimate of the effect, but there is a possibility that it is substantially different. **Low certainty:** our confidence in the effect estimate is limited: the true effect may be substantially different from the estimate of the effect. **Very low certainty:** we have very little confidence in the effect estimate: the true effect is likely to be substantially different from the estimate of effect.						
See interactive version of this table: https://gdt.gradepro.org/presentations/#/isof/isof_question_revman_web_451185156225608261.						

^a^ Downgraded two levels due to risk of bias: the only study contributing to the comparison and outcome was judged to be at high risk of bias. ^b^ Downgraded two levels due to imprecision: extremely low number of events across study arms and 95% CI encompasses the potential for benefit, harm, and no effect of the intervention.

**Summary of findings 5:** Summary of findings table ‐ Text message‐based interventions compared to no/minimal support for nicotine vaping cessation in young people (13 to 24 years)

**Text message‐based interventions compared to no/minimal support for nicotine vaping cessation in young people (13 to 24 years)**						
**Patient or population:** nicotine vaping cessation in young people (13 to 24 years) **Setting:** any (USA) **Intervention:** text message‐based interventions **Comparison:** no/minimal support						
Outcomes	Anticipated absolute effects^*^ (95% CI)		Relative effect (95% CI)	№ of participants (studies)	Certainty of the evidence (GRADE)	Comments
	Risk with no/minimal support	Risk with text message‐based interventions				
Vaping cessation at 6 months or longer follow‐up: 7 months	22 per 100	**29 per 100** (26 to 32)	**RR 1.32** (1.19 to 1.47)	4091 (2 RCTs)	⊕⊕⊝⊝ Low^a,^^b^	
Change in combustible tobacco use at 6 months or longer ‐ not reported	‐	‐	‐	‐	‐	No studies reported this outcome.
Number of participants reporting SAEs follow‐up: 3 months	Not pooled	Not pooled	Not pooled	508 (1 RCT)	⊕⊕⊝⊝ Low^c^	We did not calculate relative or absolute effects as there were no events across study arms.
*The risk in the intervention group (and its 95% confidence interval) is based on the assumed risk in the comparison group and the **relative effect** of the intervention (and its 95% CI). **CI:** confidence interval; **RR:** risk ratio						
**GRADE Working Group grades of evidence** **High certainty:** we are very confident that the true effect lies close to that of the estimate of the effect. **Moderate certainty:** we are moderately confident in the effect estimate: the true effect is likely to be close to the estimate of the effect, but there is a possibility that it is substantially different. **Low certainty:** our confidence in the effect estimate is limited: the true effect may be substantially different from the estimate of the effect. **Very low certainty:** we have very little confidence in the effect estimate: the true effect is likely to be substantially different from the estimate of effect.						
See interactive version of this table: https://gdt.gradepro.org/presentations/#/isof/isof_question_revman_web_451185315991890681.						

^a^ Not downgraded due to risk of bias; one of the two studies was unpublished at the time of writing and was judged to be at unclear risk of bias due to insufficient data with which to judge some domains. The other study was judged at low risk across all domains assessed, and there was no evidence of a difference between study results. ^b^ Downgraded two levels due to indirectness: the two contributing studies tested the same intervention in a relatively homogenous population. Unclear if the effects can be generalised to other text message‐based interventions and other populations ^c^ Downgraded two levels due to imprecision. No events were recorded across study arms.

## Background

### Description of the condition

Vapes or electronic cigarettes are handheld electronic devices that produce an aerosol by heating an e‐liquid [1]. The e‐liquid, usually comprising propylene glycol (a synthetic liquid substance that absorbs water) and/or glycerol (a naturally occurring alcohol), with or without nicotine and flavours, is stored in disposable or refillable cartridges or a reservoir or 'pod' [2]. Nicotine‐containing vapes or electronic cigarettes are considered less harmful to health than tobacco cigarettes, and in some countries are endorsed as smoking cessation aids [3, 4, 5, 6]. However, there are concerns about their potential harm to health if used long‐term by people who stopped smoking a long time ago or by people who have never smoked, with particular concerns relating to young people [7, 8, 9, 10]. Young people who take up nicotine vaping who have never smoked may develop a dependence on nicotine, which has led to concerns that they may be more likely to try other more harmful nicotine‐containing products, such as combustible tobacco cigarettes [11, 12, 13]. We do not yet have any evidence on the long‐term harms of nicotine vaping in the absence of a tobacco smoking history; therefore, potential health harms of vaping itself are an additional, as yet unquantified, concern. Even if modest in comparison to smoking tobacco, it is unlikely that vaping nicotine will be completely risk or harm free. Consequently, there are clear reasons to support people to stop nicotine vaping. There are also various reasons that people (of any age) who have used vapes for tobacco smoking cessation may ultimately want to stop using them. Commonly cited reasons include cost, concerns around health, perceptions of friends and family, concerns about dependence on nicotine, and stigma [14]. However, as nicotine is an addictive substance, and advancements in vaping technology make it increasingly effective at delivering nicotine to the brain, it may not be easy for people to discontinue use. In addition, people who have used vapes to stop smoking need to ensure that they are no longer at risk of relapsing to smoking if they stop using vapes. Where nicotine vaping is supported as a smoking cessation aid, there is a growing awareness that support to stop using vapes may be needed once people have fully stopped smoking.

There is currently a paucity of evidence on the best methods to stop using vapes. However, as the uptake of vaping rises, more research is emerging, with a number of ongoing studies currently registered. More relevant evidence is likely to emerge in the near future, making a living systematic review approach appropriate. Our living review approach is well suited to collating and assessing the evidence from new and ongoing studies as this information emerges.

### Description of the intervention and how it might work

Treatments to support people to stop vaping may include both pharmacological and behavioural interventions. Potential pharmacological treatments include nicotine replacement therapy (gums, patches, lozenges, etc., which can be used in combination), varenicline, bupropion, and cytisine, which are already used as aids to support people to stop smoking. Behavioural stop‐smoking interventions may also be adopted or adapted to support people to stop vaping, such as in‐person or telephone‐based counselling (one‐to‐one or group‐based), print‐based support and/or incentives to stop. Alternative therapies such as hypnotherapy or acupuncture may also be tested. Additionally, intervention delivery approaches, such as text message support, smartphone apps, or online support tools, could be used. In the UK, National Institute for Health and Care Excellence (NICE) guidance recommends that the National Health Service (NHS) provide support to help people who vape to stop when they are ready to do so, but does not set out how best to achieve this [15].

When used for smoking cessation, nicotine replacement strategies offer an approach based on harm reduction principles, substituting the nicotine consumed through smoking with nicotine delivered in other forms (e.g. transdermally or across oral mucous membranes). This in turn alleviates symptoms of nicotine withdrawal and breaks associations between nicotine delivery and reward. Cytisine and varenicline (nicotine receptor partial agonists) work by blocking some of the receptors in the brain associated with nicotine addiction, thereby also reducing the rewarding effects of smoking cigarettes containing nicotine [16]. Bupropion increases dopamine release in the brain's mesolimbic pathways that are stimulated by other addictive substances [17]. It is unclear exactly how this impacts mechanisms of nicotine addiction. However, these interventions have been well tested in smoking cessation trials, with evidence of effectiveness for smoking cessation [18]. Their use for vaping cessation is in its infancy, and no clear conclusions have been drawn on their effectiveness for this purpose as yet. However, as nicotine is the common addictive substance inhaled through both smoking and vaping and is central to these pharmacotherapies' mechanisms of action, it is reasonable to assume that these interventions may also be effective in helping people to stop vaping nicotine. In licensing these pharmacotherapies for smoking cessation, their unintended effects have been taken into account and considered minimal relative to the risks of combustible tobacco smoking. As the risks of nicotine vaping are less established than for combustible tobacco smoking, it is important that the relative risks of these pharmacotherapies versus vaping are taken into account when considering them as vaping cessation aids.

Behavioural interventions, whether delivered via counselling or using digital delivery techniques, are usually based on a psychological theory of change [19]. For example, text messages may be developed to address different aspects of addictive behaviour, such as withdrawal and triggers to relapse,and thus to attempt to intervene with specific ‘behaviour change techniques’ [20]. It is generally accepted that nicotine addiction is a complex behaviour, and behavioural interventions will thus seek to address different aspects of the behaviour (e.g. motivation, self‐efficacy, beliefs) in order to influence it.

The evidence base for how alternative therapies may work is somewhat unclear, with different theories suggesting how these therapies may work for some people. Personal beliefs can be powerful drivers of behaviour, thus ‘belief’ in a therapy, the placebo effect, or being persuaded to change (e.g. through hypnotherapy), could be potential mechanisms of change for some people.

### Why it is important to do this review

There is currently limited guidance based on direct evidence on: how to stop vaping nicotine; the most effective ways to ensure long‐term vaping cessation; or minimising the risk of tobacco smoking relapse and other unintended effects of treatment. A systematic review of the evidence, including literature published to September 2021, concluded that very little interventional research had been conducted, precluding any conclusions on the benefits and harms of vaping cessation interventions [21]. A more recent review of the literature carried out in 2023 and published in 2024 concluded that there was still little evidence on the best ways to support people to quit vaping, and that further studies were required [22]. Vaping cessation is a growing area of research, with a number of trials completed in the last year and many more underway.

## Objectives

To conduct a living systematic review to assess the benefits and harms of interventions to help people stop vaping compared to each other or to placebo or no intervention.

To also assess how these interventions affect the use of combustible tobacco, and whether the effects vary based on participant characteristics.

## Methods

The living review format means that this review will be updated as new evidence becomes available that may change the existing conclusions. We conduct database searches monthly, contact authors of ongoing studies, and make our monthly search updates publicly available at www.cebm.ox.ac.uk/research/electronic‐cigarettes‐for‐smoking‐cessation‐cochrane‐living‐systematic‐review‐1. An update to the review is triggered when the accumulating evidence leads to changes in any one of the following: the direction of effect or clinical significance of the findings for one or more outcomes; the certainty (e.g. GRADE rating) of one or more outcomes; or the availability of studies investigating new settings, populations, interventions, comparisons, or outcomes. When an update is triggered, we incorporate new data into meta‐analyses and tables in RevMan software [23]. We follow the Methodological Expectations for Cochrane Intervention Reviews (MECIR) when conducting this review and PRISMA 2020 for reporting.

For full methods relating to the living status of this review, see Supplementary material 7.

### Criteria for considering studies for this review

#### Types of studies

Randomised controlled trials (RCTs) including cross‐over trials. We did not exclude studies based on year or language of publication.

#### Types of participants

People of any age, regardless of tobacco use status, using any kind of nicotine vape at baseline. 'Current vaping' was defined as per study authors, at entry into the study, and could include people concurrently smoking tobacco and vaping. We included studies that enrolled people regardless of vaping behaviour, as long as they provided a group of nicotine vape users with vaping cessation intervention(s) and collected relevant outcomes for the subset of the population considered to be current vape users.

We did not include studies conducted exclusively in people who did not vape nicotine (e.g. in people vaping tetrahydrocannabinol, or non‐nicotine vapes). Where studies did not define type of vaping, or included people who vaped both nicotine and other types of e‐liquid, we planned to include these studies, separating out and only extracting information on the nicotine vaping subgroup, where available. If separate data for this group were not available, we planned to test exclusion of the study in a sensitivity analysis. However, no such studies were included in this version of the review.

#### Types of interventions

Any intervention designed to support people who vaped to stop vaping. This could include:

- behavioural interventions of any intensity, modality, or frequency, and from any provider;
- pharmacological interventions, such as cytisine, nicotine replacement therapy (NRT), varenicline, and bupropion, of any dosage or frequency;
- changes in characteristics of vapes, such as reductions in nicotine content;
- any combination of the above interventions.

If interventions were designed to both prevent vaping in people not currently vaping and to encourage cessation in people currently vaping, we planned to include these studies if the data on people using vapes at baseline could be separated out and at least one of the outcomes of interest was reported in this subset. However, no such studies were included in this version of the review.

### Outcome measures

#### Critical outcomes

- Vaping cessation at the longest follow‐up point, at least six months from the start of the intervention, measured on an intention‐to‐treat (ITT; including all participants in their originally assigned groups) basis using the strictest definition of abstinence, preferring biochemically validated results (self‐reported outcomes confirmed using biological tests) where reported.
- Change in combustible tobacco use (smoking) between baseline and the longest follow‐up point, at least six months from the start of the intervention. Combustible tobacco use includes tobacco cigarettes, loose roll‐your‐own, cigars, cigarillos, and pipe tobacco. Dependent on smoking status at baseline, this could be continued smoking, uptake of smoking, or smoking cessation. We measured these as defined by the study authors, using the strictest definition if multiple measures were reported, e.g. preferring continuous abstinence to point prevalence abstinence, and biochemically validated over self‐reported results for smoking cessation.
- Number of participants reporting serious adverse events (SAEs) at one week or longer (as defined by the study authors). If SAEs were reported at more than one time point, we used the measure at longest follow‐up.

#### Important outcomes

- Vaping cessation at the longest follow‐up point, at three or more but less than six months from the start of the intervention, measured as per critical vaping cessation outcome.
- Change in combustible tobacco use between baseline and the longest follow‐up point, three or more but less than six months from the start of the intervention, measured as per critical change in tobacco use outcome.
- Number of participants reporting adverse events (AEs) at one week or longer (as defined by the study authors), at the longest follow‐up point reported.
- Number of people vaping a substance other than nicotine at longest follow‐up, at three months follow‐up or longer.
- Changes in weight between baseline and longest follow‐up point.
- Changes in alcohol use between baseline and longest follow‐up point.
- Changes in the following measures at longest follow‐up (one week or longer):
  - carbon monoxide (CO), as measured through breath or blood;
  - blood pressure;
  - heart rate;
  - blood oxygen saturation;
  - lung function measures;
  - cotinine;
  - known toxins/carcinogens, as measured through blood or urine (examples of toxicant names and abbreviations are listed in Appendix 2 of our review on e‐cigarettes for smoking cessation) [18].

Studies needed to plan to measure at least one of the critical or important outcomes above to be eligible for inclusion.

### Search methods for identification of studies

#### Electronic searches

We conducted searches up to 24 April 2024, searching the following databases and employing the targeted search strategy in Supplementary material 1:

- Cochrane Central Register of Controlled Trials (CENTRAL; 2024, Issue 3) via CRS‐Web;
- MEDLINE (via Ovid SP, from 1 January 2004 to 24 April 2024);
- Embase (via Ovid SP, from 1 January 2004 to 24 April 2024);
- PsycINFO (via Ovid SP, from 1 January 2004 to 24 April 2024);
- ClinicalTrials.gov (via CENTRAL; 2024, Issue 3);
- World Health Organization International Clinical Trials Registry Platform (WHO ICTRP) (via CENTRAL; 2024, Issue 3).

This initial search was limited to 2004 to present because vapes were not available before 2004. Following this initial search, we will conduct monthly searches of the same databases on the first day of each month. These monthly searches will be conducted in combination with the monthly searches for the Cochrane review of *'Electronic cigarettes for smoking cessation'* [18]. The search terms used in these searches are broad enough to retrieve studies eligible for either review, using free text and subject headings relating to vape use, alongside study design filters matching our inclusion criteria. All ongoing search strategies are listed in Supplementary material 1.

#### Searching other resources

We searched the reference lists of eligible studies found in the literature searches. We contacted authors of known and eligible studies for further information when needed. We also searched abstracts from the 2024 Society for Research on Nicotine and Tobacco (SRNT) Annual Meeting.

### Data collection and analysis

#### Selection of studies

We screened citations retrieved by our searches using Covidence [24]. Two review authors independently checked the titles and abstracts for relevance against the eligibility criteria (NL, JHB, ARB, MC, LD, LB, CN, ES). Any disagreements were resolved through discussion with a third review author. We obtained the full‐text versions of papers considered to be potentially relevant. Two review authors independently assessed the full‐text reports for inclusion in the review. Any disagreements were resolved through discussion with a third review author. Where necessary, we contacted study investigators for further information to aid our decision‐making. We recorded and reported reasons for excluding studies at the full‐text stage.

We screened and included studies reported in any language. Had it been necessary, we would have arranged for the translation of non‐English language papers. Where we found multiple citations relating to the same study, we grouped them into one study record with a single study ID.

#### Data extraction and management

For each included study, two review authors independently extracted data to be used in analyses (including covariates) and for risk of bias assessment (ARB, LB, ES, LD, NL). Study characteristics were extracted by a single review author. We cross‐checked dual extraction, with any disagreements between review authors resolved through discussion or by involving a third review author. Data extraction processes were carried out using Covidence and piloted before use. We imported extracted and checked data into RevMan software [23, 24]. See Supplementary material 8 for the information extracted from the included studies.

#### Risk of bias assessment in included studies

Two review authors independently assessed the risk of bias for each included study (NL, JHB, ARB, LB, ES, LD). We used the methods set out by the Cochrane Tobacco Addiction Group [25], which is based on the domains of the Cochrane RoB 1 tool [26]. This approach uses a domain‐based evaluation that addresses seven different areas: random sequence generation; allocation concealment; blinding of participants and providers; blinding of outcome assessment; incomplete outcome data; selective outcome reporting; and other potential sources of bias. We assigned a judgement (low, high, or unclear) for each domain. Any disagreements were resolved through discussion or by consulting a third review author when required.

Specific considerations about judgements for individual domains in this review are outlined below and are in line with our existing review of *‘Electronic cigarettes for smoking cessation’* [18].

- Blinding of participants and providers: we did not assess this domain for studies solely investigating behavioural interventions, as specific risk of bias guidance developed by the Cochrane Tobacco Addiction Group advises this, due to it being impossible to blind these types of interventions [25]. For studies of pharmacological interventions that did not use blinding, we considered studies at low risk of bias for this domain if the intervention was compared to a placebo or an active control of similar intensity, as we judge performance bias to be unlikely in this circumstance. However, if a study was unblinded, and the comparator group was a minimal‐intervention control or of lower intensity than the intervention group, we considered the study to be at high risk of bias for this domain.
- Blinding of outcome assessment: following the standard methods of the Cochrane Tobacco Addiction Group, we considered studies to be at low risk of detection bias if they assessed our primary outcome(s) objectively, or if participants received the same amount of face‐to‐face contact across relevant study groups, or both [25].
- Incomplete outcome data: again, following the standard methods of the Cochrane Tobacco Addiction Group, we rated studies at high risk of attrition bias if loss to follow‐up was greater than 50% overall, or if there was a difference in follow‐up rates of more than 20% between study arms at the longest follow‐up used in our analysis [25].

We judged studies to be at high risk of bias overall if they were rated at high risk in at least one domain, and at low risk of bias overall if they were judged to be at low risk across all domains evaluated. We judged the remaining studies to be at unclear risk of bias overall.

Where a study reported more than one of our outcomes of interest, we assessed risk of bias for our critical vaping and smoking outcomes only.

#### Measures of treatment effect

We calculated risk ratios (RR) and their 95% confidence intervals (CI; the range indicating where the true effect is likely to be) for dichotomous outcomes for each study. For continuous outcomes, we compared the difference between the relevant intervention and control groups using mean differences (MD) and 95% CI.

#### Unit of analysis issues

In the case of Klein 2024 [27, 28, 29, 30, 31, 32], for our combined NRT versus control comparison, we combined the combination NRT and combination NRT + text message intervention arms into one intervention arm, and the no/minimal support arm and the text message arm into one control arm. For Klein 2024 and our text message intervention versus control comparison, we combined the text message only intervention and the combination NRT + text message intervention into one intervention arm and the no/minimal support arm and the combination NRT arm into one control group. In both instances, this was because there was no evidence of an interaction between combination NRT and the text message‐based behavioural support. In future, we will continue to only consider combining trial arms if this is how the information is presented by study authors, or where there is no evidence of difference between similar trial arms for the outcome of interest.

None of the studies included in this review were cluster‐randomised. Had any been cluster‐randomised, we would have assessed whether study authors adjusted for clustering, and whether this had an impact on the overall result. Had clustering had little impact on the results, we would have used unadjusted quit‐rate data; however, if clustering had impacted results, we would have adjusted for this using the intraclass correlation (ICC) reported by the paper (or where this was not provided, one used in a similar study). Should we include cluster‐RCTs in the future, this is the approach we will take.

In the case of eligible cross‐over trials (ensuring that the first assignment period was sufficiently long to meet our inclusion criteria), we planned to extract and report on results at the end of the first assignment period, where these were available. At this point, we have not identified any eligible cross‐over trials, but will use this approach if we identify them in future.

#### Dealing with missing data

When assessing change in tobacco use, we used the standard Cochrane Tobacco Addiction Group approach, treating participants with missing data as still smoking. We made the same assumption for vaping when assessing whether vaping cessation had taken place, assuming those lost to follow‐up were continuing to vape.

We based the proportion of people affected by SAEs/AEs on the number of people available for follow‐up, and not the number randomised, where reported.

For continuous outcomes, we also used complete‐case data and did not attempt to impute missing values. Where possible, we extracted data demonstrating the change in the outcome between baseline and follow‐up and compared this change data between study arms. However, where this was not reported, we compared the data at follow‐up only between study arms.

#### Reporting bias assessment

As noted above, we took selective reporting into consideration as part of our risk of bias assessment for each study. When interpreting the results, we accounted for this, and also planned to account for potential findings of studies that we knew to have taken place, but for which we did not have results (however, we did not become aware of any such studies).

Reporting bias can be assessed using funnel plots, where 10 or more studies contribute to a given outcome [33]. None of our meta‐analyses included 10 or more studies. However, where 10 or more studies are included in an analysis in future updates, we will generate funnel plots.

#### Synthesis methods

We took the clinical variance of studies into account when grouping them for analyses. Studies were split into comparisons based on intervention and comparator type (e.g. studies investigating behavioural interventions were not grouped with those investigating pharmacological interventions, and different types of pharmacological interventions were grouped separately).

We carried out pairwise meta‐analyses for comparisons where there was more than one eligible RCT. We used random‐effects Mantel‐Haenszel models to calculate pooled RR with 95% CI for dichotomous outcomes. For continuous outcomes, we calculated pooled MDs, using the inverse variance approach (also with 95% CI).

Where meta‐analysis was not possible or appropriate, we synthesised data narratively and using effect direction plots [33].

#### Investigation of heterogeneity and subgroup analysis

We assessed clinical and methodological diversity between studies to guide decisions as to whether data should be pooled. Where we pooled studies using meta‐analysis, we calculated I^2^ statistics [26, 33]. We considered a value greater than 50% as evidence of substantial heterogeneity (difference between the results of studies included in the analysis). Should any I^2^ have exceeded 75%, this would have been deemed an indicator that we should consider whether it was appropriate to present a pooled result. In this case, we planned to decide whether pooling was appropriate based upon the directions of the contributing effects (e.g. where all studies showed a benefit of an intervention, it could still be deemed appropriate to present a pooled estimate despite differing magnitudes of effect across studies).

We planned to use subgroup analyses to investigate the following variables as potential moderators of effects:

- Vaping/smoking history. We expected that some studies may have been carried out in people who had never smoked and some in people who had used vapes to reduce or stop smoking.
- Frequency of vaping. We expected interventions may operate differently based on the levels of vaping at baseline.
- Age. Some interventions may specifically be aimed at young people, and there are specific concerns around vaping in young people who have never smoked. It may be that interventions in young people target different elements of behaviour than those in adults, and we planned to test whether intervention effects differed in younger people compared to adults.
- Relevant intervention characteristics, such as the intensity, provider, or modality of behavioural interventions, or the dose, duration, or timing of pharmacological interventions.
- Interventions conducted in specific groups, e.g. based on level of nicotine addiction.

However, due to the small number of studies in all meta‐analyses, subgrouping was only possible by participant age category for our text message‐based intervention versus minimal support comparison for the vaping cessation at six months or longer outcome. We assessed the significance of subgroup differences based on whether the effects of subgroups would lead to differing clinical interpretations and using the I^2^ statistic (interpreted according to the thresholds discussed earlier in this section). We will seek to conduct further subgroup analyses specified above in further updates of the review where possible.

None of our analyses included enough studies to make meta‐regression possible. However, we may use meta‐regression to investigate the following variables as moderators of our aggregate outcomes in future updates of this review:

- Average age of participants in the study
- Length of time vaping at baseline (as reported by study authors)

We extracted any reports of analyses of associations between outcomes and our moderators of interest. We synthesised these narratively using effect direction plots [33].

##### Equity‐related assessment

We did not plan to investigate health inequity in this review, beyond the investigations specified above.

#### Sensitivity analysis

Where possible, we carried out sensitivity analyses for all meta‐analyses by removing studies:

- judged to be at overall high risk of bias;
- funded by the manufacturer/provider of the intervention.

We judged effects sensitive to these exclusions if the resulting effect led to a different clinical interpretation than the original effect.

We had also planned to carry out sensitivity analyses removing studies where not all participants vaped nicotine (where we were unable to separate out those people who only vaped nicotine); however, we did not include any studies that explicitly stated that participants were vaping non‐nicotine liquids and so this was not relevant. We will conduct this analysis in future updates, as appropriate.

#### Certainty of the evidence assessment

We (NL, JHB, ARB) carried out GRADE assessments and created summary of findings tables for our critical outcomes (i.e. vaping cessation at six months follow‐up or longer; change in combustible tobacco use between baseline and six months follow‐up or longer; number of people reporting SAEs at one week follow‐up or longer) using GRADEpro GDT software [34].

We generated a summary of findings table for each of the following comparisons:

- combination NRT versus control;
- cytisine versus placebo;
- varenicline versus control;
- nicotine concentration and vaping reduction versus minimal support;
- text message‐based interventions versus no/minimal support for young people.

Following standard Cochrane methodology, we used the five GRADE considerations (study limitations, consistency of effect, imprecision, indirectness, and publication bias) to assess the certainty of the body of evidence for each outcome, and to draw conclusions about the certainty of the evidence within the text of the review. Any disagreements were resolved through discussion with a third review author.

### Consumer involvement

We held a consumer planning consultation in June 2023. At this workshop, participants concluded that it would be clearer to use the term 'vape' rather than 'e‐cigarette' in the review title. We amended the title in response to this feedback. We held a second workshop and online consultation between October and December 2024 to discuss a dissemination plan for the results of this review. We will hold a further consumer consultation in 2025 to discuss future planning for this project. This will incorporate an evaluation of the living systematic review approach, dissemination used so far, and suggestions for improvements and new ways of working. This will allow us to assess whether it is appropriate and useful to continue the review. We will run a survey disseminated on public‐facing forums, such as Gumtree, Nextdoor, and X to gain the input of people who may not volunteer to be part of a more formal panel or attend a workshop in person (consumer input has indicated that different groups may be comfortable with different levels of involvement, and we want to be as inclusive as possible).

Our consumer panel have diverse vaping and smoking experiences and are from differing social backgrounds. All consumers are reimbursed for their time. We have a lead consumer contributor (CJ) who has experience of smoking combustible cigarettes and using vapes. Through phone, email, online, and in‐person project meetings, CJ is contributing to the proposed work, meeting with the chair to discuss meeting agendas beforehand, and to debrief afterwards.

We are using Cancer Research UK’s consumer toolkit and Cochrane consumer resources to assist our consumer involvement.

## Results

### Description of studies

#### Results of the search

For this new review, our bibliographic database searches identified 2470 deduplicated records (See Figure 1). We screened all records and retrieved the full‐text papers of 106 potentially relevant articles. After screening and checking the full texts, we included 51 records, representing nine new studies (Caponnetto 2023 [35, 36, 37, 38, 39]; Fucito 2024 [40, 41, 42]; Graham 2021 [43, 44, 45, 46, 47]; Klein 2024; NCT04602494 [48]; NCT04919590 [49]; Palmer 2023 [50]; Rigotti 2024 [51, 52, 53]; Sahr 2021 [54]), 20 ongoing studies (see characteristics of ongoing studies in Supplementary material 4), and 22 articles linked to the included studies. These secondary study reports are linked to the included studies in the references section of this review.

**1:**
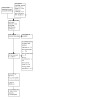


#### Included studies

Key features of the nine included studies are summarised below and in Table 6. Further details on each included study can be found in the 'Characteristics of included studies' table (Supplementary material 2).

**1:** Overview of included randomised controlled trials (all recruited participants vaping nicotine and motivated to quit vaping)

Study ID	**Number randomised**	**Study arms**	**Length of follow‐up (months)**	**Overall RoB judgement**	**Age of participants (years)**	**Participants' baseline 1) tobacco smoking; 2) vaping behaviour**	**Funded by manufacturer or provider of intervention**	**Country**	**Outcomes reported**
Caponnetto 2023	140	1) Varenicline 2) Placebo tablet	6	Low	18 and over	1) Not smoking 2) Daily vaping	Yes	Italy	Vaping cessation at 6 months; vaping cessation at between 3 and 6 months; AEs; SAEs; weight; blood pressure; heart rate
Fucito 2024	40	1) Varenicline 2) Placebo	3	Unclear	18 and over	1) Not smoking 2) Daily vaping	No	USA	Vaping cessation at between 3 and 6 months; SAEs; AEs
Graham 2021	2588	1) Text message (*This is Quitting*) 2) Control (assessment only)	7	Low	18 to 24	1) Smoking or not smoking 2) Vaping in past 30 days	Yes	USA	Vaping cessation at 6 months
Klein 2024	508	1) NRT vs text message 2) NRT + text message 3) Control	3	High	18 to 24	1) Not smoking 2) Regular vaping	No	USA	Vaping cessation at between 3 and 6 months; SAEs; AEs
NCT04602494	4	1) Varenicline 2) Placebo 3) Monitoring only	3 (planned to 6 but abandoned due to dropout)	High	18 to 24	1) Not smoking 2) Daily or near daily vaping	No	USA	Vaping cessation at 3 months (had planned at 3 to 6 months, abandoned due to dropout); AEs; SAEs
NCT04919590	1715	1) Text message (*This is Quitting*) 2) Control (assessment only) 3) Waitlist	7	Unclear	13 to 17	2) Unclear smoking status 2) Vaping in past 30 days	Yes	USA	Vaping cessation at 6 months
Palmer 2023	30	1) NRT 2) Control (quitline referral)	2	High	18 and over	1) Smoking or not smoking 2) Daily vaping	No	USA	SAEs; AEs
Rigotti 2024	160	1) Cytisinicline 2) Placebo	4	Low	18 and over	1) Not smoking 2) Daily vaping	Yes	USA	Vaping cessation at between 3 and 6 months; tobacco use at FU; SAEs; AEs; blood pressure; heart rate; cotinine
Sahr 2021	24	1) NRT + behav 2) Vaper‐taper + behav 3) Self‐taper	6	Unclear	18 and over	1) Not smoking 2) Vaping at least 4 days/week	No	USA	Vaping cessation at 6 months; vaping cessation at between 3 and 6 months; weight; blood pressure; heart rate

AE: adverse event FU: follow‐up NRT: nicotine replacement therapy RoB: risk of bias SAE: serious adverse event

##### Study types

All nine included studies were RCTs. One study, Klein 2024, employed a randomised 2 x 2 factorial study design.

##### Participants

The nine included studies represented 5209 participants. Eight studies were conducted in the USA and one in Italy. All studies were conducted in people who were currently using nicotine vapes and were interested or motivated to stop using them. Six studies were carried out among participants not using tobacco cigarettes at baseline; in a further two studies, some of the participants were dual users of vapes and tobacco cigarettes, and in one study, tobacco cigarette use at baseline was unclear. Eight studies were conducted in participants over the age of 18; three of these were exclusively in young adults aged 18 to 24. One study was conducted in 13‐ to 17‐year‐olds (NCT04919590).

##### Interventions and comparators

Included studies investigated the role of the following pharmacological interventions: varenicline, cytisinicline (also known as cytisine and hereafter referred to as such), and combination NRT. The behavioural interventions tested included text message‐based interventions and an intervention focused on reducing the nicotine content of, and time spent using, vapes.

Three studies tested varenicline versus placebo. In two of these studies, varenicline or placebo was provided for three months and follow‐up was at six months (Caponnetto 2023; NCT04602494). In the third study, varenicline was provided for eight weeks and follow‐up was at 12 weeks (Fucito 2024). One study compared cytisine to placebo tablets for 12 weeks with a 16‐week follow‐up period (Rigotti 2024). Palmer 2023 followed participants for two months and compared four weeks of combination NRT to referral to a quitline (control). A three‐month study recruiting 18‐ to 24‐year‐olds compared four study arms: 1) combination NRT + coaching calls; 2) text message‐based intervention + coaching calls; 3) combination NRT + text message‐based intervention + coaching calls; 4) coaching calls alone (Klein 2024). The NRT was supplied for eight weeks. Graham 2021 assessed a once‐a‐day text message‐based intervention (*'This is Quitting'*) for vaping cessation among young adults (18 to 24) compared to an assessment‐only control over seven months. A second study also investigated the *'This is Quitting'* text message‐based intervention compared to assessment‐only control over seven months in 13‐ to 17‐year‐olds (NCT04919590). Sahr 2021 looked at the effect of two vaping cessation methods over six months of follow‐up: 1) reducing both nicotine concentration and time spent vaping over 12 weeks and 2) 12 weeks of combination NRT. This was explored in a three‐armed study where the two interventions were compared to a minimal support control arm.

Overall, four studies followed up participants for six months or longer, four for between three and less than six months (NCT04602494 planned to follow up participants for six months but was terminated early), and one for two months.

Further details on the intervention and comparator groups for each study can be found in the 'Characteristics of included studies' table in Supplementary material 2.

##### Outcomes

Of the critical outcomes:

- Four studies reported data on vaping cessation at six months or longer (Caponnetto 2023; Graham 2021; NCT04919590; Sahr 2021).
- No studies reported data on change in combustible tobacco use between baseline and six months or longer.
- Six studies reported data on the number of participants reporting SAEs at one week or longer (Caponnetto 2023; Fucito 2024; Klein 2024; NCT04602494; Palmer 2023; Rigotti 2024).

Of the important outcomes:

- Six studies reported data on vaping cessation at three or more but less than six months from the start of the intervention (Caponnetto 2023; Fucito 2024; Klein 2024; NCT04602494; Rigotti 2024; Sahr 2021).
- One study, Rigotti 2024, reported on change in combustible tobacco product use between baseline and three or more, but less than six months from the start of the intervention.
- Five studies reported on the number of participants reporting adverse events at one week or longer in both arms (Caponnetto 2023; Fucito 2024; Klein 2024; NCT04602494; Rigotti 2024). One further study, Palmer 2023, reported AEs for the intervention arm only.
- No studies reported on the number of people vaping a substance other than nicotine at longest follow‐up, at three months follow‐up or longer.
- Two studies reported weight at follow‐up (Caponnetto 2023; Sahr 2021).
- No studies reported alcohol use status. One study, NCT04602494, stated that they would report on substances other than nicotine consumed (including tobacco and alcohol use); however, this was not reported as there were issues with recruitment and follow‐up.
- No studies reported carbon monoxide (CO), measured through breath or blood; blood oxygen saturation; lung function measures or known toxins/carcinogens.
- Three studies reported blood pressure (Caponnetto 2023; Rigotti 2024; Sahr 2021), three studies reported heart rate (Caponnetto 2023; Rigotti 2024; Sahr 2021), and one study reported cotinine (Rigotti 2024).

##### Funding

Of the nine included studies, four were funded by the manufacturer or provider of the intervention (Caponnetto 2023; Graham 2021; NCT04919590; Rigotti 2024).

Caponnetto 2023 was a trial of varenicline versus placebo, funded by GRAND (Global Research Award for Nicotine Dependence), an independently reviewed competitive grants programme funded by Pfizer Inc (USA). Pfizer is the manufacturer of Chantix/Champix (brand names for varenicline). This study was also funded by ECLAT Srl., which provides consultancy and develops, produces, and markets services and products in the field of combustion‐free devices as an alternative to traditional cigarettes. Rigotti 2024 studied cytisine versus placebo and was funded by Achieve Life Sciences, who are co‐developing a cytisine product called cytisinicline. Both Graham 2021 and NCT04919590 investigated a text message programme (*'This is Quitting'*) provided by the Truth Initiative, who also funded the studies.

#### Excluded studies

We excluded 55 studies at the full‐text screening stage. Reasons for exclusion are provided in the PRISMA diagram (Figure 1). The most common reason for exclusion was that studies did not include outcomes relevant to this review. The 16 studies that appeared to potentially meet the inclusion criteria, but which were ultimately excluded, are included in the 'Characteristics of excluded studies' table in Supplementary material 3, with reasons for exclusion.

### Risk of bias in included studies

We judged three studies to be at low risk of bias (Caponnetto 2023; Graham 2021; Rigotti 2024), three at unclear risk (Fucito 2024; Klein 2024; NCT04919590) and three at high risk (NCT04602494; Palmer 2023; Sahr 2021).

The details of the risk of bias judgements for each domain for each included study can be found in the 'Characteristics of included studies' in Supplementary material 2. Judgements across the included studies are shown in Figure 2 and for individual included studies in Figure 3.

**2:**
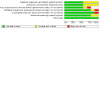
Risk of bias judgements for the included studies

**3:**
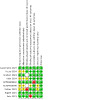
Risk of bias judgements for each domain of the included studies

#### Allocation

We judged five studies to be at low risk of bias and four studies at unclear risk of bias for random sequence generation and allocation concealment. The studies judged to be at low risk of bias for random sequence generation used methods that were considered truly random, such as a random number generator or computer‐based randomisation systems, and reported this in full. In the studies judged to be at low risk of allocation bias, concealment methods were used that meant that participants and study staff were unaware of the group to which the participants would be assigned. Where ratings were unclear, this was because reports did not provide enough information to make a judgement.

#### Blinding

We judged five studies to be at low risk of performance bias. Matched placebos were used in four studies. As per protocol, we did not assess blinding of participants and personnel (performance bias) for studies solely investigating behavioural interventions, as it is impossible to blind these types of interventions. Therefore, two studies were not judged for this domain (Graham 2021; NCT04919590). We judged one study to be at unclear risk of bias as there was not enough information to make a judgement. We judged one study to be at high risk of bias as the study arms received support of notably different intensities (Palmer 2023).

For detection bias, we judged seven studies to be at low risk, one at unclear risk, and one at high risk. We deemed Sahr 2021 high risk as vaping cessation was not biochemically validated and there was differential face‐to‐face contact between study arms.

#### Incomplete outcome data

We judged eight studies to be at low risk of attrition bias. We judged one study to be at high risk as all participants in the control group were lost to follow‐up (NCT04602494).

#### Selective reporting

We judged seven studies to be at low risk bias for selective reporting. We judged two studies to be at unclear risk. In one, the outcomes were pre‐registered; however, at the time of writing we had not been able to view the paper as it was in press (NCT04919590). The second study was not pre‐registered (Palmer 2023).

#### Other potential sources of bias

We judged all nine of the included studies to be at low risk of any other sources of bias.

### Synthesis of results

Data on our outcomes of interest are summarised by comparison below and in Table 3 (combination NRT compared to control for nicotine vaping cessation); Table 1 (cytisine compared to placebo for nicotine vaping cessation); Table 4 (varenicline compared to control for nicotine vaping cessation); Table 5 (nicotine/vaping reduction compared to minimal support for nicotine vaping cessation); and Table 2 (text message‐based interventions compared to no/minimal support for nicotine vaping cessation in young people). Analyses are presented below and are in Supplementary material 5.

#### Pharmacotherapy interventions versus controls

##### Combination NRT

###### Critical outcomes

The point estimate for one study (Sahr 2021), rated at high risk of bias, indicated higher nicotine vaping cessation rates at six‐month follow‐up in people randomised to receive combination NRT versus referral to a smoking quitline. However, the study was very small and so results were imprecise, with 95% CIs incorporating the possibility of no difference between arms or higher quit rates in the referral arm (RR 2.57, 95% CI 0.29 to 22.93; 16 participants; very low‐certainty evidence; Analysis 1.1).

Another single study (Klein 2024; N = 508), rated at unclear risk of bias, reported no SAEs in either the combination NRT + behavioural support study arms or the control study arms (with behavioural support matched to their respective intervention arms). Therefore, it was not possible to calculate a relative effect. We deemed this evidence to be of low certainty (Analysis 1.2).

None of the included studies comparing combination NRT with no/minimal support reported change in combustible tobacco use at six‐month follow‐up or longer.

###### Important outcomes

Two studies reported nicotine vaping cessation rates between three and six months follow‐up (Klein 2024; Sahr 2021). When pooled, the effect estimate indicated higher rates of quitting in the no/minimal support groups; however, the 95% CI also encompassed the potential for no effect and a benefit of combination NRT (RR 0.93, 95% CI 0.45 to 1.93; N = 524; Analysis 1.3). As well as imprecision, this analysis was limited by moderate statistical heterogeneity (I^2^ = 62%). When the one study at high risk of bias was removed, the remaining study showed evidence of greater quit rates in the combination NRT arm, but with the 95% CI also incorporating no difference (RR 1.21, 95% CI 0.97 to 1.51; N = 196).

Klein 2024 (unclear risk of bias) was the only study that reported non‐serious AEs and found evidence of more participants reporting AEs in the two study arms receiving combination NRT than the two control arms (RR 2.74, 95% CI 1.79 to 4.17; N = 379; Analysis 1.4).

Sahr 2021 (N = 16; high risk of bias) also reported weight (lbs), systolic blood pressure (mmHg), and heart rate (bpm). In all cases, results were imprecise with 95% CI incorporating potential increases, decreases, and no change in the combination NRT arm versus the minimal support arm. In the case of weight and systolic blood pressure, the point estimates indicated higher figures in the combination NRT arm at follow‐up (MD 33.93 lbs, 95% CI ‐17.57 to 85.41; N = 11; Analysis 1.5; MD 8.32 mmHg, 95% CI ‐3.98 to 20.62; N = 14; Analysis 1.6), whereas in the case of heart rate, lower bpm at follow‐up was reported in the combination NRT arm (‐4.23 bpm, 95% CI ‐24.29 to 15.83; N = 14; Analysis 1.7).

None of our other important outcomes were reported in the studies eligible for this comparison.

##### Cytisine

###### Critical outcomes

Only one study, judged to be at low risk of bias and funded by the intervention manufacturer, contributed to our cytisine versus control comparison (Rigotti 2024). This study reported one of our critical outcomes: number of participants reporting SAEs. Of the 159 participants in the trial, none reported SAEs. Therefore, it was not possible to calculate a relative effect, and we deemed the evidence to be of low certainty due to imprecision.

Nicotine vaping cessation and change in combustible tobacco use at six‐month follow‐up or longer were not measured.

###### Important outcomes

Rigotti 2024 measured nicotine vaping cessation at four months follow‐up and found that more people stopped vaping in the cytisine group than in the placebo control group; however, the 95% CI incorporated the null and a potential benefit of placebo (RR 1.77, 95% CI 0.82 to 3.82; N = 160; low risk of bias). This was the only study that reported change in combustible tobacco use; no participants were smoking at study baseline and their use of combustible tobacco was monitored to four‐month follow‐up. More participants were smoking combustible tobacco at follow‐up in the cytisine arm; however, the 95% CI was wide and incorporated both the null and potentially higher smoking rates in the placebo arm (RR 0.66, 95% CI 0.24 to 1.81; N = 160; Analysis 2.3). Eleven of the 14 participants who reported smoking across study arms (78.6%) had previously smoked; this was not broken down by study arm.

There was no clear evidence that the number of participants reporting AEs differed between arms in Rigotti 2024, with the 95% CI incorporating benefit, harm, and no effect (RR 0.93, 95% CI 0.68 to 1.27; N =159; Analysis 2.4).

Rigotti 2024 also reported mean change in systolic and diastolic blood pressure (mmHg), mean change in heart rate (bpm), and cotinine (ng/mL; a metabolite of nicotine) levels at follow‐up. For all of these outcomes, 95% CIs were wide and showed no clear effect of intervention or control. However, for change in systolic blood pressure (MD 0.90, 95% CI ‐3.35 to 5.15; N = 130; Analysis 2.5) and heart rate (MD 0.60, 95% CI ‐3.84 to 5.04; N = 130; Analysis 2.7) the effect estimate suggested lower, more favourable values in the placebo arm, whereas for change in diastolic blood pressure (MD ‐2.50, 95% CI ‐5.72 to 0.72; N = 130; Analysis 2.6) and cotinine values at follow‐up (MD ‐29.95, 95% CI ‐104.05 to 44.15; N = 126; Analysis 2.8) the effect estimate suggested lower, more favourable values in the cytisine arm.

No further important outcomes were reported by the relevant studies.

##### Varenicline

###### Critical outcomes

One study, judged at low risk of bias, eligible for the varenicline versus control (no/minimal support or placebo) comparison measured vaping cessation at six months or longer (Caponnetto 2023). This study reported evidence that more participants quit nicotine vaping in the varenicline arm than in the placebo comparator arm (RR 2.00, 95% CI 1.09 to 3.68; N = 140; Analysis 3.1). We deemed the evidence to be low certainty due to a low number of events across study arms (n = 36). Caponnetto 2023 was funded by the manufacturer of the intervention.

Three studies reported the number of participants reporting SAEs (Caponnetto 2023; Fucito 2024; NCT04602494). Two of these studies (one judged at high risk of bias and one at unclear risk of bias) reported no SAEs in either the varenicline or control arms (Fucito 2024; NCT04602494), and the third (Caponnetto 2023; low risk of bias) only reported one SAE in the varenicline arm. Therefore, the effect estimate was only calculated from the latter study, which included 95 participants for this outcome (RR 2.60, 95% CI 0.11 to 62.16; Analysis 3.2; low‐certainty evidence). As Caponnetto 2023 was funded by the intervention manufacturer, we planned to exclude this study in a sensitivity analysis; however, neither of the remaining studies reported SAEs in either arm, hence an effect estimate could not be calculated.

None of the studies included in this comparison reported change in combustible tobacco use at six‐month follow‐up or longer.

###### Important outcomes

Three studies reported vaping cessation at between three and six months follow‐up (Caponnetto 2023; Fucito 2024; NCT04602494). Pooling these three studies (N = 182) resulted in an RR of 1.64, indicating higher quit rates in the varenicline groups; however, the 95% CI (0.98 to 2.74; Analysis 3.3) did incorporate the null, and thus the potential of no difference in quit rates between varenicline and control. There was minimal statistical heterogeneity (I^2^ = 11%); however, when the one study judged to be at high risk of bias was removed (NCT04602494), the effect estimate suggested a clearer benefit of varenicline (RR 1.78, 95% CI 1.12 to 2.82). Removing the one study funded by the intervention manufacturer (Caponnetto 2023) resulted in an RR of 1.13 and 95% CI of 0.47 to 2.72, increasing the uncertainty in the possibly positive effect. However, this result should be treated with caution due to the small number of studies and the overlap in the CI resulting from the main analysis and the sensitivity analysis.

The same three studies reported the number of participants reporting AEs. The pooled estimate was imprecise with the 95% CI incorporating potential harm, benefit, and no effect of varenicline (RR 1.19, 95% CI 0.84 to 1.68; N = 130; I^2^ = 16%; Analysis 3.4). Removing the one study judged to be at high risk of bias and the one study funded by the intervention manufacturer in separate sensitivity analyses did not change the interpretation of the results.

Caponnetto 2023 alone reported weight (lbs), systolic and diastolic blood pressure (mmHg), and heart rate (bpm) at six months follow‐up. For all of these outcomes, the 95% CIs were wide and incorporated potential increases, decreases, and no difference in the varenicline arm versus the placebo arm. However, the point estimates for weight (MD ‐3.30 lbs, 95% CI ‐16.00 to 9.40; N = 95; Analysis 3.5), systolic blood pressure (MD ‐1.60 mmHg, 95% CI ‐4.93 to 1.73; N = 95; Analysis 3.6), and heart rate (MD ‐2.2 bpm, 95% CI ‐6.6 to 2.1; N = 95; Analysis 3.8) indicated lower values in the varenicline group and the point estimate for diastolic blood pressure indicated higher values in the varenicline group (MD 0.80 mmHg, 95% CI ‐2.68 to 4.28; N = 95; Analysis 3.7).

None of our other important outcomes were reported.

#### Behavioural interventions versus minimal behavioural support

##### Nicotine/vaping reduction

###### Critical outcomes

One very small study, judged to be at high risk of bias, compared a behavioural intervention reducing the nicotine concentration in vapes and reducing vaping behaviour to minimal support (referral to a tobacco quitline) (Sahr 2021). This study reported vaping cessation at six months and found greater quit rates in those using the reduction intervention; however, the 95% CI was wide and also incorporated the possibility of no intervention effect and higher quit rates in the minimal support study arm (RR 3.38, 95% CI 0.43 to 26.30; N = 17; Analysis 4.1; very low‐certainty evidence).

The numbers of participants reporting SAEs and change in combustible tobacco use at six‐month follow‐up or longer were not reported for this comparison.

###### Important outcomes

Sahr 2021 also measured nicotine vaping cessation at three‐month follow‐up. There were marginally higher quit rates in the minimal support group; however, the 95% CI was wide again (RR 0.96, 95% CI 0.57 to 1.64; N = 17; Analysis 4.2).

The other important outcomes measured by Sahr 2021 were weight (lbs), systolic blood pressure (mm/Hg), and heart rate (bpm) at follow‐up. In all three analyses there was substantial imprecision, indicating potential increases, decreases, or no change in the intervention arm versus minimal support (weight: MD 13.12 lbs, 95% CI ‐26.99 to 53.23; N = 12; Analysis 4.3; systolic blood pressure: 1.45 mmHg, 95% CI ‐10.01 to 12.91; N = 15; Analysis 4.4; heart rate: MD ‐3.80, 95% CI ‐20.22 to 12.62; N = 15; Analysis 4.5).

None of our other important outcomes were reported.

##### Text message‐based interventions

###### Critical outcomes

Graham 2021 (judged at low risk of bias) and NCT04919590 (judged at unclear risk of bias) both compared the same text message‐based nicotine vaping intervention to no/minimal support controls and reported nicotine vaping cessation at seven‐month follow‐up. The pooled analysis resulted in an RR of 1.32 (95% CI 1.19 to 1.47; N = 4091; Analysis 5.1; low‐certainty evidence) with no statistical heterogeneity detected (I^2^ = 0%). Therefore, there was no detectable moderating effect (I^2^ = 0%) of subgrouping the studies by the age of participants recruited (under 18 years versus under and over 18 years). Both of the studies included in this analysis were funded by the Truth Initiative, who provide the text message‐based intervention and both were carried out in young people, one in 13‐ to 17‐year‐olds (NCT04919590) and one in 18‐ to 24‐year‐olds (Graham 2021).

NCT04919590 also reported the number of participants reporting SAEs; zero SAEs were reported in both study arms (N = 508) and therefore it was not possible to calculate a pooled effect (Analysis 5.2; low‐certainty evidence).

Change in combustible tobacco use at six‐month follow‐up or longer was not reported for this comparison.

###### Important outcomes

An additional study, judged to be at unclear risk of bias, reported nicotine vaping cessation at three months (Klein 2024). Cessation rates were slightly higher in the text message‐based intervention; however, 95% CIs incorporated the null and a potential benefit of control (RR 1.05, 95% CI 0.84 to 1.31; N = 196; Analysis 5.3). Klein 2024 also reported the number of participants reporting AEs, resulting in an RR of 1.00 and a 95% CI also incorporating a potential benefit and harm of the text message‐based intervention (95% CI 0.70 to 1.44; N = 379; Analysis 5.4).

None of our other important outcomes were reported for this comparison.

#### Combined pharmacotherapy and behavioural interventions versus no/minimal support

##### Combination NRT + print‐based self‐help materials

###### Critical outcomes

Palmer 2023, judged to be at high risk of bias, was the only study that compared combination NRT + print‐based self‐help to minimal support. Our only outcome reported by this study was the number of people reporting SAEs. None of the participants reported SAEs across both study arms, and so it was not possible to calculate an effect estimate (N = 23; Analysis 6.1). None of our other critical outcomes (nicotine vaping cessation or change in combustible tobacco use at six‐month follow‐up or longer) were reported.

###### Important outcomes

Palmer 2023 reported that 10 of 12 participants in the intervention arm (combination NRT + print‐based self‐help materials) reported AEs. However, they did not report the AE rate in the minimal support arm and so it was not possible to calculate a relative risk.

None of our other important outcomes were reported.

##### Combination NRT + text message‐based intervention

###### Critical outcomes

Klein 2024, judged to be at unclear risk of bias, was the only study eligible for this comparison. It reported the number of people reporting SAEs and found that zero participants reported SAEs in both study arms. This meant that it was not possible to calculate an effect estimate (N = 256; Analysis 7.1).

Our other critical outcomes were not reported.

###### Important outcomes

Klein 2024 also reported nicotine vaping cessation at three months follow‐up and the number of participants reporting AEs. More participants quit vaping in the combination NRT and text messaging intervention study arm; however, 95% CI also incorporated the possibility of no effect of the intervention and a benefit in the control arm (RR 1.27, 95% CI 0.93 to 1.72; N = 256; Analysis 7.2). More adverse events were reported in the combination NRT + text message‐based intervention arm than the control arm, with the 95% CI excluding the null (RR 2.56, 95% CI 1.46 to 4.47; N = 196; Analysis 7.3).

None of our other important outcomes were reported.

#### Head‐to‐head comparisons

##### Combination NRT versus nicotine/vaping reduction

###### Critical outcomes

Sahr 2021 was the only eligible study to directly compare combination NRT to an intervention combining a reduction in both nicotine concentration and vaping frequency. This was a small study, judged to be at high risk of bias. Nicotine vaping cessation rates were slightly higher in the nicotine/vaping reduction arm; however, there was substantial imprecision and the 95% CI incorporated both benefit, harm, and no effect of either intervention (RR 0.76, 95% CI 0.17 to 3.33; N = 15; Analysis 8.1). No further critical outcomes were reported by Sahr 2021.

###### Important outcomes

Sahr 2021 also measured nicotine vaping cessation at three‐month follow‐up. Again, cessation rates were higher in the nicotine/vaping reduction arm but the 95% CI also encompassed the null and a potential benefit of combination NRT (RR 0.57, 95% CI 0.22 to 1.47; N = 15; Analysis 8.2).

The following important outcomes were also reported by Sahr 2021: weight (lbs), systolic blood pressure (mmHg), and heart rate (bpm) at follow‐up. For all of the outcomes, the 95% CIs were wide and incorporated potential increases, decreases, and no change in the combination NRT arm versus the nicotine/vaping reduction arm (weight: MD 20.80 lbs, 95% CI ‐27.49 to 69.09; N = 11; Analysis 8.3; systolic blood pressure: MD 6.87 mmHg, 95% CI ‐8.67 to 22.41; N = 11; Analysis 8.4; heart rate: MD ‐0.43 bpm, 95% CI ‐18.96 to 18.10; N = 11; Analysis 8.5).

None of our other important outcomes were reported.

##### Combination NRT versus text message‐based intervention

###### Critical outcomes

Klein 2024, judged to be at unclear risk of bias, was the only eligible study that directly compared combination NRT with a text message‐based intervention. The only critical outcome reported was the number of participants reporting SAEs, with zero events reported across study arms. Therefore, it was not possible to calculate an effect estimate (N = 252; Analysis 9.1).

Nicotine vaping cessation and change in combustible tobacco use at six‐month follow‐up or longer were not reported.

###### Important outcomes

Klein 2024 also reported nicotine vaping cessation at three‐month follow‐up. Vaping cessation rates were higher in the combination NRT arm than the text messaging arm; however, the 95% CI also incorporated the null and a potential benefit of text messaging over combination NRT (RR 1.16, 95% CI 0.84 to 1.58; N = 252; Analysis 9.2). The only other important outcome reported by Klein 2024 was the number of people reporting AEs. There was a higher rate of AEs reported in the combination NRT arm than the text messaging arm, with the 95% CI excluding the null (RR 2.99, 95% CI 1.57 to 5.70; N = 183; Analysis 9.3).

#### Associations between moderators of interest and vaping cessation

Three studies evaluated the impact of our moderators of interest on our vaping cessation outcome (Fucito 2024; Graham 2021; Rigotti 2024) (Table 7). While Fucito 2024 found evidence that vaping cessation rates were higher in those with a history of combustible tobacco use, Rigotti 2024 found no evidence of an association between the two. Fucito 2024 considered that this might be related to the fact that participants who smoked in the past were less likely to report near constant vaping prior to study entry than those who had not smoked in the past (15/21, 71.4% versus 18/19, 94.7% respectively). Rigotti 2024 also reported no evidence of an association between age and vaping cessation. Graham 2021 reported that higher vaping intensity (vaping within 30 minutes of waking) was inversely associated with vaping cessation.

**2:** Associations between moderators of interest and vaping cessation

**Study ID**	**Study design**	**Length of follow‐up (months)**	**Study size**	**Overall RoB judgement**	**Age**	**Vaping intensity**		**History of combustible tobacco use**	
Fucito 2024	RCT	3	40	Unclear				↑ *Participants who had used tobacco cigarettes. Vaping cessation: 10/21, 47.6%*	↓ *Participants who had not used tobacco cigarettes. Vaping cessation: 5/19, 26.3%*
Graham 2021	RCT	7	2588	Low		↓ *Higher‐intensity vaping. Lower vaping cessation rates: 22.6% (text message) vs 16.4% (control); P < 0.001*	↑ *Lower‐intensity vaping. Higher vaping cessation rates: 31.4% (text message) vs 28.6% (control); P = 0.51.*		
Rigotti 2024	RCT	4	160	Low	↔ No evidence of effect on cessation in intervention group			↔ No evidence of effect on cessation in intervention group	

KEY: Effect direction as reported by authors: upwards arrow ↑ = more participants quit vaping; downwards arrow ↓ = fewer participants quit vaping. ↔ = no observed association with quitting vaping. RCT: randomised controlled trial; RoB: risk of bias *Authors' judgements* *Fucito: “Adults with tobacco smoking histories were more likely to quit vapes at 3 months than those without (10/21, 47.6% vs 5/19, 26.3%), regardless of group.”* *Graham 2021: Quitting rates at 7 months were lower in both study arms in those who reported vaping within 30 minutes of waking (text message 22.6% vs control 16.4%; P < 0.001) than among those who reported vaping 30 minutes after waking (text message 31.4% vs control 28.6%; P = 0.51).* *Rigotti 2024: In the intervention (cytisinicline and behavioural support) arm “there was no evidence that the effect differed by subgroups defined by age, sex, race, history of cigarette smoking, e‐cigarette dependence, age of vaping initiation, or e‐liquid flavor used.”*

## Discussion

### Summary of main results

This new review of interventions to aid nicotine vaping cessation includes nine RCTs identified in searches up to April 2024. These studies investigated a range of comparisons, looking at pharmacotherapies, behavioural approaches, and combinations of the two. Therefore, the number of studies contributing to each comparison was minimal, limiting the conclusions that could be drawn due to serious imprecision in most cases. Three pharmacotherapies were investigated: combination NRT, varenicline, and cytisine. Despite very limited data, there was low‐certainty evidence that varenicline may improve quit rates at long‐term follow‐up when compared to placebo. However, this finding should be treated with caution as it may change as more data become available.

There was some evidence that combination NRT may lead to more non‐serious AEs than no intervention or behavioural support alone, as would be expected from any form of pharmacotherapy. Few studies reported SAEs and those that did reported zero or one event, resulting in low‐certainty evidence. However, the pharmacotherapies tested here are licensed and considered safe when used for smoking cessation.

Two forms of behavioural support were tested in isolation: reducing nicotine concentration and vaping behaviour, and text message‐based interventions. In the case of the former, data came from one very small study and so conclusions could not be drawn. In the latter case, there was low‐certainty evidence that text message‐based interventions may result in higher quit rates than no/minimal support in youth and young adults.

Some eligible studies investigated combination NRT in conjunction with print‐based self‐help and text messaging or compared to behavioural support (nicotine/vaping reduction and text messaging); however, findings were inconclusive due to the limited data available.

### Limitations of the evidence included in the review

We judged three of the included studies to be at low risk of bias, three at unclear risk, and three at high risk. We deemed two studies to be at high risk due to issues with blinding and one due to attrition. Of the nine included studies, eight were carried out in the USA and one in Italy; therefore, results may not be generalisable outside of these populations, particularly where the regulation of vaping products may differ. The majority of the included studies were carried out in adults, with only one exclusively recruiting participants under 18 years of age. Of the eight studies carried out in adults, three of these only included young adults (aged 18 to 24 years). This is particularly relevant for our comparison of text message‐based interventions versus no/minimal support; the studies contributing to the low‐certainty evidence that text message‐based interventions may help people to stop vaping nicotine recruited only young people aged between 13 and 24 years. Therefore, it is not yet possible to conclude whether this finding extends into older age groups who may have higher levels of dependence on nicotine. The majority of studies appeared to recruit a mixture of people who had smoked combustible tobacco cigarettes and who had never smoked (although this was not always stated explicitly), with only one study solely recruiting people who had previously smoked and no studies explicitly stating that they only recruited people who had never smoked. This is relevant as there may be differences in the nicotine dependence levels of people with and without a history of tobacco smoking that could affect the likelihood of successfully quitting vaping. The evidence would benefit from studies clearly stating eligibility based on baseline combustible tobacco smoking status and history. In addition, other elements besides tobacco smoking may impact dependence levels, which in turn may impact intervention effects; future studies should be clear about the vaping/nicotine dependence measures and criterion used in their studies, and report whether effects differ based on these variables.

Four of the nine studies included in the review were funded by the manufacturers of the intervention, one investigating varenicline (Caponnetto 2023), one cytisine (Rigotti 2024), and two the same text message‐based intervention (Graham 2021; NCT04919590). We intended to carry out sensitivity analyses for all outcomes, removing these studies to test their potential effects on our pooled outcomes; however, due to the limited number of studies included in each analysis, these were not informative.

We conducted GRADE ratings for our comparisons of individual pharmacotherapies and behavioural interventions when compared to placebo, minimal support, or no support, for our critical outcomes: vaping cessation at six months or longer; change in combustible tobacco use at six months or longer; and number of participants reporting SAEs. When considering our vaping cessation outcome, we deemed the evidence of very low (for combination NRT and nicotine/vaping reduction) and low (for varenicline and text message‐based interventions) certainty. No studies reported vaping cessation at six months or longer for our cytisine comparison. We downgraded the evidence on text message‐based interventions twice, to low certainty, due to indirectness. This was because the intervention being tested in both of the contributing studies was the same, and the intervention was only tested in young people. Therefore, we do not know if the same finding would be generalisable to other text message‐based apps and to older adults. Where the evidence was judged to be of very low certainty, this was because the only study contributing to a comparison was judged to be at high risk of bias, plus there was serious imprecision. Where the evidence was judged to be of low certainty, this was because there was serious imprecision alone. For all comparisons where our SAE outcome was measured by at least one study, we deemed the evidence to be of low certainty through downgrading by two levels due to imprecision. This was because few studies contributed to these analyses. The number of events was very low and where 95% CI could be calculated, these encompassed the possibility of a benefit, harm, and no effect of the intervention.

None of the studies contributing to our primary comparisons reported change in the use of combustible tobacco use at six‐month follow‐up or longer. One study reported combustible tobacco use at four‐month follow‐up; however, this was inconclusive. It is important that future studies measure this outcome, as encouraging people who have formerly smoked tobacco to stop vaping could lead to relapse to combustible tobacco smoking. Regardless of whether an intervention increases vaping quit rates, if it leads to greater smoking this would be a concern, as although vapes are unlikely to be risk‐free, evidence suggests they are considerably less harmful than combustible tobacco [22].

As the analyses conducted in this review contained small numbers of studies, we were unable to statistically assess publication bias. We made every effort to identify eligible unpublished studies, and we assessed the possibility of selective reporting for each study. We did not find any evidence of missing data relevant to the comparisons and outcomes in this review.

Overall, the evidence on interventions for quitting vaping demonstrates considerable uncertainty and more studies are needed to draw and strengthen conclusions. This review identified 20 ongoing studies that may be eligible for inclusion in this review upon completion. As such, this review will continue to be updated as a living review, with monthly searches and updates triggered as findings become available that could change our conclusions (see Supplementary material 7 for more information).

### Limitations of the review processes

We consider the methods used to carry out this systematic review to be robust. Our search strategy included a key topic‐specific conference abstract book and trial registries searched through CENTRAL, meaning we were able to capture a number of ongoing studies. However, it is possible that there are unpublished data our searches did not uncover.

For outcome assessment, we followed standard methods used for Cochrane Tobacco Addiction Group smoking cessation reviews and extended them to our vaping cessation outcomes. It is standard practice in smoking cessation research to consider participants lost to follow‐up as continuing to smoke. In this case, we assumed that participants vaping at baseline continued to vape if they were lost to follow‐up.

One of our review authors is an author of one of the included studies (NAR). This author was not involved in the decision about inclusion of their study, the risk of bias assessment for their study, or the GRADE assessments for outcomes that included their study. This approach is standard across all Cochrane reviews (regardless of subject area) and has been approved by the Cochrane editorial office as sufficient to avoid bias.

### Agreements and disagreements with other studies or reviews

A systematic review published in 2022 (with searches updated to September 2021) sought to evaluate the evidence related to the outcome of vaping cessation.[21]. Seven of the studies identified investigated the outcome of an intervention for helping a person or people to quit vaping. Two of the studies were RCTs that were also included in this review (Graham 2021; Sahr 2021), one was a single‐arm intervention study, three were case studies, and another was a case series. The interventions tested were NRT, varenicline, a text message‐based intervention, nicotine/vaping reduction, and financial incentives. All of these interventions, except financial incentives, were included in this review. The authors concluded that due to a paucity of evidence (only one study, also included in this review, was adequately powered) there was little to no evidence for effective vaping cessation strategies. This was also concluded by a more recent review of the vaping cessation evidence conducted as part of a Royal College of Physicians' report looking at e‐cigarettes and harm reduction, published in 2024 [22]. The evidence review, carried out in 2023, found that there was little evidence on the best ways to support people to stop vaping, and that further studies were required.

The pharmacological treatments investigated in this review are those with the strongest evidence for smoking cessation, i.e. cytisine, varenicline, and combination NRT [55]. This review has provided low‐certainty evidence that varenicline is also effective for quitting vaping, when compared to placebo. Clear evidence of a benefit of cytisine and combination NRT has not been demonstrated; however, this is likely (at least in part) to be due to a paucity of evidence, and in the case of all three pharmacotherapies, the incorporation of further data may change our conclusions.

Financial incentives could be tested as an intervention for vaping cessation in future trials. A Cochrane review of behavioural interventions for smoking cessation found high‐certainty evidence of the effectiveness of financial incentives for smoking cessation [56]. There was also high‐certainty evidence and moderate‐certainty evidence that counselling and text message‐based interventions were effective, respectively [56]. This review found moderate‐certainty evidence that text message‐based interventions may be effective for vaping cessation in young adults.

A Cochrane review was published in 2023, investigating interventions to prevent or cease electronic cigarette (vape) use in children and adolescents [57]. It did not find any eligible studies up to their search date of May 2023, although 22 ongoing studies were identified.

## Authors' conclusions

### Implications for practice

- There is low‐certainty evidence (downgraded two levels due to indirectness) that a text message‐based intervention may increase nicotine vaping quit rates in youth and young adults (13 to 24 years old), in comparison to no or minimal support, seven months after intervention start.
- There is low‐certainty evidence (limited by imprecision) that varenicline may increase nicotine vaping quit rates versus placebo; however, further data may change this conclusion.
- Risk of bias and imprecision preclude conclusions regarding the effects of combination nicotine replacement therapy (NRT), cytisine, and a nicotine concentration and vaping behaviour reduction programme on nicotine vaping quit rates.
- There is very limited evidence looking at serious unintended consequences of pharmacotherapies or behavioural interventions for quitting nicotine vaping, making it difficult to draw conclusions on potential harms. Where these were measured, rates of SAEs were extremely low across arms. The pharmacological interventions tested (combination NRT, cytisine, and varenicline) are licensed for the purposes of quitting smoking globally and considered safe for that indication.
- There is very limited evidence on the effectiveness and potential harms of interventions combining behavioural support and pharmacotherapies for vaping cessation and comparing relevant interventions head‐to‐head.
- None of the included studies reported whether nicotine vaping cessation interventions had an effect on the number of people smoking combustible tobacco cigarettes at six months or longer, and results of the one study measuring this at four months were inconclusive.

### Implications for research

Further randomised controlled trials (RCTs) are needed investigating interventions to help people to stop vaping, with a follow‐up period of at least six months and as long as is feasible. The interventions tested so far reflect interventions that have been found to be effective for tobacco smoking cessation. Further studies should continue to investigate these approaches and potential others, including financial incentives and counselling, which are also deemed effective for smoking cessation. It would also be helpful if studies were conducted with a comparator arm where vaping cessation was not encouraged (i.e. no treatment provided) in order to assess the effect of providing vaping cessation interventions on people's tobacco smoking rates. As well as measuring rates of vaping cessation, studies should measure unintended harms of the interventions, including serious adverse events and the impact of the interventions on rates of combustible tobacco smoking.

RCTs should ensure they are adequately powered and have processes in place to counteract risks associated with blinding and attrition (for example, using placebo as a comparator where appropriate, balancing face‐to‐face contact between study arms, biochemically validating vaping and smoking status, and optimising follow‐up contact procedures).

It is possible that the effects of interventions may differ based on the dependence levels of intervention users, which could differ according to nicotine vaping and/or tobacco smoking history and frequency of vaping. Investigators should consider the range of people to whom vaping cessation interventions are relevant, based on both tobacco smoking and vaping history, and clearly specify the baseline characteristics of participants in terms of both of these characteristics. We found low‐certainty evidence that varenicline and a text message‐based intervention may help more people quit nicotine vaping than no or minimal support. In the latter case, further research is needed to see if these findings are relevant to older adults (as well as young people), and if they extend to other text message‐based interventions.

## Supporting Information

Supplementary materials are available with the online version of this article: 10.1002/14651858.CD016058.

Supplementary materials are published alongside the article and contain additional data and information that support or enhance the article. Supplementary materials may not be subject to the same editorial scrutiny as the content of the article and Cochrane has not copyedited, typeset or proofread these materials. The material in these sections has been supplied by the author(s) for publication under a Licence for Publication and the author(s) are solely responsible for the material. Cochrane accordingly gives no representations or warranties of any kind in relation to, and accepts no liability for any reliance on or use of, such material.

**Supplementary material 1** Search strategies

**Supplementary material 2** Characteristics of included studies

**Supplementary material 3** Characteristics of excluded studies

**Supplementary material 4** Characteristics of ongoing studies

**Supplementary material 5** Analyses

**Supplementary material 6** Data package

**Supplementary material 7** Justification and methods for 'Living Review' approach

**Supplementary material 8** Data to be extracted from included studies.

## Additional information

### Registration and protocol

Protocol DOI: 10.1002/14651858.CD016058. Cochrane approved the proposal for this review in April 2024.

### Data, code and other materials

As part of the published Cochrane review, the following are made available for users of the Cochrane Library: our search strategy Supplementary material 1; full citations for all included studies, all ongoing studies, and relevant excluded studies in the reference section of the review; study data, including study information, study arms, and risk of bias assessments in our characteristics of studies tables (Supplementary material 2; Supplementary material 3; Supplementary material 4); analysis data, including overall estimates, subgroup estimates, and individual data rows (all the rows in all the analyses), which are in the main review and in Supplementary material 6. Data supporting the results of this systematic review are from published information and are available in the review. Analyses were conducted with Cochrane’s authoring tool, RevMan, using the inbuilt computation methods; for details of the computational methods, see https://documentation.cochrane.org/revman-kb/statistical-methods-210600101.html. Data were extracted in Covidence and are available from the authors on reasonable request. The data are shared within the published review directly from RevMan.

### History

Protocol first published: Issue 5, 2024
