# Supplementary material for: Interventions for quitting vaping
Source: Cochrane Database Syst Rev. 2025 Jan 8;2025(1):CD016058. doi: 10.1002/14651858.CD016058.pub2 (PMC11706636; doi:10.1002/14651858.CD016058.pub2)
Supplement: Supplementary file 1 — Supplementary material 1 Search strategies [file CD016058-SUP-01-searchStrategy.html]

Search strategies


# Supplementary material 1 to: Interventions for quitting vaping

Butler AR, Lindson N, Livingstone-Banks J, Notley C, Turner T, Rigotti NA, Fanshawe TR, Dawkins L, Begh R, Wu AD, Brose L, Conde M, Simonavičius E, Hartmann-Boyce J
  
https://doi.org/10.1002/14651858.CD016058.pub2

The material in this section has been supplied by the author(s) for publication under a Licence for Publication and the author(s) are solely responsible for the material. Cochrane has peer reviewed this material in accordance with its editorial policies, but Cochrane has not copyedited, formatted or proofread. Cochrane accordingly gives no representations or warranties of any kind in relation to, and accepts no liability for any reliance on or use of, such material.

Back to top

# Search strategies

**Initial search strategies**

**Ovid databases (MEDLINE, Embase, PsycINFO)**

1. (e-cig\* or ecig\* or electr\* cigar\* or electronic nicotine).mp. or (vape or vapes or vaporizer or vapourizer or vaporiser or vapouriser or vaper or vapers or vaping).ti,ab. or exp Electronic Nicotine Delivery Systems/ or exp Vaping/

2. (randomized controlled trial or controlled clinical trial).pt. or randomized.ab. or placebo.ab. or clinical trials as topic.sh. or randomly.ab. or trial.ti.

3. exp animals/ not human/

4. 2 not 3

5. 1 and 4

6. exp Smoking Cessation/

7. "Tobacco-Use-Cessation"/

8. (nicotine dependence or tobacco dependence).mp.

9. exp Smoking/th

10. "Tobacco-Use-Disorder"/

11. Smoking reduction/

12. exp Pipe smoking/ or exp Tobacco smoking/ or exp Tobacco Products/

13. ((quit\* or stop\* or cessation or ceas\* or giv\* or abstain\* or abstinen\* or reduc\*) adj5 (e-cig\* or ecig\* or electr\* cigar\* or electronic nicotine or vape or vapes or vaporizer or vapourizer or vaporiser or vapouriser or vaper or vapers or vaping)).ti,ab.

14. exp Tobacco/ or exp Nicotine/

15. 6 or 7 or 8 or 9 or 10 or 11 or 12 or 13 or 14

16. 5 and 15

**CENTRAL (via CRS-Web)**

1. (e-cig\* or ecig\* or electr\* cigar\* or electronic nicotine):TI,AB,KY,MH,EMT,KW,XKY,EH,KY

2. (vape or vapes or vaporizer or vapourizer or vaporiser or vapouriser or vaper or vapers or vaping):TI,AB,KY,MH,EMT,KW,XKY,EH,KY

3. MESH DESCRIPTOR Electronic Nicotine Delivery Systems EXPLODE ALL or MESH DESCRIPTOR Vaping EXPLODE ALL

4. (Quit\* or stop\* or cessation or ceas\* or giv\* or reduc\* or abstain\* or abstinen\*):TI,AB,KY,MH,EMT,KW,XKY,EH,KY

5. MESH DESCRIPTOR Smoking Cessation EXPLODE ALL or MESH DESCRIPTOR Tobacco Use Disorder EXPLODE ALL

6. #1 OR #2 OR #3

7. #4 OR #5

8. #6 AND #7

**Ongoing search strategies**

**Ovid databases (MEDLINE, Embase, PsycINFO)**

1. Meta-Analysis as Topic/

2. (meta analy$ or metaanaly$ or (systematic adj (review$1 or overview$1))).tw.

3. Meta-Analysis/

4. 1 or 2 or 3

5. (Cochrane or embase or psychlit or psyclit or cinahl or cinhal or science citation index or bids or cancerlit).ab.

6. (reference list$ or bibliograph$ or hand-search$ or relevant journals or manual search$).ab.

7. (selection criteria or data extraction).ab.

8. Review/

9. 7 and 8

10. Comment/ or Letter/ or Editorial/

11. animal/

12. human/

13. 11 not (11 and 12)

14. 11 or 13

15. 4 or 5 or 6 or 9

16. 15 not 14

17. randomized controlled trial.pt. OR controlled clinical trial.pt. OR randomized.ab. OR placebo.ab. OR clinical trials as topic.sh. OR randomly.ab. OR trial.ti.

18. exp animals/ not human/

19. 17 not 18

20. Exp case control studies/ OR Exp cohort studies/ OR Case control.tw. OR (cohort adj (study or studies)).tw. OR Cohort analy$.tw. OR (Follow up adj (study or studies)).tw. OR (observational adj (study or studies)).tw. OR Longitudinal.tw.

21. e-cig\*.mp. OR ecig\*.mp. OR electr\* cigar\*.mp. OR electronic nicotine.mp. OR (vape or vapes or vaporizer or vapourizer or vaporiser or vapouriser or vaper or vapers or vaping).ti,ab. OR Exp Electronic Nicotine Delivery Systems/

22. 16 AND 21

23. 19 AND 21

24. 20 AND 21

25. 22 OR 23 OR 24

**CENTRAL (via CRS-Web)**

1. (e-cig\* or ecig\* or electr\* cigar\* or electronic nicotine):TI,AB,KY,MH,EMT,KW,XKY,EH,KY

2. (vape or vapes or vaporizer or vapourizer or vaporiser or vapouriser or vaper or vapers or vaping):TI,AB,KY,MH,EMT,KW,XKY,EH,KY

3. MESH DESCRIPTOR Electronic Nicotine Delivery Systems EXPLODE ALL or MESH DESCRIPTOR Vaping EXPLODE ALL

4. #1 OR #2 OR #3
