# Supplementary material for: Interventions for quitting vaping
Source: Cochrane Database Syst Rev. 2025 Jan 8;2025(1):CD016058. doi: 10.1002/14651858.CD016058.pub2 (PMC11706636; doi:10.1002/14651858.CD016058.pub2)
Supplement: Supplementary file 2 — Supplementary material 2 Characteristics of included studies [file CD016058-SUP-02-characteristicsOfIncludedStudies.html]

Characteristics of included studies


# Supplementary material 2 to: Interventions for quitting vaping

Butler AR, Lindson N, Livingstone-Banks J, Notley C, Turner T, Rigotti NA, Fanshawe TR, Dawkins L, Begh R, Wu AD, Brose L, Conde M, Simonavičius E, Hartmann-Boyce J
  
https://doi.org/10.1002/14651858.CD016058.pub2

The material in this section has been supplied by the author(s) for publication under a Licence for Publication and the author(s) are solely responsible for the material. Cochrane has reviewed this material, but Cochrane has not copyedited, formatted or proofread. Cochrane accordingly gives no representations or warranties of any kind in relation to, and accepts no liability for any reliance on or use of, such material.

Back to top

# Characteristics of included studies

## Table of contents

- Studies ordered by Study ID
  - Caponnetto 2023
  - Fucito 2024
  - Graham 2021
  - Klein 2024
  - NCT04602494
  - NCT04919590
  - Palmer 2023
  - Rigotti 2024
  - Sahr 2021
- References to studies

## Studies ordered by Study ID

Caponnetto 2023

| ***Study characteristics*** | | |
| Methods | Design: Randomized controlled trial. Parallel group Country: Italy  Setting: Centro per la Prevenzione e Cura del Tabagismo (CPCT), the University-run smoking cessation center Study aim: The aim of this double-blind randomized placebo-controlled trial was to evaluate the efficacy and safety of varenicline (1 mg BID, administered for 12 weeks, and followed to week 24) combined with vaping cessation counseling in exclusive daily EC users intending to quit vaping.  Recruitment: local vape shops, databases of people who previously smoked who attended a local smoking cessation center (Centro per la Prevenzione e Cura del Tabagismo (CPCT), University of Catania) and stopped smoking by switching to ECs, databases of people who previously smoked who took part in CoEHAR (CoEHAR, University of Catania) sponsored tobacco harm reduction and switching studies, social networks, WhatsApp chat of undergraduates and postgraduates of the University of Catania, and word of mouth among relatives and friends of study participants.  Blinding: Participants, study staff and statistical team were blinded to randomisation and treatment assignment. Blinding was ensured by the identical appearance of drug and placebo tablets and their containers.  Study length 24 weeks.  Start date: 2018. End date: 2020 | |
| Participants | 140 randomised  70 randomised to varenicline (51 at 24 week FU) 70 randomised to placebo (44 at 24 week FU)  Age group 18 and over. All exclusive daily EC users intending to quit vaping who had formerly smoked. No dual users.  Varenicline arm Mean age 53.8 (SD 9.7). 48.6% female. Main vaping device: Refillable tank 55 (78.6%); refillable pod/cartridge 9 (12.9%); closed pod/cartridge system 5 (7.1%); disposable 1 (1.4%) Main e-liquid flavour: Tobacco: n=32 (45.7%); Fruit: n=15 (21.4%); Mint: n=6 (8.6%); Dessert: n= 11 (15.7%); Mixed: n=6 (8.6%)  Placebo arm Mean age 51.3 (SD 8.4); 52.9% female. Main vaping device: Refillable tank n= 53 (75.7%); refillable pod/cartridge n= 10 (14.3%); closed pod/cartridge system n= 7 (10.0%); disposable n= 0 (0%) Main e-liquid flavour: Tobacco n= 34 (48.5%); fruit n=17 (24.3%); mint n= 4 (5.7%); dessert n=9 (12.9%); mixed n=6 (8.6%) | |
| Interventions | Arm 1. Varenicline for 12 weeks Varenicline (1.0 mg, administered twice daily for 12 weeks) (0.5 mg/day for 2–3 days, 0.5 mg twice daily for 4–5 days; then 1 mg twice daily for 11 weeks). One-on-one vaping cessation counseling was provided at each visit for a total of 10 min by clinical psychologists with experience in nicotine dependence and vaping behaviors.  Arm 2. Placebo Film coated placebo tablet twice daily, for 12 weeks.One-on-one vaping cessation counseling was provided at each visit for a total of 10 min by clinical psychologists with experience in nicotine dependence and vaping behaviors. | |
| Outcomes | Study length 24 weeks. Time points for data collection baseline, weekly to week 12, 24 weeks. Not all data collected at all time points.  Nicotine vaping cessation; Weight: (pounds); BMI; Blood pressure systolic; Blood pressure diastolic; Heart rate (bpm); Serious adverse events. Number of participants reporting any type of serious adverse event(s); Adverse events. Number of participants reporting any type of adverse event(s). | |
| Identification | Title: Varenicline and counseling for vaping cessation: a double-blind, randomized, parallel-group, placebo-controlled trial Trial ID (if provided): EUCTR2016-000339-42-IT Author's name: Pasquale Caponnetto Institution: Centre for the Prevention and Treatment of Tobacco Addiction (CPCT) Email: polosa@unict.it; p.caponnetto@unict.it Address: Centre for the Prevention and Treatment of Tobacco Addiction (CPCT), University Teaching Hospital "Policlinico-Vittorio Emanuele", University of Catania, Italy | |
| Study funding | Funding statement: "This investigator-initiated research is supported by grant WS53232647 from GRAND (Global Research Award for Nicotine Dependence), an independently reviewed competitive grants program funded by Pfizer Inc (USA). Editorial support for the editing of this manuscript was provided by ECLAT Srl. to Mike Coughlan. Support for open access is provided by Università di Catania under the CRUI-CARE Agreement. The funders had no role in the design and conduct of the study; collection, management, analysis, and interpretation of the data; preparation, review, or approval of the manuscript; and decision to submit the manuscript for publication."  Pfizer manufactures Chantrix (varenicline) | |
| Author declarations | Declarations of interest statement: "RP is a full tenured professor of Internal Medicine at the University of Catania (Italy) and Medical Director of the Institute for Internal Medicine and Clinical Immunology at the same University. He has received grants from U-BIOPRED and AIR-PROM, Integral Rheumatology & Immunology Specialists Network (IRIS), Foundation for Smoke Free World, Pfizer, GlaxoSmithKline, CV Therapeutics, NeuroSearch A/S, Sandoz, Merk Sharp & Dohme, Boehringer Ingelheim, Novartis, Arbi Group Srl., Duska Therapeutics, Forest Laboratories, Ministero dell Universita’ e della Ricerca (MUR) Bando PNRR 3277/2021 (CUP E63C22000900006) and 341/2022 (CUP E63C22002080006), funded by NextGenerationEU of the European Union (EU), and the ministerial grant PON REACT-EU 2021 GREEN- Bando 3411/2021 by Ministero dell Universita’ e (MUR) – PNRR EU Community. He is the founder of the Center for Tobacco Prevention and Treatment (CPCT) at the University of Catania and of the Center of Excellence for the Acceleration of Harm Reduction at the same university. He receives consultancy fees from Pfizer, Boehringer Ingel- heim, Duska Therapeutics, Forest Laboratories, CV Therapeutics, Sermo Inc., GRG Health, Clarivate Analytics, Guidepoint Expert Network, and GLG Group. He receives textbooks royalties from Elsevier. He is also involved in a patent application for ECLAT Srl. He is a pro bono scientific advisor for Lega Italiana Anti Fumo (LIAF) and the International Network of Nicotine Consumers Organizations (INNCO); and he is Chair of the European Technical Committee for Standardization on “Requirements and test methods for emissions of electronic cigarettes” (CEN/TC 437; WG4).PC has been affiliated to the CoEHAR since December 2019 in a pro bono role. He is co-author of a protocol paper supported by an Investigator-Initiated Study award program established by Philip Morris International in 2017. The other authors have no conflict of interest to declare. In the past three years, CR’s organization, Russell Burnett Research & Consul- tancy Ltd, has received funding from e-cigarette/tobacco product manufacturers to conduct behavioral research on population use, use intentions, and perceptions of e-cigarettes/vaping products, and on theeffects of e-cigarette use/vaping on tobacco use transitions.JSA received sponsored funds for travel expenses as a speaker for the 2021 and 2022 annual GTNF conference. JSA serves as a consultant and has equity in Qnovia, a start-up company.All other authors have no conflict of interest to declare." | |
| Notes | Trial registry was amended to split into 2 studies EC users and dual (EC + tobacco cigarette) users.  The data is from the EC users study. Not dual users | |
| ***Risk of bias*** | | |
| **Bias** | **Authors' judgement** | **Support for judgement** |
| Random sequence generation (selection bias) | Low risk | Judgement Comment: "The list for treatment randomization was generated using SAS software (SAS Institute). The size of the blocks was a variable of 5, and the sequence of blocks was randomized and blinded." |
| Allocation concealment (selection bias) | Low risk | Judgement Comment: "Participants, study staff and statistical team were blinded to randomisation and treatment assignment. Blinding was ensured by the identical appearance of drug and placebo tablets and their containers." |
| Blinding of participants and personnel (performance bias) All outcomes | Low risk | Judgement Comment: "Participants, study staff and statistical team were blinded to randomisation and treatment assignment. Identical appearance of drug and placebo tablets and their containers. Same vaping cessation counseling for both arms. Equally intense interventions." |
| Blinding of outcome assessment (detection bias) All outcomes | Low risk | Quote: "Saliva samples were collected for cotinine measurement in those who stated they had not vaped and with an  eCO≤7 ppm" "analyzed in duplicate" "cotinine cut-off for abstinence of 10 ng/ml " Judgement Comment: "BP, HR, weight etc measured. Vaping cessation: cotinine level-verified self-reported abstinence" |
| Incomplete outcome data (attrition bias) All outcomes | Low risk | Judgement Comment: "Completed 51/70 (73%) (varenicline), 44/70 (63%) (placebo). Difference in LTFU across arms <20%" |
| Selective reporting (reporting bias) | Low risk | Judgement Comment: "Outcome measures were pre-specified. CO not reported in final paper but could be due to splitting the trial between dual users and exclusive vapers." |
| Other bias | Low risk | Judgement Comment: "No other sources of bias noted." |

Fucito 2024

| ***Study characteristics*** | | |
| Methods | Design: Randomized controlled trial Parallel assignment, double blind, placebo controlled 2-site Country: USA Setting: 2 sites: Connecticut (Yale) & South Carolina (Medical University of South Carolina).  Study aim: Preliminary clinical trial of varenicline (daily titration to 2 mg, FDA-approved for smoking cessation) investigated its potential for e-cigarette cessation in adults who reported exclusive daily use for ≥6 months and no other past 30-day tobacco use.  Start date: 2022-11-04. End date: 2023-09-01 | |
| Participants | Total sample size: 40  Varenicline = 20; Placebo = 20 Completed 12 week FU: Varenicline = 18; Placebo = 15.  Age group: 18 and over. All nicotine vape users. No dual users.  Inclusion criteria: daily use of a nicotine vape (use for at least 25 days out of the past month); use of a nicotine vape for more than 6 months; desire to quit vapes; willing to set a quit date; smartphone /email.  Exclusion criteria: have smoked any combustible cigarettes in the past 6 months; individuals with medical contraindications for varenicline use (i.e., severe renal impairment); using smoking cessation medications; vulnerable populations.  Mean age 28.2 (SD 8.5). 47.5% female. Main vaping device : Disposable 50% (n=20); Closed pod/cartridge 35% (n=14); refillable pod/cartridge 10% (n=4); refillable tank 5% (n=2) Nicotine salt-based EC 90% (n=36) | |
| Interventions | 8-week treatment period and a 4-week follow-up for both groups  Arm 1. Varenicline + Self-Change Pamphlet. 8 weeks varenicline. Minimal, self-guided behavior change booklet. This booklet includes general tips for e-cigarettes cessation and information about the free web-based e-cigarette cessation program sponsored by The Truth Initiative and Mayo called "This is Quitting". A single counseling session (medication guidelines, brief cessation advice, and instructions to set a quit date 1−2 weeks after medication initiation). Weekly electronic diaries assessed EC, medication use and AEs. Days 1-3: 0.5mg study pill once per day, Days 4-7: 0.5mg study pill twice per day, Weeks 2-8: 1mg study pill twice per day. Minimal, self-guided behavior change booklet.  Arm 2. Placebo + Self-Change Pamphlet. 8 weeks placebo pill. Minimal, self-guided behavior change booklet. This booklet includes general tips for e-cigarettes cessation and information about the free web-based e-cigarette cessation program sponsored by The Truth Initiative and Mayo called "This is Quitting". A single counseling session (medication guidelines, brief cessation advice, and instructions to set a quit date 1−2 weeks after medication initiation). Weekly electronic diaries assessed EC, medication use and AEs. Days 1-3: 0.5mg placebo pill once per day. Days 4-7: 0.5mg placebo pill twice per day. Weeks 2-8: 1mg placebo pill twice per day. Minimal, self-guided behavior change booklet. | |
| Outcomes | Baseline, 8 weeks, 12 week FU  Nicotine vaping cessation; adverse events (number of participants reporting any type of adverse event(s)); serious adverse events (number of participants reporting any type of serious adverse event(s)). | |
| Identification | Lisa Fucito, Department of Psychiatry, Yale School of Medicine, lisa.fucito@yale.edu Title: Varenicline for e-cigarette cessation in adults: a preliminary placebo-controlled randomized trial Trial ID: NCT05541497 | |
| Study funding | "The research was directly supported through internal department funds from Drs. Fucito and Toll. In addition, investigators were supported during this study by NIH grants: P30CA138313 for NLB, AMP, MJC, KMG, and BAT; P30CA016359 for LMF; and K23DA045957 for SRB. It was also supported by an American Heart Association (AHA) grant, 20YVNR35460041, to SKS and SOM." | |
| Author declarations | "Unrelated to this study, authors report the following financial disclosures. Dr. Fucito has received funding from Imbrium Therapeutics, LLC for serving on an advisory board. Dr. Toll has testified on behalf of plaintiffs who have filed litigation against the tobacco industry. Dr. Gray has provided consultation to Indivior and Jazz Pharmaceuticalsand has received research support from Aelis Farma. Dr. O’Malley has been a member of the Alcohol Clinical Trials Ini-tiative (ACTIVE) Workgroup, which has been supported by Alkermes, Dicerna, Beam Diagnostics, Ethypharm, Lundbeck, Mitsubishi, and Otsuka. She has been a consultant to Dicerna, received medication supplies from Novartis/Stalicla, and is an inventor on a patent application with Yale and Novartis. Dr. Krishnan-Sarin has received free medication supplies from Novartis/Stalicla for investigations on alcohol drinking behaviors. SRB, NLB, AMP, and MJC have no financial disclosures to report." | |
| ***Risk of bias*** | | |
| **Bias** | **Authors' judgement** | **Support for judgement** |
| Random sequence generation (selection bias) | Unclear risk | Judgement Comment: "No details provided about the randomisation process" |
| Allocation concealment (selection bias) | Unclear risk | Judgement Comment: "No details provided about the method used to conceal allocation sequence " |
| Blinding of participants and personnel (performance bias) All outcomes | Low risk | Judgement Comment: "Varenicline vs placebo tablet, equal intensity." |
| Blinding of outcome assessment (detection bias) All outcomes | Low risk | Judgement Comment: "Similar support. Abstinence was self-report but the two arms received the same level of support (self-guided cessation booklet & single counselling session)." |
| Incomplete outcome data (attrition bias) All outcomes | Low risk | Judgement Comment: "33/40 Varenicline 18/20 followed up Placebo 15/20  < 20% between groups" |
| Selective reporting (reporting bias) | Low risk | Judgement Comment: "Outcome measures pre-specified accurately on clinical trials.gov" |
| Other bias | Low risk | Judgement Comment: "No other sources of bias detected." |

Graham 2021

| ***Study characteristics*** | | |
| Methods | Design: Randomized controlled trial. Parallel group Country: USA General population (young adults, 18-24) Study aim: To determine the effectiveness of a text message program for vaping cessation among young adults compared to an assessment-only control. Recruitment: Participants were recruited via social media ads. Web advertisements on various platforms (eg, Facebook and Twitter) describe the study opportunity and lead to the study website, with details about study participation, including incentives for participation. Blinding: Single (participants). Participants are not explicitly told they are in the control group, although they may deduce their assignment from presence or lack of regular text message intervention.  Start date: 2019-12-19. End date: 2020-11-12 Study length: 7 months | |
| Participants | Total sample size: 2588 randomised  Intervention (This is quitting) = 1304; Control = 1284  Number of withdrawals: Intervention: 331 lost to FU at 7 months. Control: 290 lost to FU at 7 months.  Inclusion criteria: Age 18-24 years. Current nicotine EC use in the past 30 days. Interest in quitting vaping in the next 30 days. Ownership of a mobile phone with an active text message plan US residence.  Mean age (years) ± SD: 20.4 (1.7); % female: 50.3 Alcohol use (as reported by author): Binge drinking in last 30 days 1929/2588 (74.5%) | |
| Interventions | Intervention arm: This is Quitting Description of intervention: Text message-based intervention for quit vaping support. Users receive one age-appropriate message per day tailored to their enrollment date or quit date, which can be set and reset via text message. Those not ready to quit receive 4 weeks of messages focused on building skills and confidence. Users who set a quit date receive messages for a week preceding it and 8 weeks afterward that include encouragement and support, skill- and self-efficacy building exercises, coping strategies, and information about the risks of vaping, benefits of quitting, and cutting down to quit. Keywords COPE, STRESS, SLIP, and MORE provide on-demand support. Instructions for use for intervention:  Frequency of intervention: One text per day. Plus (as for control) Text message at 14 days and then monthly. Other: All participants were compensated $5 via digital gift card per response (7 assessments total for a maximum compensation of $35). These assessments were designed solely to maximize retention; they were not analyzed as outcomes. Participants were paid $20 for completing each follow-up survey; they earned an additional $10 incentive for responding within 24hours of the initial invitation. Support for quitting combusted tobacco products was not explicitly provided in TIQ  Control. Assessment only Description of intervention: Initial enrollment message, then contacted periodically to assess e-cigarette use. At the end of the intervention period and 7 mth FU, receive information on how to sign up for This is Quitting. Both arms: Incentivized text message assessments regarding e-cigarette use and abstinence were sent to all participants at 14 days post randomization and monthly thereafter through a 6-month period.  Instructions for use for intervention:  Frequency of intervention: Text message at 14 days and then monthly.   Other: All participants were compensated $5 via digital gift card per response (7 assessments total for a maximum compensation of $35). These assessments were designed solely to maximize retention; they were not analyzed as outcomes. Participants were paid $20 for completing each follow-up survey; they earned an additional $10 incentive for responding within 24 hours of the initial invitation | |
| Outcomes | Data collection time points: baseline, 1 month, 7 months  Nicotine vaping cessation | |
| Identification | Title: Effectiveness of a vaping cessation text message program among young adult e-cigarette users a randomized clinical trial Trial ID (if provided): NCT04251273 Author's name: Amanda L Graham Institution: Innovations Center, Truth Initiative, Washington DC, USA. Dept of Medicine, Mayo Clinic College of Medicine and Science, Rochester, MN, US Dept Oncology, Georgetown University Medical Center, Washington, DC, US. Center for Statistical Sciences, Brown Uni Email: agraham@truthinitiative.org | |
| Study funding | "This study is funded by the Truth Initiative with support from the CVS Health Foundation. The funding sources had no role in the design of this study and will not have any role during its execution, analyses, interpretation of the data, or decision to submit."  This is Quitting is a text messaging program run by Truth Initiative. | |
| Author declarations | Declarations of interest statement: AG, MJ, MA, SC, and MB are employed by Truth Initiative, a nonprofit public health foundation that sells enterprise digital tobacco cessation programs. | |
| ***Risk of bias*** | | |
| **Bias** | **Authors' judgement** | **Support for judgement** |
| Random sequence generation (selection bias) | Low risk | Judgement Comment: ""Randomized to treatment or control by a computer algorithm that automated random allocation in a 1:1 sequence."" |
| Allocation concealment (selection bias) | Low risk | Judgement Comment: "From Graham 2021: "Random assignments were concealed from participants and research staff throughout the trial." "Randomized to treatment or control by a computer algorithm that automated random allocation in a 1:1 sequence."" |
| Blinding of outcome assessment (detection bias) All outcomes | Low risk | Judgement Comment: "Self reported vaping cessation but no face to face contact." |
| Incomplete outcome data (attrition bias) All outcomes | Low risk | Judgement Comment: "621/2588 24% drop out at 7 months <50% This is quitting (text messages) 331/1304 = 25.4% drop out Control 290/1284 = 22.6% drop out <20% difference between arms" |
| Selective reporting (reporting bias) | Low risk | Judgement Comment: "Vaping abstinence reported as set out in NCT record." |
| Other bias | Low risk | Judgement Comment: "No other sources of bias noted." |

Klein 2024

| ***Study characteristics*** | | |
| Methods | Design: Randomized controlled trial. Parallel group. 2x2 factorial design. Factorial Assignment.  Country: USA  18-24 year old EC users interested in quitting  Recruitment: Social media. Recruitment firm(https://climb.care/) where advertisements were placed on Instagram and Facebook;additional methods included other social media or online methods (Reddit, StudySearch,etc.). A two-step recruitment process, initial screening survey hosted by the recruitment firm. Then those who met all eligibility criteria listed above were referred to an online baseline survey.  Study aim: The aim of this study is to test intervention components to help young adults quit vaping. A 2x2 factorial design will be used where all participants receive quitline-delivered behavioral phone counseling, and components to be tested are a digital intervention (with text and online cessation support) and nicotine replacement therapy (NRT).  Blinding: Evaluators are blinded to the study arm assignment of participants. Participants received a USD 40 e-gift card following the baseline survey participation and completion of the cessation coaching call  Study start date 2021-07-05. Study completion: 2023-06-30 (estimated). Results available online. | |
| Participants | Total sample size: 508  Combination NRT + 2 phone coaching calls ['CoachingNRTArm'] n=126 [Completed 96]  Text message-based intervention + 2 phone coaching calls ['DigitalArm'])n=126 [Completed 87].  Combination NRT + text-based intervention + 2 phone coaching calls ['DigitalNRTArm'] n=122 [Completed 93]  2 phone coaching only arm (control) ['CoachingOnlyArm']n=134 [Completed = 101]  Age 18-24 (18-21 = 57.1%; Age 22-24= 42.9%); Female 71.3%; Daily vape use: 77.9%. Less than daily 22.1%; Vape use 3-+ times per day = 37.2%. Vape use less than 30 times per day = 62.8%.  Current, regular user of nicotine e-cigarettes (20+ days in the last month). Exclusive e-cigarette user (no other tobacco in the last 30 days; or no other tobacco in the last 90 days if smoked 100+ cigarettes or cigarillos in lifetime). Interest in quitting in the next 30 days. Ownership of a smartphone device. All exclusive vape users interested in quitting. Study not restricted to never-smokers. | |
| Interventions | Combination NRT. Text message-based intervention.  Arm 1. Combination NRT + 2 phone coaching calls ['CoachingNRTArm'] n=126 Description of intervention: NRT 8 week supply of nicotine patch, gum, and/or lozenge. Single or combination NRT (patch plus gum or lozenge). PLUS two proactive phone-based behavioral coaching calls for quitting vaping, (all arms receive the 2 calls). NRT discussed and dosed by the study quit coach during coaching calls per quitline dosing protocols. 8 week supply of nicotine patch, gum, and/or lozenge. NRT will be sent in two 4 week shipments. Participants may be dosed for a single form of NRT or combination NRT (patch plus gum or lozenge) based on coach assessment of nicotine use and participant preference.  Arm 2. Text message-based intervention + 2 phone coaching calls ['DigitalArm'])n=126 Text-based vaping cessation program that utilizes evidence-based content from the quitline tobacco cessation protocol, and links directing participants to additional online educational content (brief videos, audio content, quizzes, and educational activities). PLUS two proactive phone-based behavioral coaching calls for quitting vaping, (all arms receive 2 calls)  Arm 3. Combination NRT + text-based intervention + 2 phone coaching calls ['DigitalNRTArm'] n=122 NRT (8 week supply) + Text based vaping cessation program PLUS two proactive phone-based behavioral coaching calls for quitting vaping (all arms receive 2 calls). NRT dosed by coach (patch, gum, and/or lozenge).   Arm 4. 2 phone coaching only arm (control) ['CoachingOnlyArm']n=134 Participants in all four arms of the study will receive two proactive phone-based behavioral coaching calls for quitting vaping, which will include making a quit plan, learning to cope with urges to vape, and education on strategies for quitting and staying quit. Calls utilize the quitline evidence-based protocol. The participants are encouraged to call in for ad hoc calls if they would like additional support. First call 20 mins, second 10 mins. | |
| Outcomes | Baseline, 3 months  Nicotine vaping cessationAdverse events; Number of participants reporting any type of adverse event(s); Serious adverse events. Number of participants reporting any type of serious adverse event(s) | |
| Identification | Elizabeth G. Klein, Ohio State University, klein.232@osu.edu Title: Research and innovation to stop e-cigarette/​vaping in young adults ((RISE)) Official title: Young adult vaping cessation: a randomized trial examining phone coaching, text-based digital Intervention, and nicotine replacement therapy Trial ID: NCT04974580 | |
| Study funding | "This research was funded by the American Heart Association as part of the End Nicotine Addiction in Children and Teens (ENACT) program." | |
| Author declarations | "K.M.C., K.M., and K.A.V. are employees of RVO Health, the provider of quitline cessation services in the randomized trial." | |
| ***Risk of bias*** | | |
| **Bias** | **Authors' judgement** | **Support for judgement** |
| Random sequence generation (selection bias) | Unclear risk | Judgement Comment: ""Randomized to receive NRT (yes vs. no) and/or the mHealth program (yes vs. no; text message program with links to videos, podcasts, and other online learning content), delivered using a factorial design." |
| Allocation concealment (selection bias) | Unclear risk | Judgement Comment: "State: evaluators are blinded to the study arm assignment of participant. No further detail.  Apart from mentioning that participants were randomised to receive interventions using a factorial design, no more information about the allocation concealment" |
| Blinding of participants and personnel (performance bias) All outcomes | Unclear risk | Judgement Comment: "Evaluators are blinded to the study arm assignment of participants. No detail on blinding of participants. Interventions are of similar intensity. " |
| Blinding of outcome assessment (detection bias) All outcomes | Low risk | Judgement Comment: Self-reported vaping cessation (30 day point prevalence abstinence). However, behavioural support was provided remotely and so face-to-face contact was matched between arms. |
| Incomplete outcome data (attrition bias) All outcomes | Low risk | Judgement Comment: "377/508 completed <50% drop out (74.21% completed) Control (all receive 2 phone calls) 101/134 (75.4% completed) Digital 87/126 (69%) NRT 96/126 (75%) NRT + digital 93/122 (76.2%) <20% difference between arms" |
| Selective reporting (reporting bias) | Low risk | Judgement Comment: "All results of outcomes specified are reported in NCT record." |
| Other bias | Low risk | Judgement Comment: "No other sources of bias noted." |

NCT04602494

| ***Study characteristics*** | | |
| Methods | Design: Randomized controlled trial. Parallel assignment. Pilot RCT Country: USA Setting: Boston area.  Age group: 18-25  Study aim: Randomized, placebo-controlled trial to test the hypothesis that varenicline added to group behavioral and texting support will be well tolerated and improve vaping cessation rates among nicotine dependent adolescents who vape, do not smoke regularly, and are willing to try treatment to stop vaping compared to placebo added to group behavioral and texting support.  Recruitment via: 1) annual high school screenings, 2) direct physician referral from local MGH clinics and the MGB Healthcare network, 3) teacher, counselor, administrator, parent/guardian referral, 4) response to postings online, flyers, streaming services, through social media advertisements, and through traditional advertisements, 5) identification via research databases.  Blinding: Study staff and participants will both be blind to drug for those randomized to the varenicline and placebo arm.  This is the pilot study for NCT05367492  Start date: 2020-12-18. End date: 2022-06-27 .  Study length 24 weeks. Intervention to week 12. FU at 24 weeks. | |
| Participants | Total sample size: 4  Five participants signed consent and were enrolled. One (1) subject was considered a screen fail and not randomized to an arm/group, and four (4) subjects were randomized.  Varenicline=1; Placebo = 1; Monitoring only control = 2 Withdrawals: 2/4 in the monitoring only control arm did not complete study.  Mean age (years) ± SD: 21.25(0.43). % female: 100%.  Daily or near daily vaping. No tobacco cigarette use at baseline. No use of tobacco cigarettes in past 30 days ( for 2 months or more). Not restricted to never-smokers. Not restricted to current or former smokers.  Ages 18-25. All motivated to quit vaping in the next 30 days. Daily or near daily nicotine vaping for the prior ≥ 3 months. No combusted tobacco use in the past 2 months at enrollment.  Exclusion criteria: Use of a smoking cessation medication in the prior month (nicotine patch, gum, nasal spray, or inhaler, varenicline, bupropion). | |
| Interventions | Varenicline (chantix) Varenicline 0.5 mg once daily on days 1-3, 0.5 mg twice daily on days 4-7 and starting on day 8, 1 mg twice daily for a total of 12 weeks added to 12 weeks of group behavioral and texting support specifically designed for teen vaping cessation. Daily to day 3. Twice daily from day 4 Other: 12 weeks of group behavioral and texting support specifically designed for teen vaping cessation  Placebo Identical placebo 0.5 mg once daily on days 1-3, 0.5 mg twice daily on days 4-7 and starting on day 8, 1 mg twice daily for a total of 12 weeks added to 12 weeks of group behavioral and texting support specifically designed for teen vaping cessation. Daily to day 3. Twice daily from day 4 Other: 12 weeks of group behavioral and texting support specifically designed for teen vaping cessation  Monitoring only Control Frequency of intervention: Participants will attend weekly and monthly sessions that will only consist of assessments. No study medication, no behavioral or texting support. | |
| Outcomes | Baseline, weeks 4, 8, 12, 16, 20 and 24.  Nicotine vaping cessation; adverse events; serious adverse events; combustible tobacco use; Minnesota Withdrawal Scale (MNWS) 9 item self-rated scale of nicotine withdrawal symptoms; Questionnaire of Vaping Craving (QVC), a 10-item measure of vaping craving, with scores ranging from 10 - 70, where higher scores indicate greater craving for vaping products. | |
| Identification | A. Eden Evins, Massachusetts General Hospital, Boston MA, USA  Title: Varenicline for nicotine vaping cessation In non smoker vaper adolescents (pilot) NCT04602494 2020 | |
| Study funding | NIH | |
| Author declarations | No statement provided | |
| ***Risk of bias*** | | |
| **Bias** | **Authors' judgement** | **Support for judgement** |
| Random sequence generation (selection bias) | Low risk | Judgement Comment: "Randomization will be computer generated by the MGH Research Pharmacy personnel with no other interactions with study staff or participants." "Participants, investigators and outcome assessor will remain fully blind to all 3 arms" |
| Allocation concealment (selection bias) | Low risk | Judgement Comment: "The full randomization code (drug, placebo, monitoring) will be held in the MGH research pharmacy and available to study PI only in the case of urgent medical need. A partial randomization code (treatment vs monitoring) will be held by the interventionist at the Center for Addiction Medicine. Participants, investigators and outcome assessor will remain fully blind to all 3 arms" |
| Blinding of participants and personnel (performance bias) All outcomes | Low risk | Judgement Comment: "Participants, investigators and outcome assessor will remain fully blind to all 3 arms. Varenicline and placebo equally intensive. |
| Blinding of outcome assessment (detection bias) All outcomes | Low risk | Vaping cessation was biochemically validated with an appropriate method (urinary cotinine <50 ng/mL). Urine cotinine testing is valid as Varenicline does not contain nicotine. Outcome assessors are unaware of participants' group assignments." |
| Incomplete outcome data (attrition bias) All outcomes | High risk | Varenicline: 1/1 completed (100%). Placebo: 1/1 completed (100%). Monitoring Only: 0 / 2 completed (0% completion rate, 100% attrition). >20% between group difference. |
| Selective reporting (reporting bias) | Low risk | Judgement Comment: "The study has detailed and pre-specified primary and secondary outcomes, including measures for abstinence, adverse events, and various symptom scales.  Primary outcomes are comprehensively reported. The lack of data for some secondary measures is likely due to the small sample size and high attrition in the monitoring group rather than selective reporting." |
| Other bias | Low risk | Judgement Comment: "No other sources of bias detected." |

NCT04919590

| ***Study characteristics*** | | |
| Methods | Design: Randomized controlled trial. Parallel assignment RCT with waitlist control.  Country: USA  Recruitment: Online  Age: 13-17   Study aim: To help us learn how text messaging can help adolescents between 13 and 17 years of age quit vaping. The Teen Quit Vaping Study (QVS Teen) is a comparative effectiveness trial to evaluate the effectiveness of a quit vaping text message program in promoting abstinence from e-cigarettes among young users aged 13-17.   Blinding: Double blind (Participant, Outcomes, Assessor)  Start date: 2021-10-01. End date: 2023-10-18 | |
| Participants | Total sample size: 1715  Age 13-17 (under 18); past 30-day use of nicotine vapes; interest in quitting vapes in the next 30 days; US residence. | |
| Interventions | Quit vaping text message intervention;  Assessment-only control condition;  waitlist control condition | |
| Outcomes | Nicotine vaping cessation at 7 months | |
| Identification | Amanda L. Graham, Truth Initiative Washington, DC 20001. agraham@truthinitiative.org  Title: Text message quit vaping intervention for adolescents NCT04919590 | |
| Study funding | Truth Initiative | |
| Notes | Study completed. Results available 7th August | |
| ***Risk of bias*** | | |
| **Bias** | **Authors' judgement** | **Support for judgement** |
| Random sequence generation (selection bias) | Unclear risk | Judgement Comment: "No information available (pre-publication)" |
| Allocation concealment (selection bias) | Unclear risk | Judgement Comment: "No information available (pre-publication)" |
| Blinding of outcome assessment (detection bias) All outcomes | Unclear risk | Judgement Comment: "Not enough information to make an assessment (pre-publication) " |
| Incomplete outcome data (attrition bias) All outcomes | Low risk | Judgement Comment: "521/759 in intervention group and 543/744 in control group followed up at 7 months. Greater than 50% follow-up and less than 20% difference between arms." |
| Selective reporting (reporting bias) | Unclear risk | Judgement Comment: "Outcomes pre-specified. However paper had not been viewed at the time of drafting this review." |
| Other bias | Low risk | Judgement Comment: "No evidence of additional risks of bias" |

Palmer 2023

| ***Study characteristics*** | | |
| Methods | Design: Randomized controlled trial. Pilot. Country: USA Setting: General public  Study aim: (1) evaluate reasons for quitting e-cigarettes in treatment-seeking adults, and (2) assess the feasibility and acceptability of nicotine replacement therapy (NRT) for e-cigarette cessation.  Recruitment: Participants were recruited to the study via online advertisements (Craigslist) across South Carolina. Advertisements were tailored explicitly for both e-cigarette-only (mono) users and dual-users inter­ested in quitting such that equal numbers of mono- (n = 15) and dual- users (n = 15) were recruited. Participants were given a link to an on­line survey via REDCap (Harris et al., 2009) to determine study eligi­bility, and all participants were screened over the phone to confirm. Start date: Enrollment June to December 2021. Pilot study completed | |
| Participants | Total sample size: 30 Intervention NRT + booklet n = 18. (10 EC users, 8 dual users.) Intervention at day 56 (FU) n=12. Control referral to quitline: n= 12. (5 EC users; 7 dual users.) Control at day 56 (FU) n=8  Study had 15 exclusive EC users and 15 dual users.  Inclusion criteria: (1) age 18+; (2) daily nicotine vaping (25 + days per month); (3) vaping 5 + times/day; (4) vaping ≥ 1 year; (5) interest in quitting vaping [and smoking, for dual users] within the next month (≥7 on 10-point scale); (6) willingness to try NRT; (7) able to receive text messages and to complete interviews over the phone or via video software; and (8) live in South Carolina  Exclusion criteria: used any other non-cigarette tobacco product or cessation medication, or endorsed a current medical condition that was contraindicated for NRT use.  Mean age 32 (SD 11). 50% female. Disposable device 36.6% (11 people). Main e-liquid flavour: Tobacco/Menthol; 15 (50%) ; Fruit/Other - 15 (50%) | |
| Interventions | Arm 1. NRT + supportive written materials (booklet) for e-cigarette cessation 28-day supply of combination NRT (21 mg patches, 4 mg lozenges) + supportive booklet (based CBT) adapted for vaping or dual cessation (2 different booklets) Participants asked to pick a day to quit vaping (and smoking, if applicable) within 1 week. Then mailed a 28-day supply of 21 mg nicotine patches, 4 mg lozenges, and a support booklet. The support booklet was developed by modifying the existing booklet utilized by the MUSC To¬bacco Treatment Program which was based on cognitive-behavioral therapy skills. Text was adapted to provide skills for EC cessation or dual product cessation, resulting in two separate booklets for each type of participant (one for mono-users detailing EC cessation, one for dual users detailing both smoking and EC cessation). On the specified quit day, participants received a supportive text message (“Today is your quit date! Make sure to use your medications and written materials to help you. You got this!”). After this, participants received daily surveys via text message over the course of 28 days. Text on quit date (within 1 week of baseline) then daily survey via text message over the course of 28 days. Daily reports of EC or CC use and AEs from NRT.  Arm 2. Control: SC Quitline referral Participants in the Control condition were provided with information about how to contact the SC Quitline (call, text, online) (South Carolina Tobacco Quitline, 2022) and encouraged to enroll in services within 1 week. Participants informed quitline offered pharmacotherapy, a vape cessation protocol (Live Vape Free Pro­gram, 2022), and smoking cessation support if applicable. The control group did not complete daily surveys   Both groups: Prior to trial onset, participants engaged in a brief interview over video. Participants were asked (1) “Tell me about why you are interested in quitting e-cigarettes (and cigarettes, if applicable).”; (2) “Have you talked to a medical pro¬fessional about your e-cigarette use? What was that like/what do you think it would be like?”; and (3) “What do you think the challenges will be when quitting e-cigarettes (and smoking)?” | |
| Outcomes | Baseline, day 28 (4 weeks), day 56 (8 weeks)  Adverse events. Number of participants reporting any type of adverse event(s). Only recorded in the intervention arm. Serious adverse events. Number of participants reporting any type of serious adverse event(s) | |
| Identification | Amanda M Palmer, Department of Public Health Sciences, Medical University of South Carolina. palmeram@musc.edu Title: Nicotine replacement therapy for vaping cessation among mono and dual users: A mixed methods preliminary study | |
| Study funding | "This study has been funded by NIH Institutional Postdoctoral Training Grant NIH-T32-HL144470 and the MUSC Hollings Cancer Center P30 CA138313. This funding source had no role in the design of this study and will not have any role during its execution, analyses, interpretation of the data, or decision to submit results. " | |
| Author declarations | "Dr. Toll has testified on behalf of plaintiffs who have filed litigation against the tobacco industry." " The authors declare that they have no known competing financial interests or personal relationships that could have appeared to influence the work reported in this paper." | |
| Notes | Information via email. 10 people experienced AE in the intervention arm. AEs were not recorded in the control arm. | |
| ***Risk of bias*** | | |
| **Bias** | **Authors' judgement** | **Support for judgement** |
| Random sequence generation (selection bias) | Unclear risk | Quote: "participants were randomized into study group at a target ~2:1 ratio; however, due  to the small sample size, this exact ratio was not achieved: Intervention  (n = 18), Control (n = 12)." Judgement Comment: "No details about the method of randomisation used." |
| Allocation concealment (selection bias) | Unclear risk | Judgement Comment: "No detail provided." |
| Blinding of participants and personnel (performance bias) All outcomes | High risk | Quote: "the control group did not complete daily surveys." Judgement Comment: "Different intervention intensity between intervention and control groups which might affect performance bias. No placebo." |
| Blinding of outcome assessment (detection bias) All outcomes | Low risk | Judgement Comment: "Outcomes were self-reported, but there was no face to face contact during the study." |
| Incomplete outcome data (attrition bias) All outcomes | Low risk | Judgement Comment: "Intervention 12/18. Control (quitline) 8/12. 80% completed the EOT survey (80 %). 66.6% completed the FU survey. Vaping and smoking status imputed as not abstinent for those with missing data." |
| Selective reporting (reporting bias) | Unclear risk | Judgement Comment: "Study was not pre-registered and presented as feasibility study with a focus on qualitative results" |
| Other bias | Low risk | Judgement Comment: "No other risk of bias noted." |

Rigotti 2024

| ***Study characteristics*** | | |
| Methods | Study design: RCT. Double-blind placebo-controlled randomized clinical trial  Country: USA  Setting: 5 US trial sites  Study aim: To determine the efficacy and safety of cytisinicline vs placebo to produce abstinence from e-cigarette use in adults seeking to quit vaping nicotine  Blinding: Masking: Quadruple (Participant Care Provider Investigator Outcomes Assessor)  Study length: 4 months. 3 months (12 weeks) of treatment. FU at 4 months (16 weeks).  Study start data: 13 July 2022. Study completion date: 28 February 2023 | |
| Participants | N = 160 Number per study group (arm): Cytisinicline = 107 (106 treated); Placebo 53. Randomized 2:1.  Withdrawals: 18 from Cytisinicline arm; 11 from placebo arm  Reason for withdrawals: Safety analyses excluded 1 participant assigned to cytisinicline who discontinued the study before receiving the drug.  Inclusion criteria: Adults 18 years and older, current daily use of a nicotine-containing vapes, had a positive (≥30 ng/mL) saliva cotinine test result. Willing to bring vape to study site so that specific product type, flavor and nicotine level can be documented. Intended to quit vaping, and were willing to set a quit date 7 to 14 days after starting the study drug and participate in vaping-cessation behavioral support.  Exclusion criteria: Dual users, of vape and combustible tobacco product. Past month use of smoking cessation medication (eg, bupropion, varenicline, nortriptyline, nicotine-replacement product); plan to use cigarettes or any nicotine-containing nonvaping product during study drug treatment; a positive urinary screen for illicit drugs (not including cannabis); health issues e.g. uncontrolled hypertension. User of cannabis asked to refrain from smoking cannabis during the study.  Overall sample. Mean age 33.6 (SD 11.1); 51.9% female; 71.9% formerly smoked greater or equal to 100 lifeime cigarettes.  Cytisine / Cytisinicline + behavioral support Main vaping device: Disposable 35.5%; Rechargeable 64.5%. [Rechargeable prefilled pod 30.8%; Rechargeable user-filled pod 16.8%; Rechargeable user-filled tank 16.8%] Main e-liquid flavour: Fruit 61 (57%); mint/menthol 36 (33.6%); candy, dessert or sweet 17 (15.9%); tobacco 11 (10.3%)  Placebo + behavioural support Main vaping device: Disposable 29 (54.7%); Rechargeable 24 (45.3%); [Rechargeable prefilled pod 14 (26.4%); Rechargeable user-filled pod 6 (11.3%); Rechargeable user-filled tank 4 (7.5%) Main e-liquid flavour: Fruit 33 (62%); mint/menthol 17 (32.1%); candy, dessert or sweet 3 (5.7%); tobacco 4 (7.5%)  No tobacco use at baseline, no tobacco use within 30 days. "To isolate the effects of cytisinicline for quitting nicotine vaping, we excluded dual users, defined as individuals who reported having smoked a cigarette or used another combustible or non-combustible tobacco product in the 4 weeks prior." | |
| Interventions | Arm 1. Cytisinicline + Behavioral Support Tablets containing 3mg of cytisinicline taken orally 3 times daily for 12 weeks. Behavioural support: At each of 13 visits from randomization to week 12, trained counselors offered all participants 10 minutes of brief vaping-cessation support (total of ≤130 minutes) using methods adapted from smoking cessation motivational and cognitive behavioral treatment.   Arm 2. Placebo + Behavioral Support Placebo tablet 3 x per day for 12 weeks. Behavioural support: Weekly, to week 12, 10 minutes of brief vaping-cessation support (total of ≤130 minutes, 13 sessions)(as per intervention arm). | |
| Outcomes | Baseline, weekly to week 12, then at 16 weeks.  Nicotine vaping cessation (cotinine verified); Serious adverse events. Number of participants reporting any type of serious adverse event(s); Adverse events. Number of participants reporting any type of adverse event(s); Combustible tobacco use (CO verified); Heart rate (bpm); Blood pressure. | |
| Identification | Sponsorship source: National Institute of Drug Abuse (#R44-DA054784) and Achieve Life Science Nancy A. Rigotti, Tobacco Research and Treatment Center, Harvard Medical School, nrigotti@mgh.harvard.edu Title: A multicenter, double-blind, randomized, placebo-controlled phase 2 trial evaluating the efficacy and safety of cytisinicline in adults using nicotine-containing e-cigarettes. Other title: Cytisinicline for vaping cessation in adults using nicotine e-cigarettes the ORCA-V1 randomized clinical trial Trial ID: NCT05431387 | |
| Study funding | "This work was supported by the National Institute of Drug Abuse (#R44-DA054784) and Achieve Life Science." | |
| Author declarations | Dr Rigotti reported personal fees from Achieve Life Sciences ending December 31, 2022, grants from Achieve Life Sciences paid to her institution, and personal fees from UpToDate for writing reviews of smoking cessation and e-cigarettes outside the submitted work. Dr Benowitz reported personal fees from Achieve Life Sciences during the conduct of the study as well as personal fees as a tobacco litigationexpert witness in litigation against tobacco companies outside the submitted work. Dr Prochaska reported grants from the National Institutes of Health during the conduct of the study as well as personal fees from Oneleaf Health, Achieve Life Sciences and plaintiff law firms in litigation against tobacco companies outside the submitted work. Mr Cain reported personal fees from Achieve Life Sciences and grants from the National Institutes of Health during the conduct of the study as well as personal fees from Achieve Life Sciences outside the submitted work and a patent (11083715) issued (Achieve Life Sciences; inventor). Ms Ball reported grants from the National Institutes of Health during the conduct of the study. Dr Clarke reported a patent for US20210330652A1 pending (inventor). Dr Blumenstein reported other support from Achieve Life Sciences during the conduct of the study. Dr Jacobs reported a patent (11083715) issued (Achieve Life Sciences) | |
| Notes | Published: https://jamanetwork.com/journals/jamainternalmedicine/fullarticle/2818194 Rigotti 2024 JAMA | |
| ***Risk of bias*** | | |
| **Bias** | **Authors' judgement** | **Support for judgement** |
| Random sequence generation (selection bias) | Low risk | Judgement Comment: "A predetermined central computer-generated randomization sequence assigned participants in a 2:1 ratio to receive cytisinicline or placebo." |
| Allocation concealment (selection bias) | Low risk | Quote: "A predetermined central computer-generated randomization sequence assigned participants in a 2:1 ratio to receive cytisinicline or placebo for 12 weeks" Judgement Comment: "Participants and study staff at all sites were blinded to study group assignment throughout the data collection period." |
| Blinding of participants and personnel (performance bias) All outcomes | Low risk | Quote: "The study medication was identical-appearing tablets containing 3mg of cytisinicline or placebo taken orally 3 times daily for 12 weeks" Judgement Comment: "Participants and study staff at all sites were blinded to study group assign- ment throughout the data collection period." |
| Blinding of outcome assessment (detection bias) All outcomes | Low risk | Quote: "participants... provided a breath sample to measure carbon monoxide and a saliva sample to measure cotinine." Judgement Comment: "Biochemically confirmed continuous EC or vaping abstinence. Cotinine measured." |
| Incomplete outcome data (attrition bias) All outcomes | Low risk | Judgement Comment: "Completed Cytisinicline 89/107 = 83% Placebo 42/53 = 79.2% > 50% at FU. <20 % difference between arms" |
| Selective reporting (reporting bias) | Low risk | Judgement Comment: "Outcomes were pre-registered and time-point and method of analysis pre-specified in the protocol" |
| Other bias | Low risk | Judgement Comment: "No other sources of bias noted." |

Sahr 2021

| ***Study characteristics*** | | |
| Methods | Design: Randomized controlled trial. Pilot Country: USA Setting: University (employees and students)  Recruitment: Informational fliers posted in Big Rapids, MI, USA and emails sent through a university’s listserv to employees and students  Study aim: To pilot three methods of Electronic Nicotine Delivery Systems (ENDS) cessation by measuring cessation rates, motivational techniques that contributed to cessation success, and participants’ changes after decreasing vape use. Blinding: "Due to the nature of the intervention, blinding was not done for the researcher or participant" | |
| Participants | Total sample size: 24 Number of withdrawals: 8 Number per study group (arm): NRT + behavioral support = 7 at baseline. At 6 moths = 4 participants Vape Taper + behavioral support = 8. At 6 months = 6 participants. Self guided = 9. At 6 months = 6 participants.  Inclusion criteria: Adults who used ENDS at least four days a week and were motivated to quit within two weeks  Nicotine Replacement Therapy (NRT) + Behavioral Support: mean age 22.6 (SD 7.3). 28.6% female. Vape-Taper + Behavioral Support : mean age 20 (SD 2.3). 12.5% female. Control. Self-Guided: mean age 19.4 (SD 1.5). 44.4% female | |
| Interventions | Baseline, 4, 8 & 12 weeks, 6 months  Nicotine Replacement Therapy (NRT) + Behavioral Support Pharmacist led behavioral support and nicotine patches and/or either nicotine gum or lozenges based on their personal preference. The NRT quit plan was determined based on their Fagerstrom Test for Nicotine Dependence score modified for vaping. All referred to the Michigan Tobacco Quitline.4, 8 & 12 week appointments. Pharmacist provided behavioral support.    Vape-Taper + Behavioral Support Participants used their own ENDS and e-juice.Received both behavioral support from a pharmacist and recommended nicotine vape-taper plan based on their current e-juice nicotine concentration and vaping habits. Vape taper aimed to decrease the amount of nicotine consumed by reducing concentration and frequency over time. Started at the participant’s current nicotine concentration of vape liquid. The first week the participant decreased vape exposure by one session per day or decreased the duration of sessions (~10–15% of time spent vaping), and, the second week, the participant decreased nicotine concentration of vape, about 20–25%, depending on available products. If unable to complete the step, then the same step was repeated until being successful before moving on. Steps were followed until the participant was vape-free and nicotine-free. All referred to the Michigan Tobacco Quitline. 4, 8 & 12 week appointments. Vaper taper plan for daily EC use.  Control. Self-Guided All referred to the Michigan Tobacco Quitline. Asked to become vape-free and nicotine-free within 12 weeks. Asked at each call and in-person appointment to discuss their quit attempt. 4, 8 & 12 week appointments.  Each participant in all three goups received a $20 gift card at 4-week, 8-week, and 12-week appointments to cover study-related expenses such as time, travel, and phone usage. Participants in the self-guided group and vape-taper group were expected to purchase their own vaping supplies and received an additional $40 gift card at enrollment including at 4 weeks and 8 weeks to cover out-of-pocket expenses. | |
| Outcomes | Baseline, 4, 8 & 12 weeks, 6 months  Nicotine vaping cessation; Mean modified FTMD vaping version; Mean e-liquid daily consumption(SD), mL/day; Blood pressure systolic; Heart rate (bpm); Weight: (pounds) | |
| Identification | Michelle Sahr, College of Pharmacy, Ferris State University, MichelleSahr@ferris.edu Start date: Recruitment May 2019-January 2020 Title: Pilot study of electronic nicotine delivery systems (ENDS) cessation methods | |
| Study funding | This research was funded by the Michigan Pharmacy Foundation and Ferris State Faculty Research Grant | |
| Author declarations | "The authors declare no conflict of interest. The funders had no role in the design of the study, in the collection, analyses, or interpretation of data, in the writing of the manuscript, or in the decision to publish the results" | |
| ***Risk of bias*** | | |
| **Bias** | **Authors' judgement** | **Support for judgement** |
| Random sequence generation (selection bias) | Low risk | Judgement Comment: "Block randomization was used to place eligible participants in one of three arms NRT (nicotine patch +/− nicotine lozenge or gum) + behavioral support, vape-taper + behavioral support, or self-guided quit] in a 1:1:1 ratio." |
| Allocation concealment (selection bias) | Low risk | Judgement Comment: "Block randomization was used to place eligible participants in one of three arms NRT (nicotine patch +/− nicotine lozenge or gum) + behavioral support, vape-taper + behavioral support, or self-guided quit] in a 1:1:1 ratio." |
| Blinding of participants and personnel (performance bias) All outcomes | Low risk | Judgement Comment: The 2 intervention arms were equally intensive. |
| Blinding of outcome assessment (detection bias) All outcomes | High risk | Judgement Comment: Cessation self-reported, not biochemically validated. Differential face-to-face contact between groups. |
| Incomplete outcome data (attrition bias) All outcomes | Low risk | Judgement Comment: "8/24 dropped out (< 50%). At 6 mths: NRT 4/7 (57%); Vaper taper 6/8 (75%); Control 6/9 (67%). < 20% difference between groups. |
| Selective reporting (reporting bias) | Low risk | Judgement Comment: "Outcomes were pre-registered and time-point and method of analysis pre-specified in the protocol, and all outcomes are reported and publicly available." |
| Other bias | Low risk | Judgement Comment: No other sources of bias noted |

## References to studies

### Caponnetto 2023 {published data only}

[doi: https://dx.doi.org/10.1186/s12916-023-02919-2]

- \*Caponnetto P, Campagna D, Ahluwalia JS, Russell C, Maglia M, Riela PM, et al. Varenicline and counseling for vaping cessation: a double-blind, randomized, parallel-group, placebo-controlled trial. BMC Medicine 2023;21(1):220. [DOI: 10.1186/s12916-023-02919-2]
- Caponnetto P, Campagna D, Ahluwalia JS, Russell C, Maglia M, Riela PM, et al. A double-blind, randomized, controlled phase III trial investigating efficacy and safety of varenicline for vaping cessation in adult users. medRxiv 2022 [Preprint]. [DOI: 10.1101/2022.12.20.22283715]
- Caponnetto P, Maglia M, Polosa R. Efficacy of smoking cessation with varenicline plus counselling for e-cigarettes users (VAREVAPE): a protocol for a randomized controlled trial. Contemporary Clinical Trials Communications 2019;15:100412. [DOI: 10.1016/j.conctc.2019.100412]
- Caponnetto P, Spicuzza L, Campagna D, Ahluwalia JS, Russell C, Maglia M, et al. Varenicline for smoking cessation in individuals who smoke cigarettes and use electronic cigarettes: a double-blind, randomised, placebo-controlled phase 3 trial. EClinicalMedicine 2023;66:102316. [DOI: 10.1016/j.eclinm.2023.102316]
- EUCTR2016-000339-42-IT. A randomized controlled trial to evaluate the efficacy of VARENICLINA to stop smoking in smokers of elettronic sigarette or in smokers who smoke both elettronic sigarette and classic sigarettes (VAREVAPE). https://trialsearch.who.int/Trial2.aspx?TrialID=EUCTR2016-000339-42-IT (accessed 22 July 2024).

### Fucito 2024 {published data only}

- \*Fucito LM, Baldassarri SR, Baker NL, Krishnan-Sarin S, Gray KM, Toll BA, et al. Varenicline for e-cigarette cessation in adults: a preliminary placebo-controlled randomized trial. American Journal of Preventive Medicine 2024 May 16 [Epub ahead of print]. [DOI: 10.1016/j.amepre.2024.04.007] [PMID: 38752949]
- Fucito L. A preliminary randomized, double-blind, placebo controlled clinical trial of varenicline in adults who use e-cigarettes. In: Society for Research on Nicotine and Tobacco (SRNT) 30th Annual Meeting, 2024 March 20-23; Edinburgh. 2024:PPS18-5.
- NCT05541497. Varenicline for treatment of e-cigarette dependence. https://clinicaltrials.gov/ct2/show/NCT05541497 (first received 15 September 2022).

### Graham 2021 {published data only}

[doi: https://dx.doi.org/10.2196/18327]

- Do EK, Tulsiani S, Edwards G, Cha S, Amato MS, Hair EC. Treatment-seeking young people enrolled in a United States vaping cessation intervention trial report high frequency of use and nicotine dependence. Preventive Medicine Reports 2023;36:102533. [DOI: 10.1016/j.pmedr.2023.102533]
- Graham AL, Amato MS, Cha S, Jacobs MA, Bottcher MM, Papandonatos GD. Effectiveness of a vaping cessation text message program among young adult e-cigarette users: a randomized clinical trial. JAMA Internal Medicine 2021;181(7):923-30. [DOI: 10.1001/jamainternmed.2021.1793]
- Graham AL, Cha S, Papandonatos GD, Amato MS, Jacobs MA, Abroms LC, et al. E-cigarette and combusted tobacco abstinence among young adults: secondary analyses from a U.S.-based randomized controlled trial of vaping cessation. Preventive Medicine 2022;165(Pt B):107119. [DOI: https://dx.doi.org/10.1016/j.ypmed.2022.107119]
- Graham AL, Jacobs MA, Amato MS, Cha S, Bottcher MM, Papandonatos GD. Effectiveness of a quit vaping text message program in promoting abstinence among young adult e-cigarette users: protocol for a randomized controlled trial. JMIR Research Protocols 2020;9(5):e18327. [DOI: 10.2196/18327]
- NCT04251273. Text message quit vaping intervention for young adults. https://clinicaltrials.gov/show/NCT04251273 (first received 31 January 2020).

### Klein 2024 {published data only}

- \*Klein EG, Shoben AB, Carpenter KM, Mullis K, Nemeth JM, Mayers E, et al. Clinical trial of a quitline vaping cessation intervention: baseline characteristics of young adult exclusive e-cigarette users seeking treatment. International Journal of Environmental Research and Public Health 2024;21(6):809. [DOI: 10.3390/ijerph21060809]
- Klein EG, Carpenter KM, Shoben AB, Mullis K, Nemeth J, Mayers E, et al. A quitline-based young adult vaping cessation trial examining the impacts of NRT and mhealth compenents. In: Society for Research on Nicotine and Tobacco (SRNT) 30th Annual Meeting, 2024 March 20-23; Edinburgh. 2024:SYM17-1.
- Mullis K, Vickerman K. Old dog, new tricks? NRT use and effectiveness for young adult vaping cessation. In: Society for Research on Nicotine and Tobacco (SRNT) 30th Annual Meeting, 2024 March 20-23; Edinburgh. 2024:POS2-105.
- Mullis M, Vickerman K. Mhealth program engagement and vaping cessation outcomes among young adults. In: Society for Research on Nicotine and Tobacco (SRNT) 30th Annual Meeting, 2024 March 20-23; Edinburgh. Edinburgh, 2024:PPS18-3.
- NCT04974580. Research and innovation to stop e-cigarette/vaping in young adults. https://clinicaltrials.gov/show/NCT04974580 (first received 23 July 2021).
- Nemeth J. Optimizing for behavioral health equity in a multicomponent quitline-delivered vaping cessation programme for young adults. In: Society for Research on Nicotine and Tobacco (SRNT) 30th Annual Meeting, 2024 March 20-23; Edinburgh. Edinburgh, 2024:POS2-119.

### NCT04602494 {published data only}

- NCT04602494. Varenicline for nicotine vaping cessation in non smoker vaper adolescents. https://clinicaltrials.gov/ct2/show/NCT04602494 (first received 26 October 2020).

### NCT04919590 {published data only}

- NCT04919590. Text message quit vaping intervention for adolescents. https://clinicaltrials.gov/study/NCT04919590 (first received 9 June 2021).

### Palmer 2023 {published data only}

[doi: https://dx.doi.org/10.1016/j.addbeh.2022.107579]

- Palmer AM, Carpenter MJ, Rojewski AM, Haire K, Baker NL, Toll BA. Nicotine replacement therapy for vaping cessation among mono and dual users: a mixed methods preliminary study. Addictive Behaviors 2023;139:107579. [DOI: 10.1016/j.addbeh.2022.107579]

### Rigotti 2024 {published and unpublished data}

- \*Rigotti NA, Benowitz NL, Prochaska JJ, Cain DF, Ball J, Clarke A, et al. Cytisinicline for vaping cessation in adults using nicotine e-cigarettes: the ORCA-V1 randomized clinical trial. JAMA Internal Medicine 2024 May 6 [Epub ahead of print]e241313. [DOI: 10.1001/jamainternmed.2024.1313]
- NCT05431387. A study of cytisinicline for vaping cessation in adult smokers. https://clinicaltrials.gov/ct2/show/NCT05431387 (first received 24 June 2022).
- Rigotti NS. Cytisinicline for vaping cessation among adult nicotine e-cigarette users: a multi-site randomized placebo controlled trial (ORCA-V1). In: Society for Research on Nicotine and Tobacco (SRNT) 30th Annual Meeting, 2024 March 20-23; Edinburgh. 2024:PPS18-4.

### Sahr 2021 {published data only}

[doi: https://dx.doi.org/10.3390/pharmacy9010021]

- Sahr M, Kelsh S, Blower M, Sohn M. Pilot study of electronic nicotine delivery systems (ENDS) cessation methods. Pharmacy (Basel, Switzerland) 2021;9(1):21. [DOI: 10.3390/pharmacy9010021]
