# Supplementary material for: Interventions for quitting vaping
Source: Cochrane Database Syst Rev. 2025 Jan 8;2025(1):CD016058. doi: 10.1002/14651858.CD016058.pub2 (PMC11706636; doi:10.1002/14651858.CD016058.pub2)
Supplement: Supplementary file 4 — Supplementary material 4 Characteristics of ongoing studies [file CD016058-SUP-04-characteristicsOfOngoingStudies.html]

Characteristics of ongoing studies


# Supplementary material 4 to: Interventions for quitting vaping

Butler AR, Lindson N, Livingstone-Banks J, Notley C, Turner T, Rigotti NA, Fanshawe TR, Dawkins L, Begh R, Wu AD, Brose L, Conde M, Simonavičius E, Hartmann-Boyce J
  
https://doi.org/10.1002/14651858.CD016058.pub2

The material in this section has been supplied by the author(s) for publication under a Licence for Publication and the author(s) are solely responsible for the material. Cochrane has reviewed this material, but Cochrane has not copyedited, formatted or proofread. Cochrane accordingly gives no representations or warranties of any kind in relation to, and accepts no liability for any reliance on or use of, such material.

Back to top

# Characteristics of ongoing studies

## Table of contents

- Studies ordered by Study ID
  - ACTRN12623000022662
  - Chadi 2023
  - Krishnan-Sarin 2024
  - Lyu 2022
  - McColgan 2024
  - NCT04146714
  - NCT04898075
  - NCT04946825
  - NCT05140915
  - NCT05586308
  - NCT05892445
  - NCT05897242
  - NCT05994209
  - NCT06027840
  - NCT06087328
  - NCT06142877
  - NCT06164678
  - NCT06196489
  - Sanchez 2023
  - Schuster 2023
- Footnotes
- References to studies

## Studies ordered by Study ID

ACTRN12623000022662

| Study name | The OurFutures Vaping Program: A cluster randomised controlled trial to evaluate the efficacy of a school-based eHealth intervention to prevent e-cigarette use among adolescents |
| Methods | RCT, parallel group.  11-15 year olds (<18)  Setting: School  Country: Australia  Study aim: The OurFutures Vaping Program is a universal school-based eHealth prevention program that aims to prevent the uptake, and reduce the use, of e-cigarettes among adolescents. The program is built on the effective “OurFutures” (formerly “Climate Schools”) prevention model which is based on social influence and social competence principles.  Blinding: Participating secondary schools will be randomly allocated to one of two groups: i) an active control group (usual health education) or ii) an intervention group (the OurFutures Vaping Program). |
| Participants | Target enrolment 3360  Inclusion criteria: Eligible participants will be all Year 7 and/or Year 8 students attending participating schools in 2023. Students will be required to be fluent in English, provide informed active consent, and only students who receive parental consent will be eligible to participate.  Exclusion criteria: Schools with fewer than 70 enrolled Year 7/8 students in 2023. -Schools based outside NSW, WA and QLD. |
| Interventions | Intervention: OurFutures Vaping Program. eHealth prevention program to prevent uptake, and reduce use, of e-cigarettes among adolescents. Year 7/8 health education classes. The program consists of 4x40-minute lessons (delivered one week apart over 4 weeks) consisting of a web-based cartoon component completed individually by students (approx. 20mins), followed by optional teacher-facilitated activities (e.g., quizzes, class discussions, role plays). Factsheets (designed specifically for this study) are provided after each lesson to summarise and reinforce key content.  Control: Active control group (usual health education) |
| Outcomes | Baseline, post-test (post-completion of 4-week intervention), 6-, 12-, 24- and 36-month follow-up.  Primary: Uptake of e-cigarette use. To assess this, students will be asked “Have you ever used a vape, even one or two puffs?” (Yes/No). The primary endpoint will be the 12-month follow-up.  Secondary: Intentions to use tobacco cigarettes in the next year; Frequency of e-cigarette use; Quantity of e-cigarette use (articipants who report vaping within the past 30 days are asked how how many sessions a day they vape); Anxiety symptoms (PROMIS Anxiety Paediatric Item Bank); Attitudes towards e-cigarettes; E-cigarette refusal skill techniques; Psychological distress (Kessler 6); Frequency of tobacco cigarette use; Quality of life (Child Health Utlity); Externalising symptoms (Strengths and Difficulties Questionnaire); Depressive symptoms. adolescent version of the Patient Health Questionnaire-8; Self-efficacy to resist peer pressure. Assessed using an adapted version of the Resistive Self-Regulatory Efficacy Scale; Internalising symptoms (Strengths and Difficulties Questionnaire); Quantity of tobacco cigarette use; Knowledge about e-cigarettes and tobacco cigarettes (assessed as a composite outcome); Motives to use e-cigarettes (adapted version of the Tobacco Motives Inventory); Intentions to use e-cigarettes in the next year (single item based on those used in our previous school-based trials); Resource utilisation (self-report resource use questionnaire); Wellbeing (Short Warwick–Edinburgh Mental Well-being Scale); Perceptions of Stress (Perceived Stress Scale); Uptake of tobacco cigarette use. Assessed using a single item: “Have you ever tried smoking a cigarette, even one or two puffs?” (Yes/No). |
| Starting date | Date first enrollment: 24/03/2023 |
| Contact information | Lauren Gardner, The University of Sydney, Level 6, Jane Foss Russell building (G02) University of Sydney NSW 2006 Australia.  lauren.gardner@sydney.edu.au |
| Notes | Funding source: Medical Research Future Fund (MRFF). |

Chadi 2023

| Study name | A brief digital screening and intervention tool for parental and adolescent tobacco and electronic cigarette use in pediatric medical care in Canada: protocol for a pilot randomized controlled trial (canCEASE) |
| Methods | RCT.  Single-center, pragmatic, single-blind, pilot RCT comparing the CEASE intervention to usual care (control condition).  Recruitment: various clinics within the Sainte-Justine University Hospital Centre, (Montreal, Quebec, Canada), including the general pediatrics clinic, the adolescent medicine clinic, and the orthopedics and sports medicine clinics.  Setting: Tertiary care paediatric hospital in Montreal, Quebec, Canada  Country: Canada  Study aim: To demonstrate the feasibility and evaluate the preliminary effectiveness of the CEASE program for parental smoking cessation and its adapted version for adolescent smoking cessation and adolescent and parental vaping cessation. |
| Participants | 130  Age group: Both under and over 18. Parents or guardians of children aged between 0 and 17 years, and adolescent patients aged between 14 and 17 year.  Participants may be dual users of vapes and tobacco cigarettes.  Inclusion: Parents or legal guardians of children 0-17 years old and who smoke or use nicotine vaping products. "User" will be defined as those who answer "Yes" to the screening questions: "Have you smoked a single cigarette, even a puff, in the past 7 days?" (smoking); "Have you used an e-cigarette or vaping device containing nicotine, even a puff, in the past 7 days? (vaping). For CEASE-A, adolescent patients aged 14-17 years who smoke or use nicotine vaping products will be considered. "Adolescent User" will be defined by those who answer "Yes" to the same screening question as parents. Parents will be eligible if 1) they are at least 18 years old, 2) their child is 0-17 years of age, 3) are attending a regular scheduled medical appointment, and 4) are sufficiently proficient in either French or English (able to read and answer a written questionnaire). Adolescents will be eligible if they are 14-17 years-old, meet criteria 3) and 4) above, and have provided informed consent (in Québec, adolescents aged 14 years can provide consent). Adolescents whose parent(s) (if present) are not agreeable to their participation will be excluded.  Exclusion: Families presenting to the clinic without a scheduled medical appointment will be excluded. There will be no other exclusion criteria. |
| Interventions | CEASE Intervention arm, behavioural intervention. CEASE (for parents) and CEASE-A (for adolescents) are evidence-based tobacco and vaping cessation interventions delivered in pediatric practices. CEASE and CEASE-A are based on the 5A's model of smoking cessation: Ask about smoking, Advise to quit, Assess readiness to quit, Assist with a quit plan and Arrange follow-up. Given that CEASE and CEASE-A are one-time interventions, "Arrange" is removed, and the fourth step "Assist" is divided into two parts: a) providing phone/text/app quit support and b) providing NRT.  Control usual care. The control condition will be care as is usually delivered in participating clinics with the possibility of receiving direct linkage with cessation services delivered via CEASE/CEASE-A at the end of the 6-month study period. Current practice does not include routine provision of assistance for parental/adolescent smoking or e-cigarette cessation (e.g., referral to quitlines, NRT prescription). |
| Outcomes | Baseline, 1, 3 and 6 months  Vaping abstinence and combustible cigarette abstinence at 6 months (7-day, self-reported and cotinine confirmed ).  Recruitment rates; feasibilty; at 12 mths. Retention at 6 months.  Secondary outcomes: attempts to quit; intent to quit at 6 months. |
| Starting date | Satrt dtate: 22 Feb 2023  Completion date: 10 June 2024 |
| Contact information | Nicholas Chadi, Sainte-Justine University Hospital Research Centre, Montreal, QC, QC, Canada.  o.drouin@umontreal.ca (Olivier Drouin) |
| Notes | Funding: This study is supported by a bridge grant from the Canadian Institutes of Health Research (CIHR). OD and NC are supported by Clinician Scholar Awards from the Fonds de recherche du Québec-Santé. MPS is supported by a Junior 2 Clinician Scholar Award from the Fonds de recherche du Québec-Santé. |

Krishnan-Sarin 2024

| Study name | Examining the use of a virtual youth-focussed, e-cigarette cessation intervention combining in-person CBT with abstinence-contingent incentives |
| Methods | Virtual RCT  Setting: virtual  Country: USA  Recruitment: recruited from local schools in Conneticut and online social media (Snapchat, Instagram, Facebook, TikTok) |
| Participants | 99 participants  47 M, 50 F, 2 non-binary; M=17.6 years old; using e-cigarettes on 6.8 + 0.5 days/week, with a baseline urine cotinine level of M=1348.2+1075.1 ng/mL.  Youth (aged 13-20) who wanted to quit using e-cigarettes and used e-cigarettes regularly (at least 4 days/week; urine cotinine levels > 200 ng/ml) |
| Interventions | 6 week virtual cessation trial.  Randomised to receive incentives that were contingent (salivary cotinine < 30ng/ml) or non-contingent (providing salivary samples) on abstinence.  All participants received 6 remote weekly CBT sessions which started one week prior to their quit date. CBT content was derived from a youth-focused CBT manual and provided by therapists who were trained and supervised weekly. Starting on quit day participants also used an online program (NuRelm, Inc.) to provide salivary cotinine tests every other day for the 1st 2 weeks, then 2x per week for the final 2 weeks, and were randomized to receive incentives that were contingent (salivary cotinine < 30ng/ml) or non-contingent (providing salivary samples) on abstinence. |
| Outcomes | Baseline, 1,3,6, 12 months.  Primary outcome 7-day point-prevalence abstinence at the end of treatment (EOT at six weeks of treatment) based on self-reports and verified biochemically (saliva cotinine < 30 ng/ml).  Biochemically confirmed self-reports of abstinence at EOT (6 weeks) are at 38%. |
| Starting date | Not stated. |
| Contact information | Suchitra Krishnan-Sarin, University School of Medicine, New Haven, CT, USA. |
| Notes | Funding: Nonprofit grant funding entity |

Lyu 2022

| Study name | Delivering vaping cessation interventions to adolescents and young adults on Instagram: protocol for a randomized controlled trial |
| Methods | Design: RCT, parallel assignment. Masking single (outcomes assessor)  Setting: Online (Instagram)  Country: USA  Recruitment: Adolescents and young adults aged 13–21 residing in California who have vaped at least once per week in the past 30 days will be recruited online via Facebook and other social media, augmented by outreach through community partners and youth serving organizations. |
| Participants | 500  Intervention group 250; Control group 250.  Inclusion: 1. English literacy;2. Age between 13-21 years; 3. Indicate they use social media “most” (≥4) days per week; 4. Have vaped at least once per week in the past 30 days; 5. Access to a computer or mobile phone with photo capability to verify abstinence from vaping; 6. considering quitting / interested in quitting within the next 6 months; 7. Reside in California. This is because the funder for this study, the California Tobacco Related Diseases Research Program, requires the research be conducted in California.  Exclusion: 1. No English literacy; 2. Age under 13 or over 21 years; 3. Insufficient social media use (3 or fewer days per week); 4. Have not vaped at least once per week in past 30 days; 5. No access to computer or mobile phone with photo capability to verify abstinence from vaping; 6. Not interested in or considering quitting within the next 6 months; 7. Not California residents. |
| Interventions | Social media intervention, Instagram with up to 3 posts per day for 30 days. Participants will be educated about signs of nicotine dependence and if they express interest in pharmacotherapy will be encouraged to access this through their personal healthcare providers. The Instagram groups will provide educational and social support, troubleshooting and advice about nicotine replacement therapy (NRT) or other forms of treatment.  Control Condition: no intervention. Directed to the Truth Initiative e-cigarette texting quit program. This innovative and free text message program was created with input from teens, college students and young adults who have attempted to, or successfully, quit e-cigarettes using text coaching methods |
| Outcomes | Baseline, 1, 3, 6 months  Point Prevalent Abstinence (PPA) from vaping.  Abstinence from all tobacco products.  Change in the number of participants: with reduction in vaping (by 50% or more); number of vape quit attempts. Change in response on: Stages of Change Questionnaire; Thoughts About Abstinence (TAA) Questionnaire; scores on the Ways of Quitting questionnaire (WOQ). |
| Starting date | 18 November 2021  18 June 2024 |
| Contact information | Pamela Ling, MD,University of California, San Francisco |
| Notes | Funding: California Tobacco Related Diseases Research Program |

McColgan 2024

| Study name | Challenges and strategies for recruitment and retention of exclusive e-cigarette users in clinical trials |
| Methods | RCT  Phase 3 study to compare nicotine mouth spray and placebo over 1 year for abstinence.  Country: Canada, UK, Germany. |
| Participants | 800  Inclusion criteria: exclusive e-cigarette users |
| Interventions | Nicotine mouth spray vs placebo.  Remote visits, behavioral support, and a motivational assessment at screening to encourage subject retention, and the recruitment strategy utilizes AI-powered targeted recruitment through social media platforms most popular with the target demographic. |
| Outcomes | Study length 12 months.  Vaping abstinence |
| Starting date | Ongoing |
| Contact information | Bryan John McColgan, Kenvue, Helsingborg, Sweden, |

NCT04146714

| Study name | Substance use screening to encourage behavior change among young people in primary care  Official title: Screening for Excessive Substance Use in the Waiting Room to Encourage Behavior Change Among Young People (YP-HEALTH): a Multi-center Randomized Controlled Trial in Primary Care |
| Methods | RCT, parallel-group randomized controlled trial  Setting: primary care  Country: Switzerland  Recruitment: primary care practices in the French-speaking part of Switzerland.  Aim: to evaluates whether completing a short screening questionnaire about health behaviours in the waiting room before a primary care consultation decreases excessive substance use in young people aged 14 to 24 years. |
| Participants | 840 (estimated) (approximately 20 per practice)  Both over 18 and under 18 (young people: 14-24 years old).  Inclusion criteria: Patients aged 14 to 24 years consulting at the participating primary care practice for any motive.  Exclusion Criteria: Acute illness requiring immediate attention of the physician; Severe mental health conditions requiring treatment in a specialized setting; Young person not consulting as a patient at the practice (e.g. accompanying friend or partner); Inability to read the trial information in French or to provide independent consent. |
| Interventions | Two different types of confidential pre-consultation screening surveys: one focusing on the assessment of binge drinking and other substance use (intervention group) and the other on physical activity (control group).Substance use questionnaire vs Physical activity questionnaire  Intervention group: Screening questionnaire about substance use based on the Detection of Alcohol and Drug Problems in Adolescents (DEP-ADO) survey.  Control group: Screening questionnaire about physical activity, based on the short version of the International Physical Activity Questionnaire (IPAQ). |
| Outcomes | Baseline and follow-up phone interviews at 3, 6 and 12 months.  Outcome at 3 months follow-up proportion of patients reporting binge drinking (≥1 episode) in the past 30 days.  Secondary outcomes will include the proportion of young people reporting smoking (≥ 1 cigarette a day), electronic cigarette use (≥ once a day) and/or excessive cannabis use (≥1 joint/week) in the past 30 days. Analysis will be by intention to treat and will take into account clustering of participants within practices. |
| Starting date | Estimated start date: 2025-01  Estimated study completion: 2027-12 |
| Contact information | Dagmar M Haller  dagmar.haller-hester@unige.ch |

NCT04898075

| Study name | Quit nicotine: e-cig cessation intervention |
| Methods | Design: RCT  Country: USA  Setting: High school |
| Participants | N=100 (estimated)  Intervention group (CM for nicotine abstinence + CBT) n=50  Control group (CBT) n=50)  13-19 (both under and over 18 years)  Inclusion: High school students between ages 13-20 years. Regular e-cigarette (vape) user (primary EC users with or without other tobacco use who report using EC at least 4 days/week and have urine cotinine levels ≥200 ng/ml).  Exclusion: Substance Use Dependence (SUD) on other psychoactive substances. Current diagnosis of psychosis. Current diagnosis of a significant mental health disorder that is not being treated. |
| Interventions | Intervention: 4-week long web-based, remote Contingency Management (CM) for nicotine abstinence plus weekly individualized Cognitive Behavioral Therapy (CBT). Participants will be paid increasing amounts of payment for each negative saliva cotinine test.  Control. Participants will be paid for providing saliva nicotine test, regardless of whether the test is positive or negative.  Both groups will receive weekly CBT during this time (2 sessions prior to quitting, 4 weekly sessions after quitting). |
| Outcomes | Baseline, 1, 3, 6, and 12 months.  Vaping cessation  Self reported of no EC usage during the 7 days prior and confirmed negativity with cotinine levels of 30ng/mL (Alere iScreen OFD Cotinine Saliva Test; Countrywide Testing). |
| Starting date | April 2021. Estimated completion January 2025 |
| Contact information | Suchitra Krishnan-Sarin, Yale University. |
| Notes | Study collaborator: American Heart Association. |

NCT04946825

| Study name | Quit smoking study for people who use e-cigarettes.  Official title: A Randomized Controlled Trial of Smoking Cessation Treatment for Young Adult Dual Users of Combustible and Electronic Cigarettes |
| Methods | Design: RCT  Country: USA  Recruitment: national advertising strategies |
| Participants | N= 390 (estimated)  Inclusion: Young adult (18-29). Smokes tobacco cigarettes. Uses electronic cigarettes. Interested in quitting tobacco |
| Interventions | Combination NRT plus text messages.  A) NRT plus text messages to quit CCs only, B) NRT plus text messages to quit CCs and ECs simultaneously, C) text messages alone to quit CCs only, or D) text messages alone to quit CCs and ECs simultaneously. |
| Outcomes | Baseline, 3 months. 6 month FU survey.  Vaping cessation (7-day point-prevalence abstinence 3 months), AEs, combustible cigarette quit attempts, cigarettes per day. |
| Starting date | Start date: 27 June 2021. Estimated completion date January 2024 |
| Contact information | Elias Klemperer, University of Vermont |
| Notes | Funding: not stated. |

NCT05140915

| Study name | **Vaper to vaper: a multimodal mobile peer driven intervention to support adolescents in quitting vaping (V2V)** |
| Methods | RCT parallell assignment  Country USA |
| Participants | 80 (estimated)  13-19 years old  Grade 9-12 at participating high schools; current e-cigarette user; smartphone. |
| Interventions | Interventions: (1) peer messages, written by current and former adolescent e-cigarette users and tailored by age and readiness-to-quit; (2) peer coaching, facilitated by texting; and (3) gamification, designed to motivate participation.  Active Comparator: Control. Written e-cigarette cessation materials by the Research Coordinator at study enrollment. Two pamphlets from Journeyworks, E-Cigarettes: 8 Things Everyone Should Know, and support in quitting, How to Quit Vaping. |
| Outcomes | 6 months  Abstinence from vaping as measured by saliva samples [Cotinine-validated 7-day point prevalence]  Investigators will assess the change in severity of nicotine addiction, as measured by the Hooked on Nicotine Checklist (HONC). The HONC is a 10-item instrument used to determine the onset and strength of tobacco dependence. |
| Starting date | 2022 Dec. Estimated completion date April 2025. |
| Contact information | Rajani Sadasivam and Lori Pbert, University of Massachusetts Chan Medical School |

NCT05586308

| Study name | Pilot study of incentive-based and media literacy informed approaches to improve vaping cessation |
| Methods | RCT. Parallell assignment  Country: USA |
| Participants | N=80 (estimated)  Aged 19-29; report vaping in the previous 30 days; access to internet/video chat/SMS text message; interested in quitting vaping in the next 30 days |
| Interventions | 4 arms  Media literacy. Participants will receive media literacy e-learning lessons and an evidence-based text-messaging support.  Financial incentive. Participants will receive financial incentive intervention and an evidence-based text-messaging support.  Combined. Participants will receive media literacy e-learning lessons, financial incentive, and an evidence-based text-messaging support program.  Active control. Participants in this arm will receive an evidence-based text-messaging support. |
| Outcomes | 3 months.  Biochemically verified vaping abstinence (negative results- < 30 ng/mL) will be measured by saliva cotinine samples at end of study (Week 12).  Self -reported vaping abstinence  Biomarkers of toxic exposures: 20 participants will provide the urine sample at the baseline and end-of-study visits (2 months after the target quit date). The biomarker classes may include: 1) cotinine and hydroxycotinine, and 2) creatinine.  Nicotine dependence index will be measured by the Penn State E-cigarette Dependence (PSECD) index at baseline and end-of-study |
| Starting date | Dec 2023. Estimated completion date Dec 2024 |
| Contact information | Tzeyu Michaud,University of Nebraska |

NCT05892445

| Study name | Impact of aversive warnings on e-cigarette cessation intentions and behaviors among young adults |
| Methods | RCT  Country: USA  Recruitment: market research firms |
| Participants | N=1000 (estimated)  18-29 year old, using vapes at least once per week. |
| Interventions | Intervention: Aversive visual health warnings about the potential health risks of e-cigarette use, delivered through the online survey platform  Control group will not receive any intervention and will complete the same survey as the intervention group |
| Outcomes | 3 months  Follow-up assessments at 3-months post-intervention to investigate impact of aversive visual health warnings on e-cigarette cessation among young adults, including the moderating effects of prior adverse event experience. |
| Starting date | Start date May 2025 |
| Contact information | Raphael Cuomo, University of California, San Diego |

NCT05897242

| Study name | ACT on vaping: digital therapeutic for young adult vaping cessation |
| Methods | RCT  Country: USA |
| Participants | N=61  Age 18-30; current weekly user of e-cigarette product(s); owns an Android phone or iPhone. |
| Interventions | Arm 1: ACT on Vaping smartphone app and text messaging program and receive incentivized text messages assessing their vaping status.  Arm 2: Incentivized text messages check-ins assessing their vaping status. |
| Outcomes | 3 months  Cotinine-confirmed 30-day point prevalence abstinence from all nicotine and tobacco.  Self-reported 24-hour quit attempt  Change in readiness to quit. Overall treatment satisfaction rating. |
| Starting date | Jan 4th 2024. Completion April 26 2024 |
| Contact information | Jaimee Heffner,Fred Hutch/University of Washington Cancer Consortium |

NCT05994209

| Study name | Testing the feasibility and acceptability of social media and digital therapeutics to decrease vaping behaviors |
| Methods | RCT  Country: USA |
| Participants | N=189 (estimated)  Current vaper product user (P30D). A desire to quit vaping and/or experiencing negative health outcomes due to vaping.  Vaping product user only (i.e., not using vaping as a means to support combustible smoking cessation) |
| Interventions | Intervention Group A: quitSTART Mobile App Intervention. quitSTART  Experimental Group B: quitSTARTMobile App Intervention PLUS Embedded Chatbot Feature  Control group: No Intervention. (Nationwide resource referral and intervention waitlist) |
| Outcomes | Baseline, 6 weeks, 3 months.  Vaping cessation. 7 day self-reported vaping abstinence at follow-up.  Nicotine dependence will be assessed using the 4-item Patient-Reported Outcomes Measurement Information System (PROMIS).  Risk perception related to vaping.  Intervention engagement |
| Starting date | Jan 2024 (estimated). Completion estimated March 2025. |
| Contact information | Patricia Cavazos-Rehg, pcavazos@wust.edu  Washington University School of Medicine |

NCT06027840

| Study name | Concurrent vs. sequential cessation of dual cigarette and e-cigarette use |
| Methods | Randomized parallel assignment  Country: USA |
| Participants | N=40 (estimated)  18 years and older  Inclusion: Report cigarette smoking, and e-cigarette use for at least the past 3 months. Smoke 5 or more cigarettes per day. Report e-cigarette use for at least 14 days in the past month. Interested in quitting both products in the next month and willing to set a quit date.  Exclusion: use of other tobacco or nicotine products besides cigarettes and e-cigarettes > once per week in the last 30 days. |
| Interventions | Varenicline 12 weeks + counselling + booklet  This study examines whether concurrent treatment for cigarettes and e-cigarettes in which an individual quits both products at the same time (QUIT-C) or sequential treatment in which an individual quits cigarettes first followed by e-cigarettes is more effective for quitting both products.  Arm 1 Experimental: QUIT-C (Concurrent). Treatment in this arm will emphasize CONCURRENT cessation of cigarettes and e-cigarettes. All participants will receive 12-weeks of varenicline, weekly individual counseling, and access to cessation resources including a guided self-change booklet and links to free text-based support. Counseling and cessation resources will emphasize CONCURRENT cessation.  Arm 2: Experimental: QUIT-S (Sequential). Treatment in this arm will focus on cessation of cigarettes FOLLOWED SEQUENTIALLY by cessation of e-cigarettes. All participants will receive 12-weeks of varenicline, weekly individual counseling, and access to cessation resources including a guided self-change booklet and links to free text-based support. Counseling and cessation resources will emphasize SEQUENTIAL cessation. |
| Outcomes | 3 months  E-Cigarette abstinence. 7-day point prevalence e-cigarette abstinence at Week 12, biochemically verified by urine cotinine  Cigarette abstinence, 7-day point prevalence cigarette abstinence at Week 12, biochemically verified by breath carbon monoxide.  Biomarkers of tobacco-related harm exposure. Primary urinary biomarkers of tobacco-related harm exposure will be assessed including NNAL (4-(methylnitrosamino)-1-(3-pyridyl)-1-butanol), a tobacco carcinogen linked to lung cancer risk. |
| Starting date | April 2024. Estimated completion date June 2025. |
| Contact information | Lisa Fucito, Yale University, lisa.fucito@yale.edu |

NCT06087328

| Study name | CAN-DOSE study: cessation with augmented nicotine for dual use of smoking and e-cigarettes |
| Methods | Randomised.  Country: USA |
| Participants | N=45 (estimated)  18 years and over; daily nicotine-containing vape user (25+ days per previous month); vape use 5+ times/day; vape use > 1year; smoking >1 cigarette on 5-7 days per week; interest in quitting smoking and e-cigarette within the next month (>7 on 10-point scale); willing to use NRT; able to receive text messages/email.  Exclusion: Individuals reporting current use of other nicotine-containing products and/or smoking cessation medications; vaping non-nicotine substances |
| Interventions | Active Comparator: Arm A. Regular Nicotine patch and lozenge dose. 21mg patch, qd + 4mg lozenge prn [minimum of 5 & up to 20 per day]  Active Comparator: Arm B. . Augmented Nicotine patch and lozenge dose21mg patch + 14mg patch qd + 4mg lozenge prn minimum of 5 & [up to 30 per day]  Active Comparator: Arm C. Augmented Nicotine patch and lozenge dose. 2 x 21mg patches qd + 4mg lozenges prn [minimum of 5 & up to 40 per day] |
| Outcomes | 4 weeks, 8 weeks  Adverse events between groups will be evaluated. NRT side effects will be captured during daily diary assessments using the Systematic Assessment for Treatment Emergent Events (SAFTEE)  Number of days participants self-report abstinence from both e-cigarettes and smoking (dual use abstinence). |
| Starting date | December 2023. Estimated completion: September 2024. |
| Contact information | Rachel Christian, chrisrac@musc.edu  Amanda Palmer, Medical University of South Carolina |

NCT06142877

| Study name | Effects of social media use on young adults' e-cigarette use |
| Methods | RCT  Country: USA |
| Participants | N=200 (estimated)  Age 18-25; daily social media use; smartphone; nicotine vaping on 1-19 days of the past 30 days |
| Interventions | Experimental: Social Media Use Reduction. Participants will be incentivized to reduce their social media use by a pre-specified percentage from baseline.  No Intervention: Social Media Use as Usual |
| Outcomes | Baseline, 1, 3, and 6 months.  Number of days the participant vaped nicotine in the past 30 days.  Episodes per vaping day. Puffs per vaping episode |
| Starting date | December 2023. Estimated completion March 2025 |
| Contact information | Erin A Vogel, erin-vogel@ouhsc.edu |

NCT06164678

| Study name | Vaping cessation using the Ottawa model for smoking cessation among e-cigarette users |
| Methods | RCT  Parallel assignment  Country: Canada |
| Participants | N=180 (estimated)  18 years or over; vaping at least once per week for the past four weeks. |
| Interventions | Arm 1. Experimental: Ottawa Model for Smoking Cessation  The OMSC group will receive counselling and NRT if they choose with follow-up calls to support medication titration. Participants will be provided with quit cards, which are pre-loaded with $300 worth of funds that can only be used by the assigned study participant to purchase NRT (if they choose).  For those in the intervention group, the study counsellor who is a trained Nicotine Addiction Treatment Specialist (NATS) will facilitate follow-up, monitor NRT use, and advise participants to titrate NRT dose as required based on their minimum daily nicotine intake. These counselling calls will be conducted at day 3, 7, 14, 21, 30, 60, 90, and 180 as is standard in OMSC for people who smoke and are interested in quitting. A diary will also be provided to the participants to track their usage.  Arm 2. Control: Usual Care. The usual care group will receive the initial counselling session but no further follow-up or NRT. They will be able to self-initiate a follow-up call if they choose. Participants will not be excluded if they choose to initiate NRT on their own at their own expense. |
| Outcomes | Baseline, 3, 6, 12 months  Vaping cessation (7 day point prevalence), baseline to 3 months.  Continuous abstinence rates at 1-day, 7-day, 1-month, 3-month, 6-month, and 12-month follow up.  Self-reported cessation will be measured by a self-administered salivary cotinine test. 12 month assessment. |
| Starting date | July 2024. Estimated completion December 2025 |
| Contact information | Evyanne Quirouette, equirouette@ottawaheart.ca  Nia Patel, niapatel@ottawaheart.ca  Hassan Mir, Ottawa Heart Institute Research Corporation |
| Notes | Sponsor: Ottawa Heart Institute Research Corporation |

NCT06196489

| Study name | Adapting an intervention for vaping in young veterans |
| Methods | Randomised  Country: USA |
| Participants | N=20  Inclusion: young adults (18-30 years old); daily e-cigarette user for at least the past 6 months; willing to enroll in a program to quit nicotine use within 30 days; smartphone/computer/tablet.  Exclusion: current use of combustible tobacco products at least weekly. |
| Interventions | Arm 1. Experimental: receive the adapted vaping cessation intervention by telephone.  Arm 2. Experimental: will receive the adapted vaping cessation intervention by video telehealth. |
| Outcomes | Baseline, 2 months, 3 month follow-up  Number of days using e-cigarettes in the past 7 days at the time of assessment  Number of e-cigarette uses per day.  Client Satisfaction Questionnaire (CSQ-8) |
| Starting date | January 2024. Estimated study completion June 2024. |
| Contact information | Neal M Doran, neal.doran@va.gov, Veterans Medical Research Foundation.  Collaborator University of California |

Sanchez 2023

| Study name | Supporting youth vaping cessation with the crush the crave smartphone app: protocol for a randomized controlled trial |
| Methods | RCT  Country: Canada  Recruitment: through the Vaping Dependence Cohort—an existing panel of youth enrolled in a prospective cohort study at the University of Toronto who provided consent for recontact in future studies at the Ontario Tobacco Research Unit. |
| Participants | N=600 (estimated)  Age 16-18 and19-29; nicotine vape user (used in the previous 30 days)  Arm 1: Crush the crave = 300 (estimated)  Arm 2: Control = 300 (estimated) |
| Interventions | App based intervention vs control  Intervention arm: Crush the Crave app for vaping cessation. enables users to customize a quit plan. As a tracker app, Crush the Crave monitors the amount of money saved and the number of vape-free days since the user’s quit date. The app tracks cravings and vaping habits. App displays supportive messages and images and links to evidence-based resources, such as quitlines  Control arm: email invitationto complete the baseline assessment, and follow-up assessment questionnaires asking about e-cigarette use and abstinence. After last follow up they will be invited to try the Crush the Crave app, if interested.  Each participant in the intervention and control arms will receive compensation (CAD $10 [US $7.46] electronic gift card) upon completion of the baseline survey questionnaire and each time they complete a follow-up survey throughout the course of the study. |
| Outcomes | Baseline, 3, 6, 9, and 12 months.  self-reported 30-day PPA at 3 months, operationalized as not having vaped, even a puff, in the last 30 days.  Intention to quit smoking in the next 6 months (yes or no), number of puffs per vaping session, number of vape sessions per day, and number of sessions in the past 30 days. |
| Starting date | Recruitment started March 4 2022. |
| Contact information | Michael Chaiton, Michael.Chaiton@camh.ca  Institute for Mental Health Policy Research Centre for Addiction and Mental Health, Toronto, Canada )N, M5s 2S1 |
| Notes | Trial Registration: OSF Registries osf.io/hmd87; https://doi.org/10.17605/OSF.IO/HMD87 |

Schuster 2023

| Study name | A randomized controlled trial of varenicline and brief behavioral counseling delivered by lay counselors for adolescent vaping cessation: study protocol. |
| Methods | RCT  Country: USA  Recruitment: local high schools and colleges and community and clinical setting |
| Participants | N=300  Aged 16-25; daily or near daily nicotine vaping for the prior ≥ 3 months ; Nicotine dependence as defined by a score ≥4 on the 10-item E-cigarette Dependence Inventory (ECDI), or report of persistent use despite negative consequences, or prior failed quit attempts; no regular combusted tobacco use in the past 2 months at enrollment and exhaled CO <10 ppm. |
| Interventions | Arm 1: varenicline (up to 1 mg BID for 12 weeks). Attend QuitVaping behavioral support sessions, completed in-person or via video-conferencing, once per week for 12 weeks. Be encouraged to sign up for This Is Quitting (TIQ), a text message vaping cessation program for adolescents.  Arm 2: identical placebo, up to 1 mg for 12 weeks, plus behavioral and texting support for adolescent vaping cessation (as for arm 1).  Arm 3: monitoring only. Receive NO drug intervention. Attend NO behavioral support sessions. Will not be encouraged to sign up for text message vaping cessation support. |
| Outcomes | Baseline, 1, 2, 3, 4, 5, 6 months  Weekly for groups 1 and 2.  Vaping abstinence at 3 months (Continuous 4-week Nicotine Vaping Abstinence at end of treatment).  Continuous Nicotine Vaping Abstinence over Study Weeks 9 to 24  Adverese events study weeks 1-12.  Change in Nicotine Withdrawal Scale (MNWS); Change in Vaping Craving (QVC)  Other biomarkers Urine NNAL, THC-COOH. |
| Starting date | StartJune 2022. Completion May 28 2024 |
| Contact information | Eden Evins and Randi Schuster, Massachusetts General Hospital, USA |
| Notes | Funding statement: Funds for this study are provided by National Institute on Drug Abuse (NIDA; R01DA052583). The funder had no role in study design, decision to publish, or preparation of the manuscript. |

## Footnotes

Funding source not cited.

Authors declare no conflicts of interests.

## References to studies

### ACTRN12623000022662 {published data only}

- ACTRN12623000022662. The OurFutures Vaping Program: a cluster randomised controlled trial to evaluate the efficacy of a school-based eHealth intervention to prevent e-cigarette use among adolescents. https://trialsearch.who.int/Trial2.aspx?TrialID=ACTRN12623000022662 2023;(accessed 23 July 2024).
- Gardner LA, Rowe AL, Stockings E, Champion KE, Hides L, McBride N et al. Study protocol of the Our Futures Vaping Trial: a cluster randomised controlled trial of a school-based eHealth intervention to prevent e-cigarette use among adolescents. BMC Public Health 2023 Apr 12;23(1):683. [DOI: 10.1186/s12889-023-15609-8]

### Chadi 2023 {published data only}

- Chadi N, Diamant E, Perez T, Al-Saleh A, Sylvestre M-P, O'Loughlin J et al. A brief digital screening and intervention tool for parental and adolescent tobacco and electronic cigarette use in pediatric medical care in Canada: protocol for a pilot randomized controlled trial. JMIR research protocols 2023;12(101599504):e47978-. [DOI: https://dx.doi.org/10.2196/47978]
- NCT05366790. A brief digital screening tool to address tobacco and e-cigarette use in pediatric medical care.. https://clinicaltrials.gov/ct2/show/NCT05366790 2022.

### Krishnan-Sarin 2024 {published data only}

- Krishnan-Sarin S, Kong G, Bold K, Davis D,  Liss T,  Lavallee H et al. Examining the use of a virtual youth-focussed, e-cigarette cessation intervention combining in-person CBT with abstinence-contingent incentives. In: Society for Research on Nicotine and Tobacco (SRNT) 30th Annual Meeting Edinburgh. Vol. SYM17-2. 20-23 March 2024.

### Lyu 2022 {published data only}

- \*Lyu JC, Olson SS, Ramo DE, Ling PM. Delivering vaping cessation interventions to adolescents and young adults on Instagram: protocol for a randomized controlled trial. BMC public health 2022;22(1):2311-. [DOI: https://dx.doi.org/10.1186/s12889-022-14606-7]
- NCT04707911. Social media intervention to stop nicotine and cannabis vaping among adolescents. https://clinicaltrials.gov/show/NCT04707911 (first received 8 July 2024).

### McColgan 2024 {published data only}

- McColgan BJ. Challenges and strategies for recruitment and retention of exclusive e-cigarette users in clinical trials. In: Society for Research on Nicotine and Tobacco (SRNT) 30th Annual Meeting Edinburgh. Vol. COM2-1. 20-23 March 2024.

### NCT04146714 {published data only}

- NCT04146714. Substance use screening to encourage behavior change among young people in primary care. https://clinicaltrials.gov/ct2/show/NCT04146714 2019;(accessed 22 July 2024).

### NCT04898075 {published data only}

- NCT04898075. Quit nicotine: e-cig cessation intervention. https://clinicaltrials.gov/show/NCT04898075 2021;(accessed 23 July 2024).

### NCT04946825 {published data only}

- NCT04946825. Quit smoking study for people who use e-cigarettes. https://clinicaltrials.gov/show/NCT04946825 2021;(accessed 23 July 2023).

### NCT05140915 {published data only}

- NCT05140915. Vaper to vaper: a multimodal mobile peer driven intervention to support adolescents in quitting vaping. https://clinicaltrials.gov/show/NCT05140915 2021;(accessed 23 July 2024).

### NCT05586308 {published data only}

- NCT05586308. Incentive-based and media literacy informed approaches to improve vaping cessation. https://clinicaltrials.gov/show/NCT05586308 2022;(accessed 23 July 2024).

### NCT05892445 {published data only}

- NCT05892445. Impact of aversive warnings on e-cigarette cessation. https://clinicaltrials.gov/show/NCT05892445 2023;(accessed 23 July 2024).

### NCT05897242 {published data only}

- NCT05897242. A smartphone application (ACT on vaping) for vaping cessation in young adults. https://clinicaltrials.gov/ct2/show/NCT05897242 2023;(accessed 23 July 2024).

### NCT05994209 {published data only}

- NCT05994209. Testing the feasibility and acceptability of social media and digital therapeutics to decrease vaping behaviors. https://clinicaltrials.gov/ct2/show/NCT05994209 2023;(accessed 22 July 2024).

### NCT06027840 {published data only}

- NCT06027840. Concurrent vs. sequential cessation of dual cigarette and e-cigarette use. https://clinicaltrials.gov/ct2/show/NCT06027840 2023;(accessed 22 July 2024).

### NCT06087328 {published data only}

- NCT06087328. CAN-DOSE study: cessation with augmented nicotine for dual use of smoking and e-cigarettes. https://clinicaltrials.gov/ct2/show/NCT06087328 2023;(accessed 22 July 2024).

### NCT06142877 {published data only}

- NCT06142877. Effects of social media use on young adults' e-cigarette use. https://clinicaltrials.gov/ct2/show/NCT06142877 2023;(accessed 22 July 2024).

### NCT06164678 {published data only}

- NCT06164678. Vaping cessation using the Ottawa model for smoking cessation among e-cigarette users. https://clinicaltrials.gov/ct2/show/NCT06164678 2023;(accessed 22 July 2024).

### NCT06196489 {published data only}

- NCT06196489. Adapting an intervention for vaping in young veterans. https://clinicaltrials.gov/ct2/show/NCT06196489 2023;(accessed 22 July 2024).

### Sanchez 2023 {published data only}

- Sanchez S, Deck Al, Baskerville NB, Chaiton M. Supporting youth vaping cessation with the crush the crave smartphone app: protocol for a randomized controlled trial. JMIR research protocols 2023;12(101599504):e42956. [DOI: https://dx.doi.org/10.2196/42956]
- Sanchez S. Effectiveness of the 'crush the crave' smartphone app on vaping cessation among youth and young adults: a randomized controlled trial. In: Society for Research on Nicotine and Tobacco (SRNT) 30th Annual Meeting Edinburgh. Vol. PPS18-6. 20-23 March 2024.

### Schuster 2023 {published data only}

- NCT05367492. Varenicline for nicotine vaping cessation in adolescents. https://clinicaltrials.gov/ct2/show/NCT05367492 2022;(accessed 22 July 2024).
- Schuster RM, Cather C, Pachas GN, Nielsen L, Iroegbulem V, Dufour J et al. A randomized controlled trial of varenicline and brief behavioral counseling delivered by lay counselors for adolescent vaping cessation: study protocol.. Frontiers in psychiatry 2023;14(101545006):1083791-. [DOI: https://dx.doi.org/10.3389/fpsyt.2023.1083791]
