# Supplementary material for: Interventions for quitting vaping
Source: Cochrane Database Syst Rev. 2025 Jan 8;2025(1):CD016058. doi: 10.1002/14651858.CD016058.pub2 (PMC11706636; doi:10.1002/14651858.CD016058.pub2)
Supplement: Supplementary file 5 — Supplementary material 5 Analyses [file CD016058-SUP-05-analyses.html]

Analyses


# Supplementary material 5 to: Interventions for quitting vaping

Butler AR, Lindson N, Livingstone-Banks J, Notley C, Turner T, Rigotti NA, Fanshawe TR, Dawkins L, Begh R, Wu AD, Brose L, Conde M, Simonavičius E, Hartmann-Boyce J
  
https://doi.org/10.1002/14651858.CD016058.pub2

The material in this section has been supplied by the author(s) for publication under a Licence for Publication and the author(s) are solely responsible for the material. Cochrane has reviewed this material, but Cochrane has not copyedited, formatted or proofread. Cochrane accordingly gives no representations or warranties of any kind in relation to, and accepts no liability for any reliance on or use of, such material.

Back to top

# Analyses

## Analysis group 1: Combination NRT versus no/minimal support

| Analysis or subgroup title | No. of studies | No. of participants | Statistical method | Effect size |
| --- | --- | --- | --- | --- |
| 1.1 Vaping cessation at 6 months or longer | 1 |  | Risk Ratio (M-H, Random, 95% CI) | Totals not selected |
| 1.2 Number of participants reporting SAEs | 1 |  | Risk Ratio (M-H, Random, 95% CI) | Totals not selected |
| 1.3 Vaping cessation at between 3 & 6 months | 2 | 524 | Risk Ratio (M-H, Random, 95% CI) | 0.93 [0.45, 1.93] |
| 1.4 Number of participants reporting AEs | 1 |  | Risk Ratio (M-H, Random, 95% CI) | Totals not selected |
| 1.5 Weight (lbs) at longest follow-up | 1 |  | Mean Difference (IV, Random, 95% CI) | Totals not selected |
| 1.6 Systolic blood pressure (mmHg) at longest follow-up | 1 |  | Mean Difference (IV, Random, 95% CI) | Totals not selected |
| 1.7 Heart rate (bpm) at longest follow-up | 1 |  | Mean Difference (IV, Random, 95% CI) | Totals not selected |

## Analysis group 2: Cytisine versus placebo

| Analysis or subgroup title | No. of studies | No. of participants | Statistical method | Effect size |
| --- | --- | --- | --- | --- |
| 2.1 Number of participants reporting SAEs | 1 |  | Risk Ratio (M-H, Random, 95% CI) | Totals not selected |
| 2.2 Vaping cessation at between 3 & 6 months | 1 |  | Risk Ratio (M-H, Random, 95% CI) | Totals not selected |
| 2.3 Change in combustible tobacco product use (tobacco cigarette use) at between 3 & 6 months | 1 |  | Risk Ratio (M-H, Random, 95% CI) | Totals not selected |
| 2.4 Number of participants reporting AEs | 1 |  | Risk Ratio (M-H, Random, 95% CI) | Totals not selected |
| 2.5 Mean change in systolic blood pressure (mmHg) | 1 |  | Mean Difference (IV, Random, 95% CI) | Totals not selected |
| 2.6 Mean change in diastolic blood pressure (mmHg) | 1 |  | Mean Difference (IV, Random, 95% CI) | Totals not selected |
| 2.7 Mean change in heart rate (bpm) | 1 |  | Mean Difference (IV, Random, 95% CI) | Totals not selected |
| 2.8 Cotinine (ng/ml) at longest follow-up | 1 |  | Mean Difference (IV, Random, 95% CI) | Totals not selected |

## Analysis group 3: Varenicline versus control

| Analysis or subgroup title | No. of studies | No. of participants | Statistical method | Effect size |
| --- | --- | --- | --- | --- |
| 3.1 Vaping cessation at 6 months or longer | 1 |  | Risk Ratio (M-H, Random, 95% CI) | Totals not selected |
| 3.2 Number of participants reporting SAEs | 3 | 130 | Risk Ratio (M-H, Random, 95% CI) | 2.60 [0.11, 62.16] |
| 3.3 Vaping cessation at between 3 & 6 months | 3 | 182 | Risk Ratio (M-H, Random, 95% CI) | 1.64 [0.98, 2.74] |
| 3.4 Number of participants reporting AEs | 3 | 130 | Risk Ratio (M-H, Random, 95% CI) | 1.19 [0.84, 1.68] |
| 3.5 Weight (lbs) at longest follow-up | 1 |  | Mean Difference (IV, Random, 95% CI) | Totals not selected |
| 3.6 Systolic blood pressure (mmHg) at longest follow-up | 1 |  | Mean Difference (IV, Random, 95% CI) | Totals not selected |
| 3.7 Diastolic blood pressure (mmHg) at longest follow-up | 1 |  | Mean Difference (IV, Random, 95% CI) | Totals not selected |
| 3.8 Heart rate (bpm) at longest follow-up | 1 |  | Mean Difference (IV, Random, 95% CI) | Totals not selected |

## Analysis group 4: Nicotine/vaping reduction versus minimal support

| Analysis or subgroup title | No. of studies | No. of participants | Statistical method | Effect size |
| --- | --- | --- | --- | --- |
| 4.1 Vaping cessation at 6 months or longer | 1 |  | Risk Ratio (M-H, Random, 95% CI) | Totals not selected |
| 4.2 Vaping cessation at between 3 & 6 months | 1 |  | Risk Ratio (M-H, Random, 95% CI) | Totals not selected |
| 4.3 Weight (lbs) at longest follow-up | 1 |  | Mean Difference (IV, Random, 95% CI) | Totals not selected |
| 4.4 Systolic blood pressure (mmHg) at longest follow-up | 1 |  | Mean Difference (IV, Random, 95% CI) | Totals not selected |
| 4.5 Heart rate (bpm) at longest follow-up | 1 |  | Mean Difference (IV, Random, 95% CI) | Totals not selected |

## Analysis group 5: Text message versus no/minimal support

| Analysis or subgroup title | No. of studies | No. of participants | Statistical method | Effect size |
| --- | --- | --- | --- | --- |
| 5.1 Vaping cessation at 6 months or longer (subgrouped by age) | 2 | 4091 | Risk Ratio (M-H, Random, 95% CI) | 1.32 [1.19, 1.47] |
| 5.1.1 Under 18 years | 1 | 1503 | Risk Ratio (M-H, Random, 95% CI) | 1.35 [1.17, 1.57] |
| 5.1.2 Both under and over 18 years | 1 | 2588 | Risk Ratio (M-H, Random, 95% CI) | 1.29 [1.11, 1.50] |
| 5.1.3 18 years and over | 0 | 0 | Risk Ratio (M-H, Random, 95% CI) | Not estimable |
| 5.2 Number of participants reporting SAEs | 1 |  | Risk Ratio (M-H, Random, 95% CI) | Totals not selected |
| 5.3 Vaping cessation at between 3 & 6 months | 1 |  | Risk Ratio (M-H, Random, 95% CI) | Totals not selected |
| 5.4 Number of participants reporting AEs | 1 |  | Risk Ratio (M-H, Random, 95% CI) | Totals not selected |

## Analysis group 6: Combination NRT + print-based self-help versus minimal support

| Analysis or subgroup title | No. of studies | No. of participants | Statistical method | Effect size |
| --- | --- | --- | --- | --- |
| 6.1 Number of participants reporting SAEs | 1 |  | Risk Ratio (M-H, Random, 95% CI) | Totals not selected |

## Analysis group 7: Combination NRT + text message versus no/minimal intervention

| Analysis or subgroup title | No. of studies | No. of participants | Statistical method | Effect size |
| --- | --- | --- | --- | --- |
| 7.1 Numbers of participants reporting SAEs | 1 |  | Risk Ratio (M-H, Random, 95% CI) | Totals not selected |
| 7.2 Vaping cessation at between 3 & 6 months | 1 |  | Risk Ratio (M-H, Random, 95% CI) | Totals not selected |
| 7.3 Number of participants reporting AEs | 1 |  | Risk Ratio (M-H, Random, 95% CI) | Totals not selected |

## Analysis group 8: Combination NRT versus nicotine/vaping reduction

| Analysis or subgroup title | No. of studies | No. of participants | Statistical method | Effect size |
| --- | --- | --- | --- | --- |
| 8.1 Vaping cessation at 6 months or longer | 1 |  | Risk Ratio (M-H, Random, 95% CI) | Totals not selected |
| 8.2 Vaping cessation at between 3 & 6 months | 1 |  | Risk Ratio (M-H, Random, 95% CI) | Totals not selected |
| 8.3 Weight (lbs) at longest follow-up | 1 |  | Mean Difference (IV, Random, 95% CI) | Totals not selected |
| 8.4 Systolic blood pressure (mmHg) at longest follow-up | 1 |  | Mean Difference (IV, Random, 95% CI) | Totals not selected |
| 8.5 Heart rate (bpm) at longest follow-up | 1 |  | Mean Difference (IV, Random, 95% CI) | Totals not selected |

## Analysis group 9: Combination NRT versus text message

| Analysis or subgroup title | No. of studies | No. of participants | Statistical method | Effect size |
| --- | --- | --- | --- | --- |
| 9.1 Numbers of participants reporting SAEs | 1 |  | Risk Ratio (M-H, Random, 95% CI) | Totals not selected |
| 9.2 Vaping cessation at between 3 & 6 months | 1 |  | Risk Ratio (M-H, Random, 95% CI) | Totals not selected |
| 9.3 Number of participants reporting AEs | 1 |  | Risk Ratio (M-H, Random, 95% CI) | Totals not selected |

# Figures and tables

Analysis 1.1: Vaping cessation at 6 months or longer


Study or Subgroup
Sahr 2021

Combination NRT
Events
2
Total
7

No/minimal support
Events
1
Total
9

Risk Ratio
M-H, Random, 95% CI
2.57 [0.29 , 22.93]

Risk Ratio
M-H, Random, 95% CI


0.01

0.1

1

10

100


Favours no/minimal support

Favours combination NRT


Analysis 1.2: Number of participants reporting SAEs


Study or Subgroup
Klein 2024

Combination NRT
Events
0
Total
248

Control
Events
0
Total
260

Risk Ratio
M-H, Random, 95% CI
Not estimable

Risk Ratio
M-H, Random, 95% CI


0.1

0.2

0.5

1

2

5

10


Favours combination NRT

Favours control


Analysis 1.3: Vaping cessation at between 3 & 6 months


Study or Subgroup
Klein 2024
Sahr 2021

Total
Total events:
Test for overall effect: Z = 0.19 (P = 0.85)
Test for subgroup differences: Not applicable

Heterogeneity: Tau² = 0.19; Chi² = 2.64, df = 1 (P = 0.10); I² = 62%

Combination NRT
Events
105
3
108
Total
248
7

255

Control
Events
91
7
98
Total
260
9

269
Weight
66.9%
33.1%

100.0%

Risk Ratio
M-H, Random, 95% CI
1.21 [0.97 , 1.51]
0.55 [0.22 , 1.39]

0.93 [0.45 , 1.93]

Risk Ratio
M-H, Random, 95% CI


0.1

0.2

0.5

1

2

5

10


Favours no/minimal support

Favours combination NRT


Analysis 1.4: Number of participants reporting AEs


Study or Subgroup
Klein 2024

Combination NRT
Events
66
Total
190

Control
Events
24
Total
189

Risk Ratio
M-H, Random, 95% CI
2.74 [1.79 , 4.17]

Risk Ratio
M-H, Random, 95% CI


0.1

0.2

0.5

1

2

5

10


Favours combination NRT

Favours control


Analysis 1.5: Weight (lbs) at longest follow-up


Study or Subgroup
Sahr 2021

Combination NRT
Mean [lb]
205.8
SD [lb]
46.86
Total
5

No/minimal support
Mean [lb]
171.88
SD [lb]
38.81
Total
6

Mean Difference
IV, Random, 95% CI [lb]
33.92 [-17.57 , 85.41]

Mean Difference
IV, Random, 95% CI [lb]


-100

-50

0

50

100


Higher with no/minimal support

Higher with combination NRT


Analysis 1.6: Systolic blood pressure (mmHg) at longest follow-up


Study or Subgroup
Sahr 2021

Combination NRT
Mean [mmHg]
136.2
SD [mmHg]
13.04
Total
5

No/minimal support
Mean [mmHg]
127.88
SD [mmHg]
6.94
Total
9

Mean Difference
IV, Random, 95% CI [mmHg]
8.32 [-3.98 , 20.62]

Mean Difference
IV, Random, 95% CI [mmHg]


-50

-25

0

25

50


Favours combination NRT

Favours no/minimal support


Analysis 1.7: Heart rate (bpm) at longest follow-up


Study or Subgroup
Sahr 2021

Combination NRT
Mean [bpm]
71.4
SD [bpm]
17.6
Total
5

No/minimal support
Mean [bpm]
75.63
SD [bpm]
19.62
Total
9

Mean Difference
IV, Random, 95% CI [bpm]
-4.23 [-24.29 , 15.83]

Mean Difference
IV, Random, 95% CI [bpm]


-50

-25

0

25

50


Favours combination NRT

Favours no/minimal support


Analysis 2.1: Number of participants reporting SAEs


Study or Subgroup
Rigotti 2024

Cytisine
Events
0
Total
106

Placebo
Events
0
Total
53

Risk Ratio
M-H, Random, 95% CI
Not estimable

Risk Ratio
M-H, Random, 95% CI


0.5

0.7

1

1.5

2


Favours cytisine

Favours placebo


Analysis 2.2: Vaping cessation at between 3 & 6 months


Study or Subgroup
Rigotti 2024

Cytisine
Events
25
Total
107

Placebo
Events
7
Total
53

Risk Ratio
M-H, Random, 95% CI
1.77 [0.82 , 3.82]

Risk Ratio
M-H, Random, 95% CI


0.1

0.2

0.5

1

2

5

10


Favours placebo

Favours cytisine


Analysis 2.3: Change in combustible tobacco product use (tobacco cigarette use) at between 3 & 6 months


Study or Subgroup
Rigotti 2024

Cytisine
Events
8
Total
107

Placebo
Events
6
Total
53

Risk Ratio
M-H, Random, 95% CI
0.66 [0.24 , 1.81]

Risk Ratio
M-H, Random, 95% CI


0.1

0.2

0.5

1

2

5

10


Favours cytisine

Favours placebo


Analysis 2.4: Number of participants reporting AEs


Study or Subgroup
Rigotti 2024

Cytisine
Events
54
Total
106

Placebo
Events
29
Total
53

Risk Ratio
M-H, Random, 95% CI
0.93 [0.68 , 1.27]

Risk Ratio
M-H, Random, 95% CI


0.5

0.7

1

1.5

2


Favours cytisine

Favours placebo


Analysis 2.5: Mean change in systolic blood pressure (mmHg)


Study or Subgroup
Rigotti 2024

Cytisine
Mean [mmHg]
0.3
SD [mmHg]
12.96
Total
89

Placebo
Mean [mmHg]
-0.6
SD [mmHg]
10.74
Total
41

Mean Difference
IV, Random, 95% CI [mmHg]
0.90 [-3.35 , 5.15]

Mean Difference
IV, Random, 95% CI [mmHg]


-10

-5

0

5

10


Favours cytisine

Favours placebo


Analysis 2.6: Mean change in diastolic blood pressure (mmHg)


Study or Subgroup
Rigotti 2024

Cytisine
Mean [mmHg]
-1.2
SD [mmHg]
9.32
Total
89

Placebo
Mean [mmHg]
1.3
SD [mmHg]
8.41
Total
41

Mean Difference
IV, Random, 95% CI [mmHg]
-2.50 [-5.72 , 0.72]

Mean Difference
IV, Random, 95% CI [mmHg]


-10

-5

0

5

10


Favours cytisine

Favours placebo


Analysis 2.7: Mean change in heart rate (bpm)


Study or Subgroup
Rigotti 2024

Cytisine
Mean [bpm]
3
SD [bpm]
11.56
Total
89

Placebo
Mean [bpm]
2.4
SD [bpm]
12.2
Total
41

Mean Difference
IV, Random, 95% CI [bpm]
0.60 [-3.84 , 5.04]

Mean Difference
IV, Random, 95% CI [bpm]


-10

-5

0

5

10


Favours cytisine

Favours placebo


Analysis 2.8: Cotinine (ng/ml) at longest follow-up


Study or Subgroup
Rigotti 2024

Cytisine
Mean [ng/mL]
181.74
SD [ng/mL]
188.91
Total
87

Placebo
Mean [ng/mL]
211.69
SD [ng/mL]
199.37
Total
39

Mean Difference
IV, Random, 95% CI [ng/mL]
-29.95 [-104.05 , 44.15]

Mean Difference
IV, Random, 95% CI [ng/mL]


-100

-50

0

50

100


Favours cytisine

Favours placebo


Analysis 3.1: Vaping cessation at 6 months or longer


Study or Subgroup
Caponnetto 2023

Varenicline
Events
24
Total
70

Placebo
Events
12
Total
70

Risk Ratio
M-H, Random, 95% CI
2.00 [1.09 , 3.68]

Risk Ratio
M-H, Random, 95% CI


0.1

0.2

0.5

1

2

5

10


Favours placebo

Favours varenicline


Analysis 3.2: Number of participants reporting SAEs


Study or Subgroup
Caponnetto 2023
Fucito 2024
NCT04602494

Total
Total events:
Test for overall effect: Z = 0.59 (P = 0.56)
Test for subgroup differences: Not applicable

Heterogeneity: Not applicable

Varenicline
Events
1
0
0
1
Total
51
18
1

70

Control
Events
0
0
0
0
Total
44
15
1

60
Weight
100.0%

100.0%

Risk Ratio
M-H, Random, 95% CI
2.60 [0.11 , 62.16]
Not estimable
Not estimable

2.60 [0.11 , 62.16]

Risk Ratio
M-H, Random, 95% CI


0.01

0.1

1

10

100


Favours varenicline

Favours placebo


Analysis 3.3: Vaping cessation at between 3 & 6 months


Study or Subgroup
Caponnetto 2023
Fucito 2024
NCT04602494

Total
Total events:
Test for overall effect: Z = 1.88 (P = 0.06)
Test for subgroup differences: Not applicable

Heterogeneity: Tau² = 0.03; Chi² = 2.24, df = 2 (P = 0.33); I² = 11%

Varenicline
Events
28
8
0
36
Total
70
20
1

91

Control
Events
14
6
1
21
Total
70
20
1

91
Weight
64.6%
31.4%
4.1%

100.0%

Risk Ratio
M-H, Random, 95% CI
2.00 [1.15 , 3.46]
1.33 [0.57 , 3.14]
0.33 [0.03 , 4.19]

1.64 [0.98 , 2.74]

Risk Ratio
M-H, Random, 95% CI


0.01

0.1

1

10

100


Favours control

Favours varenicline


Analysis 3.4: Number of participants reporting AEs


Study or Subgroup
Caponnetto 2023
Fucito 2024
NCT04602494

Total
Total events:
Test for overall effect: Z = 0.95 (P = 0.34)
Test for subgroup differences: Not applicable

Heterogeneity: Tau² = 0.02; Chi² = 2.37, df = 2 (P = 0.30); I² = 16%

Varenicline
Events
36
12
1
49
Total
51
18
1

70

Control
Events
29
5
1
35
Total
44
15
1

60
Weight
73.8%
17.3%
8.9%

100.0%

Risk Ratio
M-H, Random, 95% CI
1.07 [0.81 , 1.41]
2.00 [0.91 , 4.39]
1.00 [0.32 , 3.10]

1.19 [0.84 , 1.68]

Risk Ratio
M-H, Random, 95% CI


0.1

0.2

0.5

1

2

5

10


Favours varenicline 

Favours placebo


Analysis 3.5: Weight (lbs) at longest follow-up


Study or Subgroup
Caponnetto 2023

Varenicline
Mean [lb]
169.32
SD [lb]
31.97
Total
51

Placebo
Mean [lb]
172.62
SD [lb]
31.09
Total
44

Mean Difference
IV, Random, 95% CI [lb]
-3.30 [-16.00 , 9.40]

Mean Difference
IV, Random, 95% CI [lb]


-50

-25

0

25

50


Higher with placebo

Higher with varenicline


Analysis 3.6: Systolic blood pressure (mmHg) at longest follow-up


Study or Subgroup
Caponnetto 2023

Varenicline
Mean [mmHg]
124.4
SD [mmHg]
8.3
Total
51

Placebo
Mean [mmHg]
126
SD [mmHg]
8.2
Total
44

Mean Difference
IV, Random, 95% CI [mmHg]
-1.60 [-4.93 , 1.73]

Mean Difference
IV, Random, 95% CI [mmHg]


-10

-5

0

5

10


Favours varenicline

Favours placebo


Analysis 3.7: Diastolic blood pressure (mmHg) at longest follow-up


Study or Subgroup
Caponnetto 2023

Varenicline
Mean [mmHg]
78
SD [mmHg]
8.9
Total
51

Placebo
Mean [mmHg]
77.2
SD [mmHg]
8.4
Total
44

Mean Difference
IV, Random, 95% CI [mmHg]
0.80 [-2.68 , 4.28]

Mean Difference
IV, Random, 95% CI [mmHg]


-10

-5

0

5

10


Favours varenicline

Favours placebo


Analysis 3.8: Heart rate (bpm) at longest follow-up


Study or Subgroup
Caponnetto 2023

Varenicline
Mean [bpm]
74.5
SD [bpm]
8.9
Total
51

Placebo
Mean [bpm]
76.7
SD [bpm]
12.2
Total
44

Mean Difference
IV, Random, 95% CI [bpm]
-2.20 [-6.55 , 2.15]

Mean Difference
IV, Random, 95% CI [bpm]


-10

-5

0

5

10


Favours varenicline

Favours placebo


Analysis 4.1: Vaping cessation at 6 months or longer


Study or Subgroup
Sahr 2021

Reduction in nicotine concentration and vape frequency
Events
3
Total
8

No/minimal support
Events
1
Total
9

Risk Ratio
M-H, Random, 95% CI
3.38 [0.43 , 26.30]

Risk Ratio
M-H, Random, 95% CI


0.02

0.1

1

10

50


Favours no/minimal support

Favours vaping reduction


Analysis 4.2: Vaping cessation at between 3 & 6 months


Study or Subgroup
Sahr 2021

Reduction in nicotine concentration and vape frequency
Events
6
Total
8

No/minimal support
Events
7
Total
9

Risk Ratio
M-H, Random, 95% CI
0.96 [0.57 , 1.64]

Risk Ratio
M-H, Random, 95% CI


0.5

0.7

1

1.5

2


Favours no/minimal support

Favours vaping reduction


Analysis 4.3: Weight (lbs) at longest follow-up


Study or Subgroup
Sahr 2021

Reduction in nicotine concentration and vape frequency
Mean [lb]
185
SD [lb]
31.73
Total
6

No/minimal support
Mean [lb]
171.88
SD [lb]
38.81
Total
6

Mean Difference
IV, Random, 95% CI [lb]
13.12 [-26.99 , 53.23]

Mean Difference
IV, Random, 95% CI [lb]


-100

-50

0

50

100


Higher with no/minimal support

Higher with vaping reduction


Analysis 4.4: Systolic blood pressure (mmHg) at longest follow-up


Study or Subgroup
Sahr 2021

Reduction in nicotine concentration and vape frequency
Mean [mmHg]
129.33
SD [mmHg]
13.16
Total
6

No/minimal support
Mean [mmHg]
127.88
SD [mmHg]
6.94
Total
9

Mean Difference
IV, Random, 95% CI [mmHg]
1.45 [-10.01 , 12.91]

Mean Difference
IV, Random, 95% CI [mmHg]


-20

-10

0

10

20


Favours vaping reduction

Favours no/minimal support


Analysis 4.5: Heart rate (bpm) at longest follow-up


Study or Subgroup
Sahr 2021

Reduction in nicotine concentration and vape frequency
Mean [bpm]
71.83
SD [bpm]
12.83
Total
6

No/minimal support
Mean [bpm]
75.63
SD [bpm]
19.62
Total
9

Mean Difference
IV, Random, 95% CI [bpm]
-3.80 [-20.22 , 12.62]

Mean Difference
IV, Random, 95% CI [bpm]


-20

-10

0

10

20


Favours vaping reduction

Favours no/minimal support


Analysis 5.1: Vaping cessation at 6 months or longer (subgrouped by age)


Study or Subgroup

5.1.1 Under 18 years
NCT04919590

Subtotal
Total events:
Test for overall effect: Z = 4.02 (P < 0.0001)

Heterogeneity: Not applicable

5.1.2 Both under and over 18 years
Graham 2021

Subtotal
Total events:
Test for overall effect: Z = 3.37 (P = 0.0007)

Heterogeneity: Not applicable

5.1.3 18 years and over

Subtotal
Total events:
Test for overall effect: Not applicable

Heterogeneity: Not applicable

Total
Total events:
Test for overall effect: Z = 5.24 (P < 0.00001)
Test for subgroup differences: Chi² = 0.17, df = 1 (P = 0.68), I² = 0%

Heterogeneity: Tau² = 0.00; Chi² = 0.17, df = 1 (P = 0.68); I² = 0%

Text message-based intervention
Events
287
287
314
314
0
601
Total
759

759
1304

1304

0

2063

No/minimal support
Events
208
208
239
239
0
447
Total
744

744
1284

1284

0

2028
Weight
50.8%

50.8%
49.2%

49.2%

100.0%

Risk Ratio
M-H, Random, 95% CI
1.35 [1.17 , 1.57]

1.35 [1.17 , 1.57]
1.29 [1.11 , 1.50]

1.29 [1.11 , 1.50]

Not estimable

1.32 [1.19 , 1.47]

Risk Ratio
M-H, Random, 95% CI


0.5

0.7

1

1.5

2


Favours no/minimal support

Favours text message-based intervention


Analysis 5.2: Number of participants reporting SAEs


Study or Subgroup
Klein 2024

Text message intervention
Events
0
Total
248

Control
Events
0
Total
260

Risk Ratio
M-H, Random, 95% CI
Not estimable

Risk Ratio
M-H, Random, 95% CI


0.01

0.1

1

10

100


Favours text message-based 

Favours no/minimal support


Analysis 5.3: Vaping cessation at between 3 & 6 months


Study or Subgroup
Klein 2024

Text message intervention
Events
98
Total
248

Control
Events
98
Total
260

Risk Ratio
M-H, Random, 95% CI
1.05 [0.84 , 1.31]

Risk Ratio
M-H, Random, 95% CI


0.5

0.7

1

1.5

2


Favours control

Favours text message intervention


Analysis 5.4: Number of participants reporting AEs


Study or Subgroup
Klein 2024

Text message intervention
Events
43
Total
181

Control
Events
47
Total
198

Risk Ratio
M-H, Random, 95% CI
1.00 [0.70 , 1.44]

Risk Ratio
M-H, Random, 95% CI


0.2

0.5

1

2

5


Favours text message-based intervention

Favours no/minimal support


Analysis 6.1: Number of participants reporting SAEs


Study or Subgroup
Palmer 2023

Combination NRT + print-based self-help
Events
0
Total
12

No/minimal support
Events
0
Total
11

Risk Ratio
M-H, Random, 95% CI
Not estimable

Risk Ratio
M-H, Random, 95% CI


0.01

0.1

1

10

100


Favours combination NRT + print-based self-help

Favours minimal support


Analysis 7.1: Numbers of participants reporting SAEs


Study or Subgroup
Klein 2024

Combination NRT + text message-based intervention
Events
0
Total
122

No/minimal support
Events
0
Total
134

Risk Ratio
M-H, Random, 95% CI
Not estimable

Risk Ratio
M-H, Random, 95% CI


0.1

0.2

0.5

1

2

5

10


Favours combination NRT + text messages

Favours no/minimal support


Analysis 7.2: Vaping cessation at between 3 & 6 months


Study or Subgroup
Klein 2024

Combination NRT + text message-based intervention
Events
53
Total
122

No/minimal support
Events
46
Total
134

Risk Ratio
M-H, Random, 95% CI
1.27 [0.93 , 1.72]

Risk Ratio
M-H, Random, 95% CI


0.5

0.7

1

1.5

2


Favours no/minimal support

Favours combination NRT + text messages


Analysis 7.3: Number of participants reporting AEs


Study or Subgroup
Klein 2024

Combination NRT + text message-based intervention
Events
33
Total
94

No/minimal support
Events
14
Total
102

Risk Ratio
M-H, Random, 95% CI
2.56 [1.46 , 4.47]

Risk Ratio
M-H, Random, 95% CI


0.1

0.2

0.5

1

2

5

10


Favours combination NRT + text messages

Favours no/minimal support


Analysis 8.1: Vaping cessation at 6 months or longer


Study or Subgroup
Sahr 2021

Combination NRT
Events
2
Total
7

Reduction in nicotine concentration and vape frequency
Events
3
Total
8

Risk Ratio
M-H, Random, 95% CI
0.76 [0.17 , 3.33]

Risk Ratio
M-H, Random, 95% CI


0.1

0.2

0.5

1

2

5

10


Favours vaping reduction

Favours combination NRT


Analysis 8.2: Vaping cessation at between 3 & 6 months


Study or Subgroup
Sahr 2021

Combination NRT
Events
3
Total
7

Reduction in nicotine concentration and vape frequency
Events
6
Total
8

Risk Ratio
M-H, Random, 95% CI
0.57 [0.22 , 1.47]

Risk Ratio
M-H, Random, 95% CI


0.1

0.2

0.5

1

2

5

10


Favours vaping reduction

Favours combination NRT


Analysis 8.3: Weight (lbs) at longest follow-up


Study or Subgroup
Sahr 2021

Combination NRT
Mean [lb]
205.8
SD [lb]
46.86
Total
5

Reduction in nicotine concentration and vape frequency
Mean [lb]
185
SD [lb]
31.73
Total
6

Mean Difference
IV, Random, 95% CI [lb]
20.80 [-27.49 , 69.09]

Mean Difference
IV, Random, 95% CI [lb]


-100

-50

0

50

100


Higher with vaping reduction

Higher with combination NRT


Analysis 8.4: Systolic blood pressure (mmHg) at longest follow-up


Study or Subgroup
Sahr 2021

Combination NRT
Mean [mmHg]
136.2
SD [mmHg]
13.04
Total
5

Reduction in nicotine concentration and vape frequency
Mean [mmHg]
129.33
SD [mmHg]
13.16
Total
6

Mean Difference
IV, Random, 95% CI [mmHg]
6.87 [-8.67 , 22.41]

Mean Difference
IV, Random, 95% CI [mmHg]


-50

-25

0

25

50


Favours combination NRT

Favours vaping reduction


Analysis 8.5: Heart rate (bpm) at longest follow-up


Study or Subgroup
Sahr 2021

Combination NRT
Mean [bpm]
71.4
SD [bpm]
17.6
Total
5

Reduction in nicotine concentration and vape frequency
Mean [bpm]
71.83
SD [bpm]
12.83
Total
6

Mean Difference
IV, Random, 95% CI [bpm]
-0.43 [-18.96 , 18.10]

Mean Difference
IV, Random, 95% CI [bpm]


-50

-25

0

25

50


Favours combination NRT

Favours vaping reduction


Analysis 9.1: Numbers of participants reporting SAEs


Study or Subgroup
Klein 2024

Combination NRT
Events
0
Total
126

Text message-based intervention
Events
0
Total
126

Risk Ratio
M-H, Random, 95% CI
Not estimable

Risk Ratio
M-H, Random, 95% CI


0.1

0.2

0.5

1

2

5

10


Favours combination NRT

Favours text message-based intervention


Analysis 9.2: Vaping cessation at between 3 & 6 months


Study or Subgroup
Klein 2024

Combination NRT
Events
52
Total
126

Text message-based intervention
Events
45
Total
126

Risk Ratio
M-H, Random, 95% CI
1.16 [0.84 , 1.58]

Risk Ratio
M-H, Random, 95% CI


0.5

0.7

1

1.5

2


Favours text message-based intervention

Favours combination NRT


Analysis 9.3: Number of participants reporting AEs


Study or Subgroup
Klein 2024

Combination NRT
Events
33
Total
96

Text message-based intervention
Events
10
Total
87

Risk Ratio
M-H, Random, 95% CI
2.99 [1.57 , 5.70]

Risk Ratio
M-H, Random, 95% CI


0.1

0.2

0.5

1

2

5

10


Favours combination NRT

Favours text message-based intervention
