# Supplementary material for: Interventions for quitting vaping
Source: Cochrane Database Syst Rev. 2025 Jan 8;2025(1):CD016058. doi: 10.1002/14651858.CD016058.pub2 (PMC11706636; doi:10.1002/14651858.CD016058.pub2)
Supplement: Supplementary file 8 — Supplementary material 8 Data to be extracted from included studies. [file CD016058-SUP-08-other.html]

Data to be extracted from included studies.


# Supplementary material 8 to: Interventions for quitting vaping

Butler AR, Lindson N, Livingstone-Banks J, Notley C, Turner T, Rigotti NA, Fanshawe TR, Dawkins L, Begh R, Wu AD, Brose L, Conde M, Simonavičius E, Hartmann-Boyce J
  
https://doi.org/10.1002/14651858.CD016058.pub2

The material in this section has been supplied by the author(s) for publication under a Licence for Publication and the author(s) are solely responsible for the material. Cochrane has reviewed this material, but Cochrane has not copyedited, formatted or proofread. Cochrane accordingly gives no representations or warranties of any kind in relation to, and accepts no liability for any reliance on or use of, such material.

Back to top

# Data to be extracted from included studies.

We will extract the following information from the studies that meet our inclusion critera:

- Author
- Date and country of publication
- Study start and finish dates
- Study design
- Study location and setting
- Recruitment method
- Participant characteristics, to include measures of vaping/smoking history and baseline use of e-cigarettes in regards to content, device, and flavours; nicotine dependence measures; and PROGRESS+ indicators [1]
- Intervention and comparator(s) methods
- Intervention provider(s)
- Number of participants in each study arm
- Relevant outcomes measured
- Definitions of outcomes used
- Relevant assessment time points
- Numbers and proportions of participants changing their tobacco smoking behaviour between baseline and longest follow-up (i.e. continuing to smoke, taking up smoking, or stopping smoking)
- Numbers and proportions of participants abstinent from vaping at relevant follow-ups
- Numbers and proportions of participants who reported SAEs at relevant time point
- Numbers and proportions of participants who reported adverse events at relevant time point
- Number and proportions of participants vaping a substance other than nicotine at relevant follow-ups;
- Changes in relevant biomarkers
- Changes in weight;
- Changes in alcohol use status;
- Denominators for each outcome
- Loss to follow‐up at time points when relevant outcomes are measured
- Any reported analysis looking at vape characteristics as a moderator of relevant outcomes
- Information needed to assess risk of bias as specified below
- Funding source
- Authors’ declarations of interest
- Any additional comments

## References

1. O'Neill J, Tabish H, Welch V, Petticrew M, Pottie K, Clarke M et al. Applying an equity lens to interventions: using PROGRESS ensures consideration of socially stratifying factors to illuminate inequities in health. Journal of Clinical Epidemiology 2014;67(1):56-64. [DOI: 10.1016/j.jclinepi.2013.08.005]
